# Hyperactivation of Nrf2 increases stress tolerance at the cost of aging acceleration due to metabolic deregulation

**DOI:** 10.1111/acel.12845

**Published:** 2018-12-10

**Authors:** Eleni N. Tsakiri, Sentiljana Gumeni, Kalliopi K. Iliaki, Dimitra Benaki, Konstantinos Vougas, Gerasimos P. Sykiotis, Vassilis G. Gorgoulis, Emmanuel Mikros, Luca Scorrano, Ioannis P. Trougakos

**Affiliations:** ^1^ Department of Cell Biology and Biophysics Faculty of Biology National & Kapodistrian University of Athens Athens Greece; ^2^ Department of Pharmaceutical Chemistry Faculty of Pharmacy National & Kapodistrian University of Athens Athens Greece; ^3^ Biomedical Research Foundation Academy of Athens Athens Greece; ^4^ Service of Endocrinology, Diabetology and Metabolism Lausanne University Hospital Lausanne Switzerland; ^5^ Department of Histology and Embryology School of Medicine National & Kapodistrian University of Athens Athens Greece; ^6^ Faculty of Biology, Medicine and Health University of Manchester Manchester UK; ^7^ Department of Biology, Venetian Institute of Molecular Medicine, Dulbecco‐Telethon Institute University of Padua Padova Italy

**Keywords:** aging, insulin/IGF‐like, metabolism, mitostasis, Nrf2, proteostasis

## Abstract

Metazoans viability depends on their ability to regulate metabolic processes and also to respond to harmful challenges by mounting anti‐stress responses; these adaptations were fundamental forces during evolution. Central to anti‐stress responses are a number of short‐lived transcription factors that by functioning as stress sensors mobilize genomic responses aiming to eliminate stressors. We show here that increased expression of nuclear factor erythroid 2‐related factor (Nrf2) in *Drosophila *activated cytoprotective modules and enhanced stress tolerance. However, while mild Nrf2 activation extended lifespan, high Nrf2 expression levels resulted in developmental lethality or, after inducible activation in adult flies, in altered mitochondrial bioenergetics, the appearance of Diabetes Type 1 hallmarks and aging acceleration. Genetic or dietary suppression of Insulin/IGF‐like signaling (IIS) titrated Nrf2 activity to lower levels, largely normalized metabolic pathways signaling, and extended flies’ lifespan. Thus, prolonged stress signaling by otherwise cytoprotective short‐lived stress sensors perturbs IIS resulting in re‐allocation of resources from growth and longevity to somatic preservation and stress tolerance. These findings provide a reasonable explanation of why most (if not all) cytoprotective stress sensors are short‐lived proteins, and it also explains the build‐in negative feedback loops (shown here for Nrf2); the low basal levels of these proteins, and why their suppressors were favored by evolution.

## 1 INTRODUCTION

The viability of metazoans largely depends on their ability to regulate metabolic processes in order to produce energetic molecules, as well as on their capacity to mount anti‐stress responses. These processes are mostly regulated by short‐lived sensors (mainly transcription factors) which in cases of disturbing departures from the optimal levels set by evolution, trigger genomic responses aiming to restore normal cellular functionality (López‐Otín, Blasco, Partridge, Serrano, & Kroemer, 2013). At the whole organism level, these responses require complex co‐regulation and wiring of cell‐autonomous and non‐autonomous mechanisms (Kaushik & Cuervo, 2015). The efficiency of these processes declines during aging leading to increased morbidity and mortality (López‐Otín et al., 2013).

Nevertheless, it is nowadays evident that lifespan can be prolonged by genetic and/or dietary interventions. Specifically, several studies in model organisms have shown that longevity can be increased by caloric restriction (CR) and/or genetic interventions that reduce the activity of energy‐ and/or nutrient‐sensing signaling pathways including, the insulin/IGF‐like signaling (IIS) and the Target of Rapamycin (ToR)/ribosomal protein S6 kinase signaling pathways (López‐Otín et al., 2013). It is believed that CR results in activation of cellular defenses that delay the rate of age‐related accumulation of stressors and/or damaged biomolecules in cells (Fontana & Partridge, 2015).

Central to cell defense pathways are the networks of DNA (DDR) and proteome (PDR) damage responses; the latter ensure proteome stability during proteotoxic stress by activating the proteostasis network (PN). Key components of the PN are the protein synthesis and trafficking machineries; the endoplasmic reticulum (ER) unfolded protein responses (UPR^ER^), the molecular chaperones and the two main degradation machineries, namely the Autophagy Lysosome‐ (ALP) and the Ubiquitin‐Proteasome (UPP) pathways. ALP is mostly involved in the degradation of protein aggregates and damaged or non‐functional organelles and it is subject to negative ToR regulation (Kaushik & Cuervo, 2015), while UPP ensures protein synthesis quality control and degrades normal short‐lived or non‐repairable misfolded proteins (Tsakiri & Trougakos, 2015). The 26S eukaryotic proteasome comprises a 20S core particle (CP) bound to 19S regulatory particles (RP). The 20S CP consists of four stacked heptameric rings (two α type surrounding two β type) that form a barrel‐like structure; the caspase‐ (C‐L), trypsin‐ (T‐L), and chymotrypsin‐ (CT‐L) like proteasome enzymatic activities are located at the β1, β2, and β5 subunits, respectively. The 19S RP is involved in substrate recognition, deubiquitination, unfolding, and translocation into the 20S CP (Tsakiri & Trougakos, 2015). Notably, the functionality of both the anti‐stress responses module and the PN declines during in vivo aging contributing to age‐related diseases (Kaushik & Cuervo, 2015); in support, disruption of proteostasis in young *Drosophila *flies accelerated aging (Tsakiri, Sykiotis, Papassideri, Terpos, et al., 2013). On the other hand, increased activities of proteostatic modules have been correlated with increased longevity. Consistently, forced reinvestment of resources from the germ line to the soma in *C. elegans *resulted in elevated somatic proteasome activity, effective clearance of damaged proteins and increased longevity (Vilchez et al., 2012). Also, *Drosophila *reproductive tissues age at a lower rate than the soma due to their higher proteasome activities that prevent age‐related proteome instability (Tsakiri, Sykiotis, Papassideri, Gorgoulis, et al., 2013).

The network of stress‐responsive cellular sensors comprises several short‐lived proteins, including the transcription factors forkhead box O (Foxo), heat shock factor‐1 (Hsf1) and nuclear factor erythroid 2‐related factor (Nrf2). Nrf2 plays a central role in cell responses against oxidative and xenobiotic damage (Sykiotis & Bohmann, 2010). Specifically, oxidative stress abrogates the Keap1‐mediated proteasomal degradation of Nrf2, which in turn accumulates in the nucleus and heterodimerizes with a small musculoaponeurotic fibrosarcoma (Maf) protein on antioxidant response elements (AREs) to upregulate the expression of antioxidant and phase II genes (Sykiotis & Bohmann, 2010). Nrf2 activity is also regulated by several protein kinases, including inhibition by Glycogen synthase kinase 3β (Gsk3β) and tyrosine kinase Fyn (Pitoniak & Bohmann, 2015).

We are using *Drosophila *flies as a model organism to study the functional implication of stress sensors in PN regulation, organismal physiology, and aging. We reported recently that cap “*n*” collar isoform‐C (CncC; the Nrf2 ortholog in *Drosophila*) regulates antioxidant responses and proteasome functionality in flies’ tissues, and that its functionality declines in the aged soma. Notably, high levels of CncC/Nrf2 expression or endogenous activity [induced by Keap1 knock down (KD)] accelerated aging despite concomitant proteasome activation (Tsakiri, Sykiotis, Papassideri, Terpos, et al., 2013).

We report here that increased Nrf2 activity in flies enhanced stress tolerance and activated proteostatic modules in a dose‐dependent manner. However, while mild Nrf2 upregulation extended lifespan, high Nrf2 expression levels reduced longevity due to reprogramming of cellular bioenergetics that resulted in the appearance of Diabetes Type 1‐like (DT1) phenotypes; these toxic effects were alleviated by downregulating IIS.

## 2 RESULTS

### 2.1 CncC/Nrf2 overexpression (OE) activates cytoprotective proteostatic modules in a dose‐dependent manner and renders flies resistant to stressors

RU486 treatment caused no significant effects on flies’ proteostatic modules and longevity (Supporting information Figure S1). Also, the *cncC/nrf2*, *maf‐S, *and *keap1 *genes are expressed in all flies’ tissues assayed (Supporting information Figure S2); and thus, ubiquitous CncC/Nrf2 OE or activation (by *keap1 *KD) resembles physiological responses to systemic stress. Ubiquitous OE of CncC/Nrf2 (Supporting information Figure S3A1) with the RU486‐inducible GeneSwitch Tubulin driver resulted at doses higher than 10 μM of RU486 in lethality during late larval/early pupal stages. Development was successfully completed at RU486 concentrations lower than 10 μM or in non‐RU486‐treated transgenic larvae, despite low levels of leaky transgene expression (Supporting information Figure S3A2). Thus, Nrf2‐mediated effects are likely dose dependent. Analyses in isogenic CncC/Nrf2 transgenic adult flies showed that RU486 treatment induced dose‐dependent upregulation of CncC/Nrf2 (to a maximum of ~4‐fold) and of proteasome subunits expression (Supporting information Figure S3B), as well as of proteasome assembly (Supporting information Figure S3C1) and proteasome activities (Supporting information Figures S3C2, S3C3); notably, increased CncC/Nrf2 activity coincided with proteome over‐ubiquitination (Supporting information Figure S3B). Also, increasing doses of RU486 upregulated proteasomal genes in a dose‐dependent manner (Supporting information Figure S3D). Other CncC/Nrf2 transcriptional targets, for example, the antioxidant genes *gstd1 *and* trxr-1* or the CncC/Nrf2 suppressor gene *keap1*, were also induced in a dose‐dependent manner but at higher RU486 doses, while the *maf‐S *gene showed a unique response pattern; thus, not all Nrf2‐targeted enhancers are equivalent. CncC/Nrf2 OE suppressed reactive oxygen species (ROS) or carbonylated proteins (DNPs) in middle‐aged flies, while both ROS and DNPs were accumulating following suppression of CncC/Nrf2 activity (Supporting information Figures S3E, S3F).

**Figure 1:**
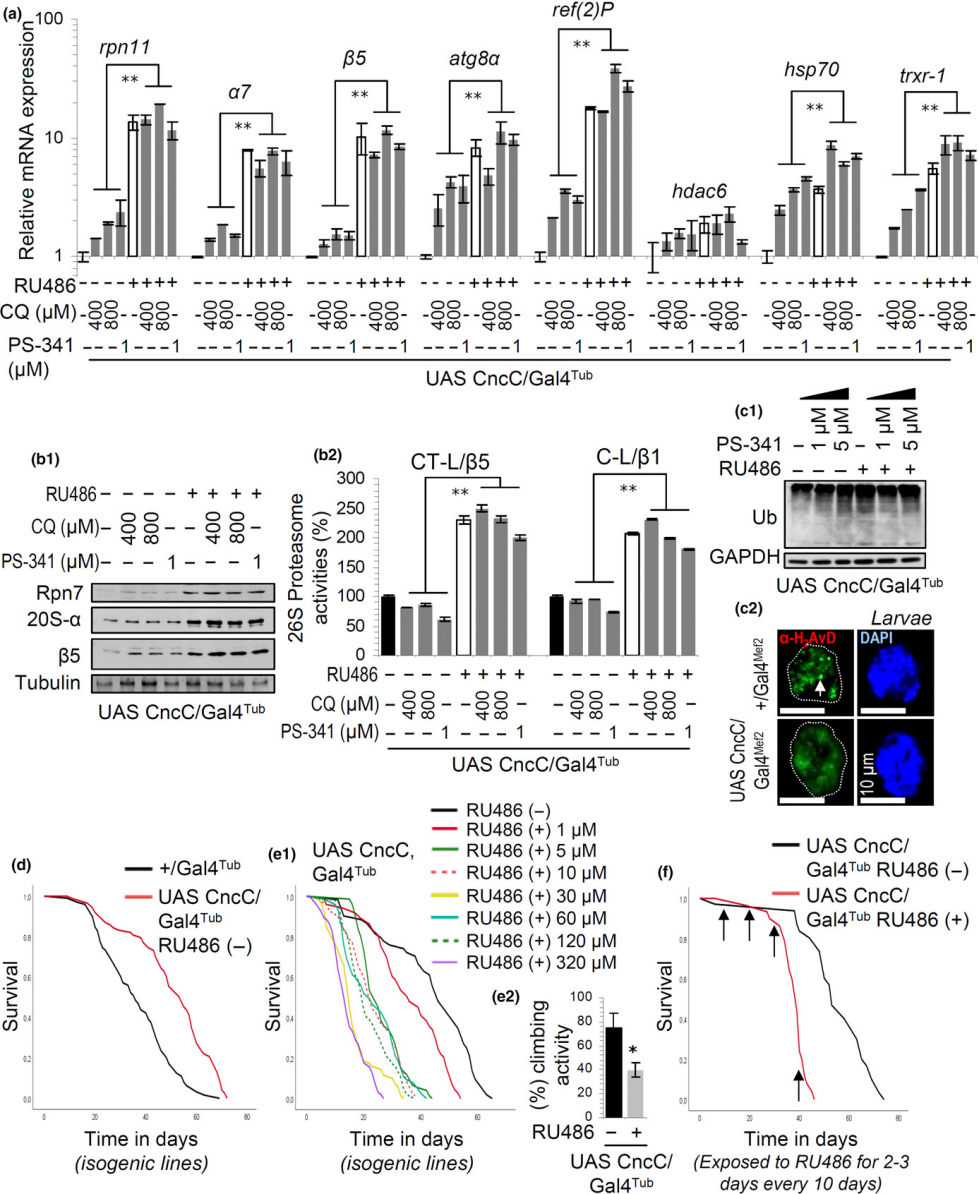
Sustained CncC/Nrf2 overactivation in adult flies enhances tissues’ responses to stress; yet, it causes a significant decrease in lifespan. (a) Relative expression of rpn11, α7, β5, atg8α, ref(2)P, hdac6, hsp70 and trxr‐1 genes in control flies; after exposing flies to the shown concentrations of Chloroquine (CQ) or PS‐341, and/or CncC/Nrf2 induction by RU486. (b) Immunoblot analyses (b_1_) or relative (%) 26S proteasome activities (b_2_) in flies’ tissues after treating control or CncC/Nrf2 overexpressing flies as in (a); samples in (b_1_) were probed with antibodies against proteasomal subunits Rpn7, 20S‐α and β5. (c_1_) Immunoblots of samples from non‐induced or CncC/Nrf2 overexpressing PS‐341 treated flies probed with antibodies against ubiquitin (Ub). (c_2_) CLSM visualization of H2AvD immunofluorescence staining in larvae muscle tissues nuclei of the shown genotypes following UV exposure; samples were counterstained with DAPI (white arrow denotes H2AvD positive nuclear foci). (d, e) Longevity curves of control (+/Gal4_Tub_) or CncC/Nrf2 overexpressing isogenic transgenic flies cultured in the absence of RU846 (d) or with the indicated RU486 concentrations (e_1_); in (e_2_) the relative locomotion activity of middle‐aged CncC/Nrf2 overexpressing flies (vs. controls) is shown. (f) Longevity curves of transgenic flies after short‐term (2–3 days every 10 days; marked by arrows) exposure to RU486. If not otherwise indicated, flies were exposed to 320 μM RU486. In (a–c_1_) young transgenic flies were exposed to RU486 for 3 days. In (b_2_) control values were set to 100%. Gene expression in (a) was plotted vs. control set to 1 (rp49 gene was used as reference). Tubulin (b_1_) or GAPDH (c_1_) probing were used to demonstrate equal protein loading. Bars, ±*SD*; **p < 0.01

CncC/Nrf2 OE in adult flies’ tissues maximized the upregulation of proteostatic or antioxidant response genes (Figure 1a) and proteins (Figure 1b_1_), as well as that of proteasomal activities (Figure 1b_2_), after chloroquine‐ (CQ; lysosome inhibitor) or PS‐341 (proteasome inhibitor)‐mediated proteotoxic stress. It also suppressed total proteome over‐ubiquitination after proteasome inhibition (Figure 1c_1_), further supporting proteasome activation. CncC/Nrf2 overexpressing larvae or flies were in the short‐term resistant to oxidative, proteotoxic (not shown) or genotoxic stress (Figure 1c_2_); however, only mild CncC/Nrf2 induction (i.e., leaky expression in the absence of RU486) increased flies’ longevity (Figure 1d), whereas higher CncC/Nrf2 expression levels decreased flies’ lifespan, as well as the locomotion activity of middle‐aged flies (Figure 1e). The progeria effect was evident even after short periods of CncC/Nrf2 induction in adult flies (Figure 1f) (Supporting information Table S1). Similar effects on proteostatic modules and flies’ longevity were also noted following Keap1 KD (Supporting information Figure S4) or after CncC/Nrf2^Δ1‐87 ^OE (Supporting information Figure S5); the latter refers to a truncated CncC/Nrf2 form that lacks the N‐terminus 87 amino acids (Supporting information Figure S3A1) containing the ER‐localization NHB1 domain (Pitoniak & Bohmann, 2015). Thus, prolonged CncC/Nrf2 overactivation is toxic.

In the absence of stress, inducible CncC/Nrf2 OE in adult flies promoted the upregulation of additional proteostatic modules, including *atg8α*, *ref(2)P *(the fly ortholog of p62/Sequestosome‐1), *hdac6*, *hsp70, *and *grp78 *(ER stress‐related protein) genes (Supporting information Figure S6A1); and Atg8α, Ref(2)P proteins in adult (Supporting information Figure S6A2) and larval (Supporting information Figure S6A3) tissues. CncC/Nrf2 induction in larvae resulted (as in the adult) in increased proteome ubiquitination, which was, however, less intense compared to CncC/Nrf2 KD (Supporting information Figure S6B) that has been shown (Tsakiri, Sykiotis, Papassideri, Terpos, et al., 2013) to suppresses proteasomal activity. These findings prompted us to perform high‐resolution iTRAQ proteomics in somatic tissues of CncC/Nrf2 overexpressing adult flies (Supporting information Tables S2, S3). Results revealed that CncC/Nrf2 positively regulates several proteostatic modules including Hsp26 and Hsc20 chaperones, the Ubiquitin (Ub) carboxyl‐terminal hydrolase Uch‐L5, the ER‐associated degradation chaperone Ter94 (the fly ortholog of human valosin‐containing protein, VCP/p97) and its adaptor Ufd1/Npl4, as well as the Ub binding and autophagosome assembly‐related protein p47, and the E1‐Ub and SUMO activating protein Aos1 (Supporting information Figure S6C). Gene expression (Supporting information Figures S6D, S6E) or chromatin immunoprecipitation (ChIP) (Supporting information Figure S7) studies showed that CncC/Nrf2 is likely a direct regulator of the expression levels of these genes. Gene Ontology analyses of the proteomics data revealed that CncC/Nrf2 OE also upregulated peptides involved in chromatin replication, stability, and silencing (Supporting information Table S2), indicating that it also affects modules involved in genome stability. In conclusion, while mild CncC/Nrf2 activation can be in the long‐term beneficial, CncC/Nrf2 overactivation is paradoxically toxic in the absence of evident biomolecular damage and in spite of mobilizing cytoprotective modules that confer stress resistance.

### 2.2 Persistent high CncC/Nrf2 expression levels alter mitochondrial bioenergetics and deregulate metabolic pathways resulting in DT1‐like phenotypes

**Figure 2:**
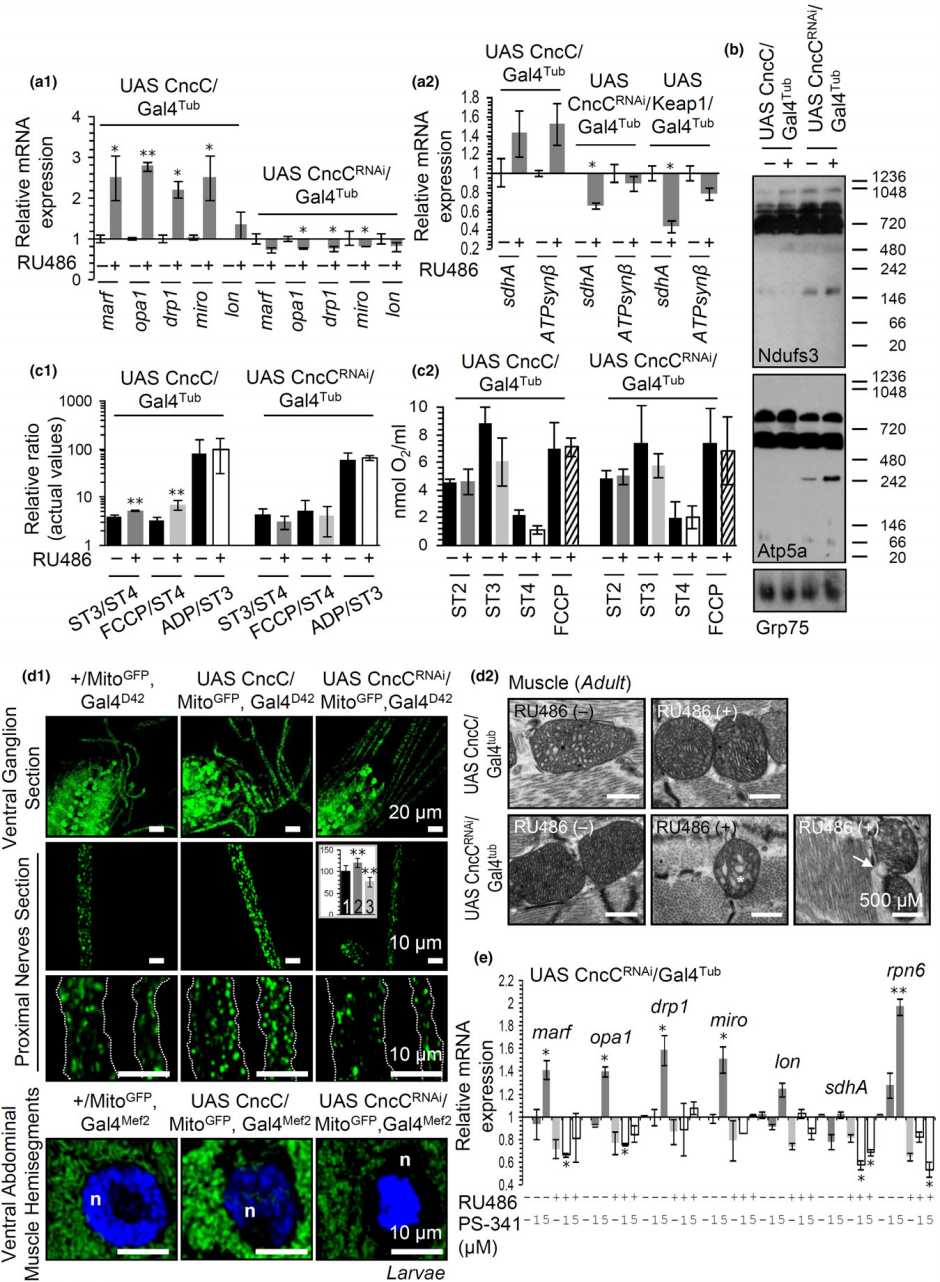
Persistent CncC/Nrf2 OE upregulates mitochondrial dynamics and bioenergetics. (a) Relative expression of marf, opa1, drp1, miro and lon (a_1_), as well as, of sdhA and ATPsynβ (a_2_) genes in the indicated genotypes. (b) Immunoblots after BN‐PAGE, for the analysis of mitochondrial RCS assembly, and probing with antibodies against Ndufs3 (complex I) and Atp5a (complex V); mitochondria were isolated after inducible CncC/Nrf2 OE or KD. (c) Relative mitochondrial ST3/ST4, FCCP/ST4, and ADP/ST3 ratios (c_1_) or ST2, ST3, ST4, and FCCP values (c_2_) after inducible CncC/Nrf2 OE or KD. (d) CLSM (d_1_) or EM (d_2_) visualization of mitochondria in the nervous system (d_1_; upper panels) and muscle [d_1_; lower panels, (n) nuclei], (d_2_) after CncC/Nrf2 OE or KD. In (d_2_) the white star and arrow indicate disrupted mitochondrial cristae and outer membrane, respectively. Insert in (d_1_) indicates quantitation of stained mitochondria (1: +/Mito^GFP^, Gal4^D42^; 2: UAS CncC/Mito^GFP^, Gal4^D42^; 3: UAS CncC^RNAi^/Mito^GFP^, Gal4^D42^). (e) Relative expression of mitochondrial marf, opa1, drp1, miro, lon, sdhA, and proteasomal rpn6 genes following treatment with PS‐341 after inducible CncC/Nrf2 KD vs. controls. Young flies were exposed to 320 μM RU486 for 7 days. Gene expression was plotted vs. the respective control set to 1. Grp75 (Hsc70‐5) probing (b) and rp49 gene expression (a, e) were used as reference. Bars, ±*SD*; *p < 0.05; **p < 0.01

Nano‐LC‐ESI‐MS/MS proteomics analyses after Ub immunoprecipitation in samples from CncC/Nrf2 overexpressing flies (Supporting information Tables S4, S5) revealed differential accumulation of several over‐ubiquitinated proteins including pyruvate kinase (Pyk), cytochrome c oxidase subunits 2 (Cox2) and 5B (CoVb), as well as succinate‐CoA ligase subunit beta (Skap) indicating that CncC/Nrf2 OE affects mitostatic pathways. Indeed, CncC/Nrf2 activation upregulated genes involved in mitochondrial dynamics (*marf*, *opa1 *and *drp1*), cristae stability (*opa1*), mitochondria motility (*miro*), and energetics (*sdhA*, *ATPsynβ*) (Figure 2a). Also, CncC/Nrf2 OE increased the assembly of mitochondrial complexes I and V respiratory chain supercomplexes (RCS); conversely, increased disassembly of these RCS was observed in CncC/Nrf2 KD flies (Figure 2b). Moreover, CncC/Nrf2 OE increased mitochondrial respiratory control (ST3/ST4) and FCCP/ST4 ratios (Figure 2c_1_), suggesting increased substrate oxidation, tight mitochondrial coupling, and reduced proton leak. Nevertheless, closer inspection of the ST2, ST3, ST4, and FCCP values revealed that ST3 tends to decrease after CncC/Nrf2 OE (Figure 2c_2_), indicating lower rates of ADP phosphorylation. In line with RCS disassembly, CncC/Nrf2 KD flies showed a tendency toward reduced mitochondrial coupling and increased proton leak (Figure 2c_1_), as reflected in decreased ST3 and increased ST4 values, respectively (Figure 2c_2_). CncC/Nrf2 OE in larval nervous system or muscle (Figure 2d_1_; see also insert graph) led to enhanced Mito^GFP ^reporter staining indicating a denser mitochondrial network; again, opposite effects were observed after CncC/Nrf2 KD. EM analyses showed that CncC/Nrf2 OE caused no significant effects on mitochondria fine structure, while CncC/Nrf2 KD disrupted cristae stability and outer membrane integrity (Figure 2d_2_). CncC/Nrf2 is also functionally involved in the upregulation of mitostatic genes upon proteasome inhibition as this effect was abolished upon CncC/Nrf2 KD (Figure 2e). Thus, CncC/Nrf2 modulates mitochondrial dynamics and energetics, and it induces mitochondrial genes upon proteome instability.

**Figure 3:**
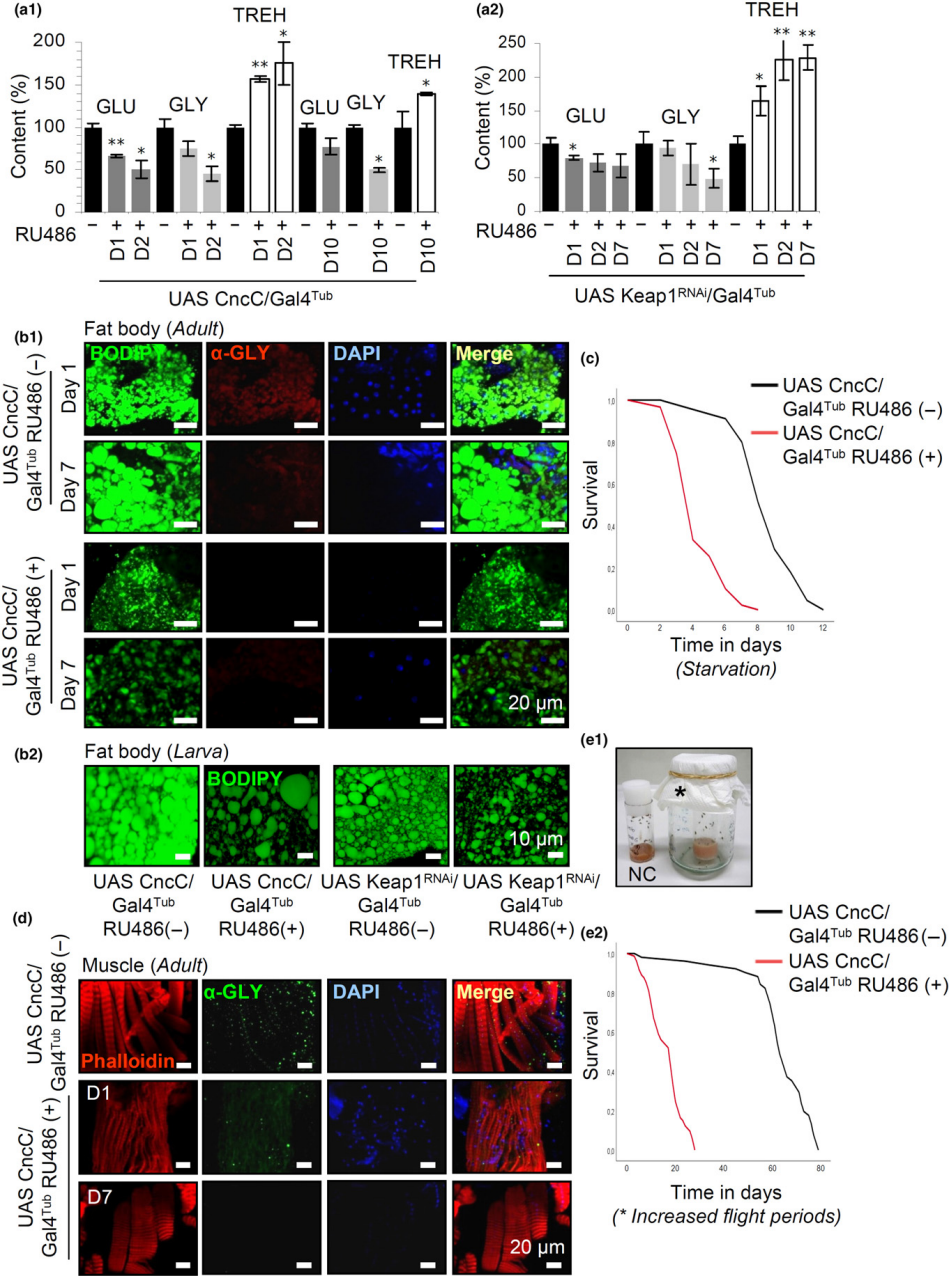
Prolonged CncC/Nrf2 activation results in phenotypes that are hallmarks of DT1. (a) Relative (%) content (vs. controls) of GLU, GLY, and TREH in flies’ somatic tissues after CncC/Nrf2 OE (a_1_) or Keap1 KD (a_2_); flies were treated with RU486 for 1 (D1) up to 10 (D10) days. (b) CLSM visualization of fat bodies after BODIPY staining of transgenic CncC/Nrf2 overexpressing (or not) adult flies (b_1_, exposed to RU486 for 1 or 7 days) or larvae (b_2_, genotypes are indicated). Samples in (b_1_) were also stained by immunofluorescence for α‐GLY and counterstained with DAPI. (c) Longevity curves of starved control or CncC/Nrf2 overexpressing adult flies. (d) CLSM visualization following immunofluorescence staining of adult flies muscle tissues with an α‐GLY antibody (flies were treated with RU486 for 1 or 7 days); samples were counterstained with Phalloidin and DAPI. (e) Longevity curves of control or CncC/Nrf2 overexpressing adult flies (e_2_) after facilitating increased flying periods (e_1_); NC denotes normal culturing conditions and star the enlarged vials used to enable increased flying periods. Larvae in (b_2_) were exposed to 320 μM RU486 for 4 days. In (a), control values were set to 100%. Bars, ±*SD*; *p < 0.05; **p < 0.01

Downstream analyses in metabolic pathways showed that prolonged CncC/Nrf2 activation (after CncC/Nrf2 OE or Keap1 KD) resulted in gradual decrease of glucose (GLU) and glycogen (GLY) content in flies’ tissues. In contrast, CncC/Nrf2 overactivation in flies resulted in progressively increased levels of trehalose (TREH; a fat body synthesized sugar that circulates in flies’ hemolymph) indicating a hyperglycemic state (Figure 3a). Similar to GLU synthesis in the mammalian liver, TREH synthesis in the fly fat body is coupled to fatty acid oxidation and to reduced rates of glycolysis and glycogenesis. Indeed, sustained CncC/Nrf2 activation reduced GLY staining in adult flies’ fat body (Figure 3b_1_). Also, BODIPY staining of fat body lipid droplets in adult flies (Figure 3b_1_) or larvae (Figure 3b_2_) documented extensive lipolysis; this is consistent with downregulation of fatty acid synthase 1 observed in proteomics analyses (Supporting information Table S2). CncC/Nrf2 overexpressing flies were sensitive to starvation (Figure 3c) and showed reduced GLY staining in muscles (Figure 3d), while exposure of flies to increased flight periods further accelerated aging and premature death (Figure 3e). Similar late DT1‐like phenotypes were found after prolonged CncC/Nrf2^Δ1‐87 ^OE (Supporting information Figure S8). These DT1‐like phenotypes suggest an extensive reprogramming of metabolic signaling due to CncC/Nrf2 overactivation.

NMR‐based metabolomics analysis (Supporting information Figure S9, Supporting information Table S6) in adult flies verified these observations, as CncC/Nrf2 OE increased ATP and TREH levels, whereas it decreased maltose, GLU, and GLU‐1‐phosphate (intermediate for GLY synthesis). In addition, triglycerides, and polyunsaturated fatty acids, the amino acids Ala and Pro, lactate (these metabolites are used as energetic substrates during active muscle contraction or during fasting), and several respiratory substrates of the Krebs cycle (e.g. succinic, fumaric, and malic acid) were significantly reduced. Notably, citric acid levels were increased indicating that glycolysis is progressively switched off. We also observed that branched proteinogenic amino acids (e.g., Leu, Ile, and Val) tend to increase, indicating uncontrolled protein catabolism that has been recognized as a marker of insulin resistance or deficiency. These effects are likely compounded by reduced protein synthesis since our proteomics analyses showed downregulation of the ribosomal assembly‐related Rpl24 and Rpl17 peptides (Supporting information Table S2) and coincide with reduced locomotion activities of middle‐aged flies, as well as with enhanced death rates after increased periods of flight (see above) suggesting that protein breakdown promotes diabetic myopathy‐like phenotypes. The primary benign role of CncC/Nrf2 is, however, still evident in the upregulation of cytoprotective metabolites like taurine and the neurotransmitter gamma‐aminobutyric acid (GABA); the latter inhibits brain insulin‐producing cells (IPCs) in flies’ (Rajan & Perrimon, 2016). Further, CncC/Nrf2 OE reduced the levels of the neurotoxic metabolite 3‐hydroxykynurenine, as well as of Met‐sulfoxide. Notably, several of the aforementioned metabolites (e.g., TREH, ATP, lactate or citric acid) were regulated in the opposite direction after CncC/Nrf2 KD (Supporting information Table S6).

**Figure 4:**
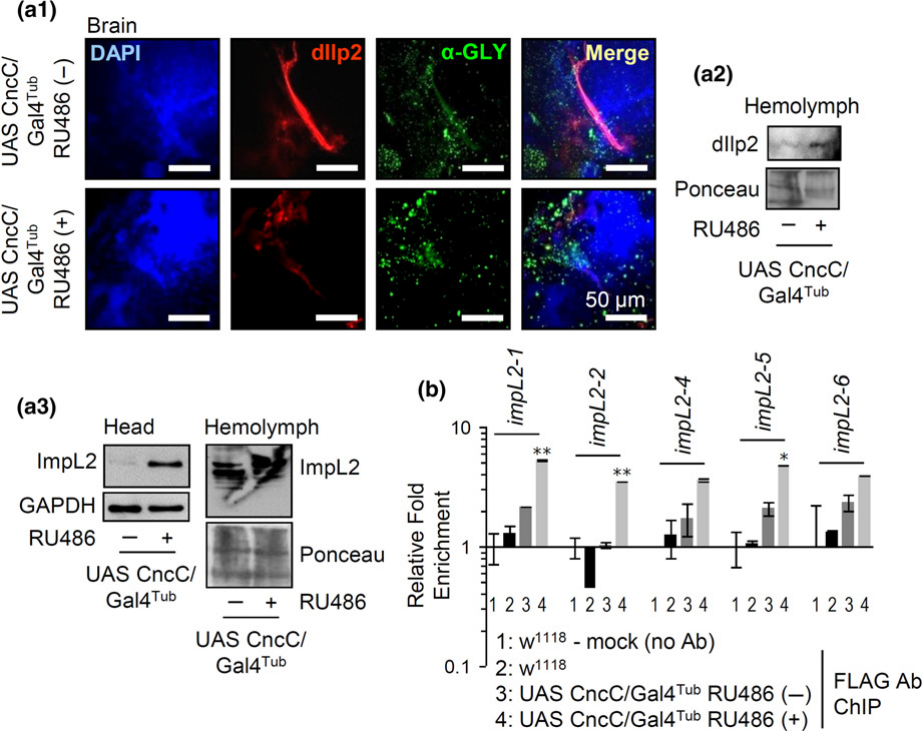
CncC/Nrf2 OE modulates dIlp2 levels in brain IPC cells and in the hemolymph, and it upregulates Impl2 (a dIlp2 inhibitor) in flies’ head tissues and in the hemolymph. (a_1_) CLSM visualization following immunofluorescence staining of isolated brains from CncC/Nrf2 overexpressing (or not) flies with antibodies against dIlp2 and α‐GLY; samples were counterstained with DAPI. (a_2_, a_3_) Immunoblot analyses of hemolymph or dissected head tissues samples probed with antibodies against dIlp2 (a_2_) or ImpL2 (a_3_). (b) ChIP assays showing CncC/Nrf2 binding to *impL2* regulatory regions; experimental controls were the u1, rp49, and gapdh genes and showed no CncC/Nrf2 binding. Young flies were exposed to 320 μM RU486 for 7 days. Pounceau staining (a_2_, a_3_) or GAPDH (a_3_) were used as input references. Bars, ±*SD*; *p < 0.05; **p < 0.01

The progressive impact of CncC/Nrf2 overactivation on metabolic pathways was also evident after gene expression analyses during days 1, 2, 7, and 20, post‐CncC/Nrf2 OE (Supporting information Figure S10A; upper panel). We observed upregulation of the gluconeogenic *g6p *and *pepck *genes, while enzymes involved in glycolysis or Krebs cycle regulation (*pyk*, *pdp*, *pdk*) were only mildly induced in the first 1–2 days of CncC/Nrf2 OE. By analyzing the expression levels of genes involved in IIS, we found a late downregulation of *insulin‐like peptide 2* (dIlp2; secreted from IPCs by cell‐autonomous GLU sensing), along with *dilp6 *(secreted during fasting states from the fat body to repress dIlp2 secretion from the brain) and *impL2 *(a muscle‐secreted factor that inhibits dIlp2 activity) upregulation. Since dIlps function as ligands for the sole insulin receptor (InR) in the fly genome, the observed upregulation of *inr *and of its downstream effector *pdpk1 *are likely a late compensatory response due to reduced IIS. Also, in line with increased lipolysis, CncC/Nrf2 OE resulted in upregulation of the *atgl *and *tgl *lipases genes. Similar genomic effects were evident after Keap1 KD and were (in most cases) not inverted after CncC/Nrf2 KD (Supporting information Figure S10A; middle, lower panels). Interestingly, at 20 days post‐CncC/Nrf2 activation the expression pattern of several of the metabolic genes assayed was inverted, suggesting that the initial genomic responses eventually collapse. As CncC/Nrf2 activation induced dIlp2 inhibitors, (e.g., the *dilp6 *and *impL2 *genes) we then asked whether CncC/Nrf2 directly regulates IIS modulators. We found reduced dIlp2 staining in CncC/Nrf2 overexpressing flies’ brain IPCs (Figure 4a_1_), likely due to TREH accumulation‐mediated dIlp2 over‐secretion in hemolymph (Figure 4a_2_). Furthermore, ImpL2 was found to accumulate in head tissues and in the hemolymph (Figure 4a_3_) of CncC/Nrf2 overexpressing flies, while ChIP analyses showed the direct binding of CncC/Nrf2 in *impL2* regulatory elements (Figure 4b) indicating a direct regulation of the *impL2 *gene by CncC/Nrf2. OE of CncC/Nrf2 also upregulated (apart from *keap1*; see above) its other inhibitor, namely *sgg/gsk3 *(Shaggy, the fly ortholog of mammalian Gsk3) (Chatterjee, Tian, Spirohn, Boutros, & Bohmann, 2016; Tsakiri et al., 2017) (Supporting information Figure S10B) indicating that as Nrf2 network evolved in higher metazoans one of its major functions is to limit its own activity (Supporting information Figure S11A). As deduced from Supporting information Figure S11A, an Nrf2‐mediated decrease in IIS should trigger late Foxo and ALP activation. Indeed, we found that CncC/Nrf2 activation resulted in Foxo accumulation and reduced Akt phosphorylation; it also increased phosphorylation of AMPKα (Supporting information Figure S11B1) and suppressed an inhibitory (S^21^/S^9^) Sgg/Gsk3 phosphorylation (Supporting information Figure S11B2). We further observed higher expression levels of the autophagic *atg8α* (see above) and *atg6 *(Supporting information Figure S11C) genes and, by using mCherry‐Atg8α line, of the Atg8α protein (Supporting information Figure S11D). Consistently, we noted higher activity of lysosomal cathepsins B, L (Supporting information Figure S11E; cathepsin D induction was also found in proteomics analyses) and enhanced expression of a GFP‐Lamp1 reporter in the nervous system (Supporting information Figure S11F) indicating upregulated chaperon mediated autophagy. We suggest that although ALP activation may enhances resistance to stressors, it likely exacerbates the increased catabolism‐related side effects.

The DT1‐like phenotypes were also evident after muscle‐targeted CncC/Nrf2 activation. Specifically, CncC/Nrf2 OE by a strong muscle‐specific driver (Mef2) was lethal at late larval/early pupa stages. Analyses in transgenic larvae (while viable) showed the induction of proteostatic and metabolic genes (Supporting information Figure S12A), along with the activation of proteasomal peptidases and lysosomal cathepsins (Supporting information Figure S12B). Transgenic larvae or pupae were significantly growth‐retarded (Supporting information Figure S12C) and GLY stores in the larval muscles were depleted (Supporting information Figure S12D). Also, muscle‐targeted CncC/Nrf2 OE caused a lipolytic effect in larvae fat body (Supporting information Figure S12E), indicating a cell non‐autonomous systemic organ effect. By using a weaker muscle‐specific driver (MHC), we found that development was concluded with only mild larval growth retardation and fat body lipolytic effects (Supporting information Figure S12F). Yet, middle‐aged flies developed a “wings up” phenotype (Supporting information Figure S12G), indicating that they were unable to maintain flight muscle contraction. Given the fact that wing beat frequency is closely correlated with oxygen consumption and directly reflects the rate of ATP hydrolysis and glycolytic flux, we concluded that even mild prolonged CncC/Nrf2 induction eventually depletes energetic molecules in muscles. Beyond the shown CncC/Nrf2 OE‐mediated metabolic defects, our iTRAQ proteomic analyses revealed the downregulation of proteins involved in flies’ courtship songs, mating, and vitellogenesis (Supporting information Tables S2, S3), indicating that persistent activation of alarm pathways and stress signaling‐mediated suppression of IIS also downregulates reproductive machineries.

### 2.3 Knocking down effectors of nutrient‐sensing pathways alleviates CncC/Nrf2 OE‐mediated toxicity

**Figure 5:**
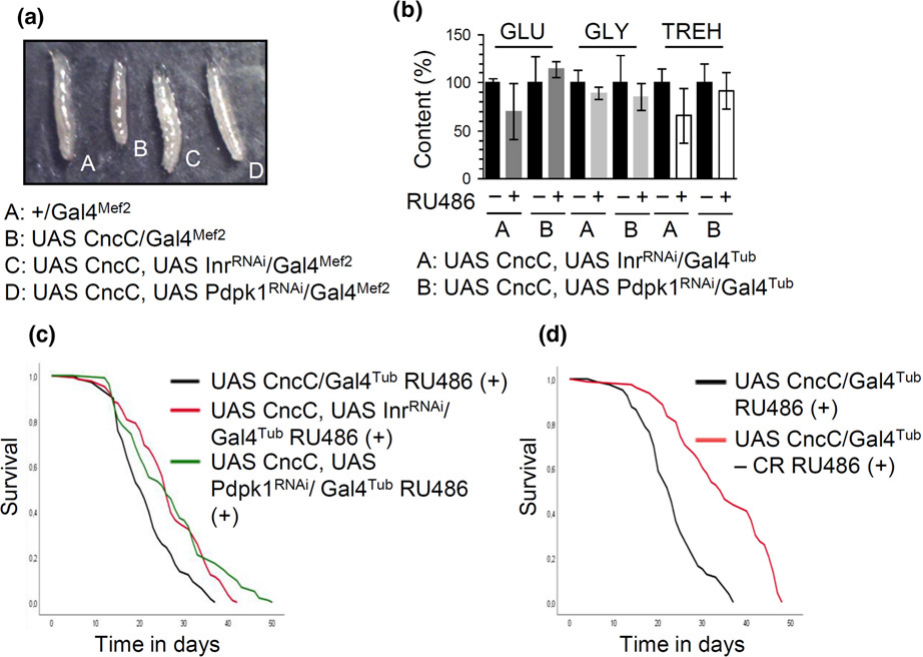
Suppressing IIS partially rescues the CncC/Nrf2 overactivation‐mediated effects on larvae growth, metabolic pathways, and adult flies’ longevity. (a) Stereoscope viewing of 3rd instar control (+/Gal4^Mef2^) or transgenic larvae expressing the indicated transgenes. (b) Relative (%) content (vs. controls) of GLU, GLY, and TREH in the shown transgenic lines’ somatic tissues. (c) Longevity curves of the indicated transgenic lines. (d) Longevity curves of CncC/Nrf2 overexpressing flies exposed (or not) to CR. Flies were exposed to 320 μM RU486. In (b) young mated flies were treated with RU486 for 7 days; control values were set to 100%. Bars, ±*SD*

As metabolic syndrome mostly relates to increased IIS (e.g., due to obesity) which would result in Nrf2 activation (Supporting information Figure S11A), we sought to moderate the CncC/Nrf2 OE‐mediated diabetes‐like phenotypes by modulating upstream IIS effectors or other downstream components of the pathway. Reduction of IIS is expected to activate ALP, Gsk3 (a CncC/Nrf2 inhibitor), and suppress glycogen synthase (Gys; the rate‐limiting enzyme of glycogenesis). We hypothesized that early constant downregulation of IIS or modulation of its end points (e.g., Atg8α OE or Gys KD) will suppress aberrant CncC/Nrf2 activity and/or will provide energetic biomolecules (e.g., amino acids or GLU) to the organism for longer periods, thus ameliorating diabetic phenotypes. We initially found that InR or Pdpk1 KD suppressed proteasome activities, increased the mitochondrial network density and mildly enhanced lipolysis (Supporting information Figure S13). We then established transgenic flies, where CncC/Nrf2 OE was combined with InR or Pdpk1 KD and found that both genetic interventions largely rescued the CncC/Nrf2 OE‐mediated larval growth retardation (Figure 5a) and mitigated CncC/Nrf2 OE‐induced hyperglycemia in adult flies (Figure 5b). Also, either genetic (InR or Pdpk1 KD) (Figure 5c) or dietary (CR) (Figure 5d) constant early IIS downregulation extended the longevity of CncC/Nrf2 overexpressing flies.

Similarly, early constant Atg8α OE largely rescued the CncC/Nrf2 OE‐mediated larval growth retardation, alleviated lipolysis in larval fat body after targeted transgenes expression in muscle, and suppressed CncC/Nrf2 OE‐mediated hyperglycemia (Supporting information Figure S14A). Also, Atg8α OE increased the longevity of CncC/Nrf2 overexpressing flies (Supporting information Figure S14B). We then established transgenic flies where CncC/Nrf2 OE was combined with Gys KD (Supporting information Figure S11A). We found that Gys KD reduced the intensity of CncC/Nrf2 overactivation‐mediated proteome over‐ubiquitination, Ref(2)P upregulation (Supporting information Figure S14C1) and proteasome activation (Supporting information Figure S14C2) (compare with Supporting information Figure S3C2). Furthermore, while Gys KD did not affect the mitochondrial respiratory control ST3/ST4 and FCCP/ST4 ratios, it decreased the absolute ST2, ST3, ST4, and FCCP values; tended to suppress maximum mitochondrial respiration (FCCP values) (compare with Figure 2) and largely normalized the expression of mitochondrial genes (Supporting information Figures S14C3–S14C5) in CncC/Nrf2 OE flies. It also partially alleviated lipolysis in larval fat body after targeted transgenes expression in muscle (Supporting information Figure S14D), normalized the GLU/TREH content in CncC/Nrf2 overexpressing flies’ tissues (Supporting information Figure S14E) indicating a more physiological IIS; enhanced the survival of CncC/Nrf2‐overexpressing flies during increased periods of flight (Supporting information Figure S14F1), and it delayed aging under normal culture conditions (Supporting information Figure S14F2). NMR‐based metabolomics analyses largely confirmed these findings as we (among others) found that Gys KD in CncC/Nrf2 OE flies suppressed TREH accumulation and alleviated (or inverted) alanine, proline and lactate downregulation (Supporting information Figure S15, Supporting information Table S7). Thus, IIS downregulation delays the exhaustion of energetic biomolecules in CncC/Nrf2 overexpressing flies and suppresses diabetes‐like phenotypes.

**Figure 6:**
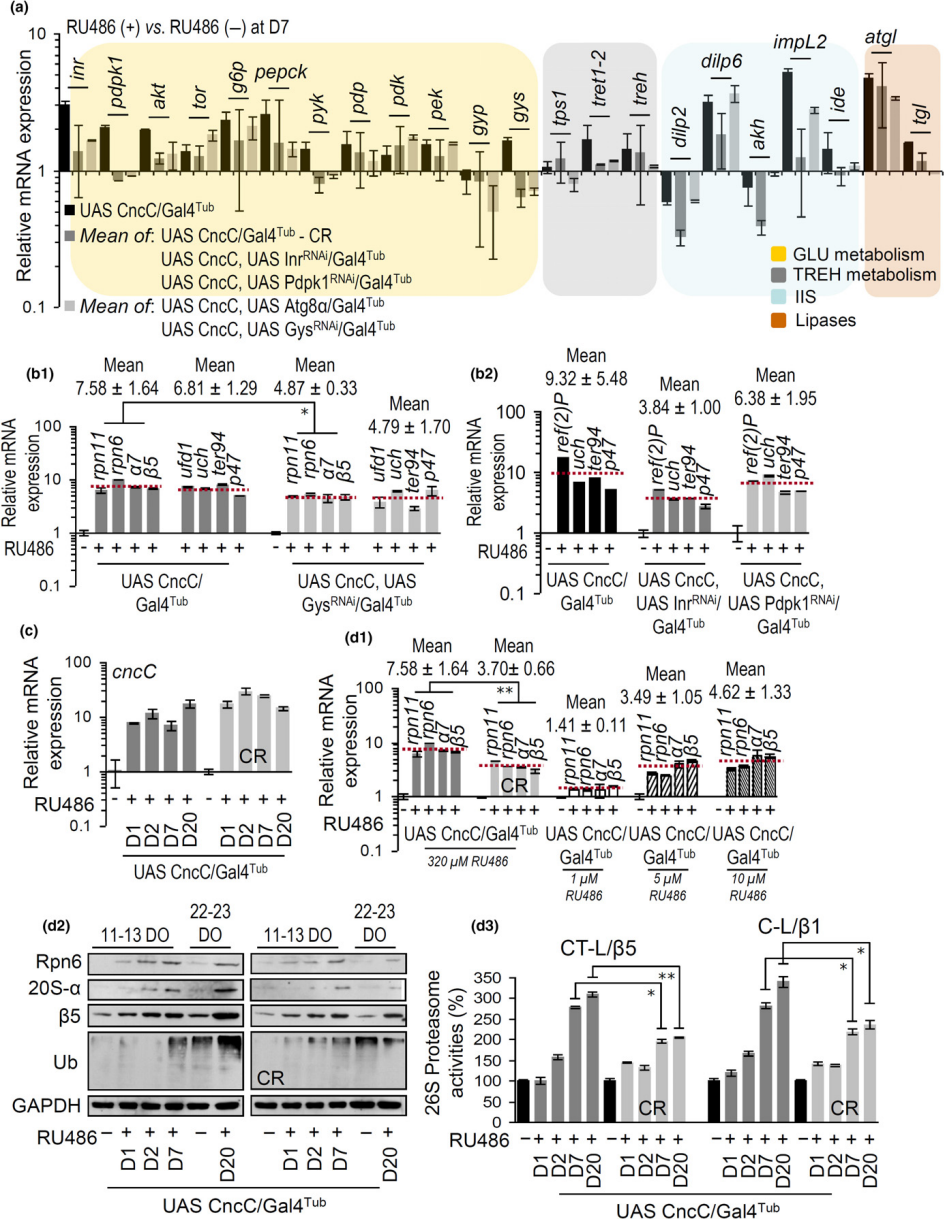
IIS downregulation mitigates the transcriptional output of CncC/Nrf2 despite sustained OE of the transgene. (a) Relative expression of the shown genes at the indicated transgenic fly lines; values refer to mean expression per gene (vs. their respective control) of the indicated genotypes (see also Supporting information Figure S16). (b_1_, b_2_) Relative expression of rpn11, rpn6, α7, β5, ref(2)P, uch, ter94, and p47 genes at the shown transgenic lines; mean expression values/genotype/group of genes is also indicated. (c) Relative expression levels of the cncC/Nrf2 transgene (at days indicated) in transgenic flies fed with normal culture medium (left bars) or after CR. (d_1_) Relative expression of rpn11, rpn6, α7, and β5 genes in flies fed with normal medium or after CR; gene expression values in flies treated with 1, 5, or 10 μM of RU486 and mean expression values/genotype/group of genes are also shown. (d_2_, d_3_) Immunoblot analyses (d_2_) or relative (%) 26S proteasome activities (d_3_) (at days indicated) in CncC/Nrf2 overexpressing (or not) flies’ tissues fed with normal medium or after CR. Samples in (d_2_) were probed with antibodies against proteasome subunits Rpn6, 20S‐α, and β5 or ubiquitin. Unless otherwise indicated samples were collected from young flies being exposed to 320 μM RU486 for 7 days. Gene expression was plotted vs. the respective control set to 1. GAPDH probing or rp49 gene expression were used as reference. Bars, ±*SD*; *p < 0.05; **p < 0.01

Mechanistically, as deduced from Supporting information Figure S11A, early constant IIS suppression would result in reduced Nrf2 activity, for example, due to its inhibition by upstream activated Gsk3. We thus compared mean metabolic and proteostatic genes’ expression levels in CncC/Nrf2 overexpressing flies vs. the other transgenic lines used or CR treatment. As summarized in Figure 6a (see also Supporting information Figure S16), our genetic or dietary interventions caused milder deregulation of metabolic genes’ expression. A similar effect of less intense transcriptional output was also evident after comparing the expression of proteostatic genes (which represent direct transcriptional targets of CncC/Nrf2) in double transgenic lines vs. CncC/Nrf2 overexpressing flies (Figure 6b). The impact of reduced IIS on CncC/Nrf2 activity was even more dramatic after CR, since although CR did not affect the levels of inducible transgene expression (Figure 6c), it significantly suppressed the upregulation of direct CncC/Nrf2 targets, namely proteasomal genes (Figure 6d_1_), protein subunits (Figure 6d_2_), and peptidases activities (Figure 6d_3_). The reduced CncC/Nrf2 activity upon IIS suppression was also confirmed in flies where CncC/Nrf2 OE was combined with dIlp2 KD (Supporting information Figure S17A,B, S17B). These flies also displayed, as compared to CncC/Nrf2 overexpressing flies, normalized mitochondrial function (Supporting information Figure S17C), similar resistance to oxidants (Supporting information Figure S17D), and increased healthspan/lifespan (Supporting information Figure S17E) (Supporting information Table S1). Therefore, IIS suppression titrates CncC/Nrf2 activity to lower levels, reducing thus the emitted stress signaling.

## 3 DISCUSSION

We report here that in spite of activating a wide panel of cytoprotective modules in a tunable manner, only mild CncC/Nrf2 activation enhanced healthspan, and in fact only marginally. In contrast, higher activation levels either caused larval lethality or significantly reduced adult longevity. Supportively, whereas loss of one *keap1 *copy, heterozygosity for the *keap1* ^EY5 ^loss‐of‐function mutation, or pharmacological inhibition of Gsk3, activated CncC/Nrf2 and increased stress resistance and flies’ longevity (Castillo‐Quan et al., 2016; Sykiotis & Bohmann, 2008; Tsakiri et al., 2017), deletion of *keap1 *led to lethality during mid‐larval development (Sykiotis & Bohmann, 2008). In *C. elegans *transgenic SKN‐1 OE in the intestine promoted longevity, whereas SKN‐1 expression from high‐copy transgenic arrays was toxic (Tullet et al., 2008). Similarly, strong activation of UPR^ER ^in *C. elegans *conferred stress resistance while shortening lifespan (Taylor & Dillin, 2013) and Nrf2 overactivation in mouse keratinocytes resulted in epidermal inflammation (Schäfer et al., 2012); also, persistent Nrf2 activation in rodents caused lifespan alteration and stem cell exhaustion (Murakami et al., 2017). Thus, the activation level of Nrf2 that enhances healthspan/lifespan is considerably lower than that which maximizes protection against toxic doses of stressors.

Our data suggest that Nrf2 also regulates mitochondrial dynamics and energetics. Consistently, recent reports indicated that Nrf2 upregulates electron transport‐related genes (Misra, Horner, Lam, & Thummel, 2011) and affects mitochondria biogenesis (Palikaras, Lionaki, & Tavernarakis, 2015). Our findings extend these observations by demonstrating that CncC/Nrf2 regulates mitochondrial dynamics, energetics, O_2_ consumption, and ATP production, assembly of OXPHOS machineries and protein supercomplexes; it also mediates upregulation of mitochondrial genes after proteotoxic stress. Thus, Nrf2 is likely a key immediate sensor of altered mitochondrial energetics and/or ROS production, consistent with its tethering to the cytoplasmic side of the outer mitochondrial membrane with the mitochondrial protein PGAM5 *via *Keap1 binding (Sykiotis & Bohmann, 2010).

Sustained CncC/Nrf2 activation in flies’ tissues promoted late downregulation of IIS, as part of a Nrf2‐driven circuit that aims to suppress its own activity (Supporting information Figure S11A), leading to hyperglycemia, extensive lipolysis, and DT1‐like phenotypes. Previous studies have shown that activation of Nrf2 in the liver reduced lipid levels (Chambel, Santos‐Gonçalves, & Duarte, 2015) and that Nrf2 regulated enzymes involved in GLU metabolism (Lacher et al., 2015); thus, Nrf2 likely modulates a wide panel of metabolic genes. Similarly to our findings, ablation of IPCs in flies caused developmental delay, growth retardation, and hyperglycemia (Rulifson, Kim, & Nusse, 2002), while deletion of dIlps1–5 generated small homozygotes with elevated circulating sugar levels, decreased triglycerides, and activated autophagy; these animals were growth‐delayed and poorly viable (Zhang et al., 2009). IIS signaling in flies is also regulated by the secreted protein ImpL2 that binds and inhibits dIlps 2 and 5 (Alic, Hoddinott, Vinti, & Partridge, 2011). Consistently to our observations, inhibition of IIS in flies by ImpL2 OE caused systemic cachexia‐like organ wasting (Kwon et al., 2015). Low IIS results in reduced GLU uptake from muscle; and hence, lactate and alanine (which decrease in CncC/Nrf2 overexpressing flies) are exported to the fat body for conversion into GLU (Berg, Tymoczko, & Lubert Stryer, 2002). After exhaustion of the lipid reservoirs, the only source of GLU precursors is proteolysis‐derived amino acids triggering muscle wasting. This adverse effect is evident in DT1 patients, who in the absence of insulin replacement are in a catabolic state that results in severe depletion of both energy stores and protein mass (denutrition and cachexia); eventually, untreated DT1 patients develop severe neuropathy, myopathy, and/or cardiomyopathy due to muscle wasting (D'Souza, Al‐Sajee, & Hawke, 2013). As CncC/Nrf2 overexpressing flies recapitulate these effects they provide a useful model to identify new mechanisms and therapeutic targets for these severe complications.

Prolonged stress signaling seems to be centrally linked to IIS downregulation, since genotoxic stress in XPF‐ERCC1‐deficient mice suppresses the somatotroph axis triggering somatic growth attenuation (Niedernhofer et al., 2006), and impaired genome maintenance suppresses the growth hormone/IGF‐1 axis in mice with Cockayne syndrome (Van der Pluijm et al., 2007). Also, DNA damage in *Drosophila *larva epidermis induces an innate immune response that is kept in control by repression of IIS activity (Karpac, Younger, & Jasper, 2011). The same mechanism likely operates under other types of stress, since, for example, proteotoxic stress due to proteasome dysfunction mediates insulin resistance in the liver (Otoda et al., 2013). Also, flies infected with *Mycobacterium marinum *progressively lose metabolic stores of fat and become hyperglycemic (Dionne, Pham, Shirasu‐Hiza, & Schneider, 2006), while in support of our proteomics findings of reduced abundance of yolk proteins after CncC/Nrf2 OE, *inr *mutant females are non‐vitellogenic (Giannakou & Partridge, 2007). Thus, given also our observations in proteomics analyses showing that sustained CncC/Nrf2 activation suppressed the expression of proteins involved in courtship behavior, mating and reproduction, sleep and circadian rhythms, it emerges that persistent stress affects most regulatory networks of metazoans.

Interestingly, muscle‐targeted CncC/Nrf2 OE promoted systemic effects in the fat body lipid content, indicating that normal Nrf2 activity in muscles is required non‐autonomously to maintain physiological fat body functionality. Similarly, Foxo activation in the adult pericerebral fat body reduces expression of the dIlp2 synthesized in neurons and represses IIS in peripheral fat body (Hwangbo, Gershman, Tu, Palmer, & Tatar, 2004). Also, ImpL2 secretion from muscles with mitochondrial distress triggers non‐autonomous repression of IIS (Owusu‐Ansah, Song, & Perrimon, 2013) and an aggregation‐prone protein expressed in the neurons of *C. elegans *elicits a mitochondrial‐specific unfolded protein response that affects whole‐animal physiology (Berendzen et al., 2016). Our findings are thus consistent with the existence of dynamic communication between stress pathways in muscle and adaptive programs in other peripheral organs that are activated through central integration of signals spanning multiple tissues.

Since Nrf2 overactivation is likely a primary output of increased IIS in metabolic disorders (e.g., obesity), our observation that CncC/Nrf2 OE‐induced diabetic phenotypes can be mitigated by early constant lowering of IIS or by modulating IIS downregulation end points (e.g., ALP activation or Gys inhibition) is of particular importance. Supportively, autophagy activation had a renoprotective role in diabetic nephropathy (Xu et al., 2015) and it improved ER stress‐induced diabetes in a rodent model (Bachar‐Wikstrom, Wikstrom, Kaiser, Cerasi, & Leibowitz, 2013). Also, a genetic mutation of the Gys inhibiting enzyme Gsk3β (that renders Gsk3β resistant to IIS inhibition) corrected diabetes in mouse models of insulin resistance (Tanabe et al., 2008) and protected against metabolic syndrome (Chen et al., 2016). Moreover, Gys KD in *Drosophila *neurons improved neurological function and extended lifespan (Sinadinos et al., 2014). Likely a cross‐talk exists between autophagy and GLY breakdown in *Drosophila*, since it was found that autophagy is required for efficient degradation of GLY in response to starvation (Zirin, Nieuwenhuis, & Perrimon, 2013). Therefore, Atg8α activation may also alleviate the effects of CncC/Nrf2 OE by enhancing GLY breakdown (mimicking Gys KD) and thereby increasing GLU availability. A consistent and intense rescue of the CncC/Nrf2 OE‐induced toxic effects in adult flies was observed after genetic or CR‐mediated constant early IIS downregulation. In support, it was showed that restricted diet delays accelerated aging, improves neuronal function and alleviates genomic stress in DNA repair‐deficient mice (Vermeij et al., 2016). Our finding that IIS downregulation titrates CncC/Nrf2 activity to lower levels is supported by our observation that the CncC/Nrf2 OE mean transcriptional output on proteostatic genes of CR‐treated flies exposed to 320 μM RU486, was similar to that found in flies cultured in normal medium containing 5 μM RU486 (Figure 6d_1_). Consistently, the median longevity of CR‐treated CncC/Nrf2 overexpressing flies exposed to 320 μM RU486 was 35 days, which roughly corresponds to the median longevity of flies reared in normal culture medium containing 1–5 μM RU486 (Supporting information Table S1). Thus, IIS downregulation is dominant over CncC/Nrf2 (and likely of other stress sensors) overactivation; this finding suggests therapeutic dietary interventions for various age‐related diseases of chronic stress including progeroid genome instability syndromes and/or neurodegeneration.

Taken together, our findings suggest that even in the absence of biomolecular damage, persistent stress signaling triggers a highly conserved adaptive metabolic response which reallocates resources from growth and longevity to somatic preservation and stress tolerance (Supporting information Figure S18). This notion provides a reasonable explanation of why most (if not all) cytoprotective stress sensors (e.g., Nrf2, Foxo, p53, etc) are short‐lived proteins, and it also explains the build‐in negative feedback loops (shown here for Nrf2); the low basal levels of these proteins, and why their suppressors were favored by evolution. Despite the severe adverse effects of Nrf2 overactivation, none is sufficient reason to discredit the Nrf2 pathway as a drug target, for example, for anti‐aging purposes. Evidence comes from the fact that humans have been safely ingesting Nrf2 activators in their diet for millennia and from the increased healthspan associated with mild Nrf2 activation. Yet, the critical issues of correct dosage of stress sensors activators and of their interactions with disease‐related pathways remain critical to avoid clinical trial failures. Systematic analyses of the cross‐talk and functional interactions of pathways controlling stress and metabolic responses in model organisms can provide valuable preclinical insights and elucidate potential therapeutic avenues against aging and age‐associated pathologies.

## 4 EXPERIMENTAL PROCEDURES

### 4.1 Fly stocks (maintenance and transgenic lines)

The transgenic lines, UAS CncC, UAS CncC^Δ1‐87^, UAS CncC^RNAi^, UAS Keap1, and UAS Keap1^RNAi^; the gstD‐ARE:GFP/II (ARE of the *gstd *gene) and the gstD‐mARE:GFP/III (mutated version of gstD‐ ARE) reporter transgenic lines, as well as the Tubulin GeneSwitch Gal4 (tubGSGal4) driver, were a gift from Prof. D. Bohmann (University of Rochester, NY, USA); the conditional driver (tubGSGal4) is ubiquitously activated upon dietary administration of RU486. The w^1118^ stock and the transgenic strains UAS Atg8α.GFP, UAS Gys^RNAi^, UAS Pdpk1^RNAi^, UAS Inr^RNAi^, and UAS dIlp2^RNAi ^along with another Keap1^RNAi^ line were obtained from the Bloomington *Drosophila *Stock Center. To establish isogenic lines, the UAS CncC and the conditional driver tubGSGal4 transgenes were backcrossed ten times into the w^1118^ genetic background. The reporter lines UAS mCherry‐Atg8α, GFP‐Lamp1, and UAS Mito^GFP^, along with the ubiquitous Gal4^Actin^; the nervous system‐specific Gal4^Elav^ and Gal4^D42^, and the muscle‐specific Gal4^Mef2^ and Gal4^MHC^ drivers were a gift from Prof. A. Daga (University of Padua, Padova, Italy). Since gonads display distinct aging rates and regulation of proteostatic mechanisms as compared to adult somatic tissues (Tsakiri, Sykiotis, Papassideri, Gorgoulis, et al., 2013), in all presented experiments referring to adult flies only microdissected somatic tissues (head and thorax; equal numbers from mated male and female flies) were analyzed.

### 4.2 Flies culture, exposure to compounds, starvation and increased flight periods

Flies were maintained at 23°C, 60% relative humidity on a 12‐hr light: 12‐hr dark cycle and were fed standard medium (Trougakos & Margaritis, 1998). All used compounds [chloroquine (CQ; Sigma), the proteasome inhibitor PS‐341 (Calbiochem) and RU486 (Sigma)] were added in culture medium; doses and duration of flies’ exposure to compounds are indicated in figure legends.

The CR assay was performed in young mated flies fed with standard medium containing 0.4% yeast extract. For starvation experiments, flies were exposed to 1.5% agar medium; experiments of increased flight periods were performed by placing a culture vial into an empty bigger vial (Figure 3e_1_).

For survival curves and statistical analyses, the Kaplan–Meier procedure and log‐Rank (Mantel‐Cox) test were used; significance was accepted at *p* < 0.05. Statistical analyses for all longevity experiments are reported in Supporting information Table S1.

Full Methods are available in Supporting information Appendix S1.

## CONFLICT OF INTEREST

The authors declare no conflict of interest.

## AUTHOR CONTRIBUTIONS

IPT designed and supervised the study; ENT, SG, KKI and IPT conducted experiments or interpreted the data; DB and EM performed the metabolomics analysis; KV conducted the proteomics experiments; GPS, VGG, and LS generated or contributed reagents, materials, analysis tools and edited the manuscript; IPT wrote the manuscript.
