# Supplementary material for: Hyperactivation of Nrf2 increases stress tolerance at the cost of aging acceleration due to metabolic deregulation
Source: Aging Cell. 2018 Dec 10;18(1):e12845. doi: 10.1111/acel.12845 (PMC6351879; doi:10.1111/acel.12845)
Supplement: Supplementary file 1 [file ACEL-18-e12845-s001.pdf]

Hyperactivation of Nrf2 increases stress tolerance at the cost of aging acceleration due to metabolic deregulation

Eleni N. Tsakiri, Sentiljana Gumeni, Kalliopi K. Iliaki, Dimitra Benaki, Konstantinos Vougas, Gerasimos P. Sykiotis, Vassilis G. Gorgoulis, Emmanuel Mikros, Luca Scorrano and Ioannis P. Trougakos

Supporting Information

Supporting Figure Legends

Figure S1. Treatment of flies with RU486 neither activates antioxidant response elements or proteostatic genes nor does it affect metabolism or flies' longevity. (A) Relative (%) green fluorescent protein (GFP) fluorescent levels (A_1) and immunoblotting analysis of GFP expression (A_2; blots were probed with an anti-GFP antibody) following exposure (or not) of young transgenic gstD-ARE:GFP or gstD-mARE:GFP flies to RU486 for 3 days. (B) Relative (%) 26S proteasome activities in RU486 treated control flies at day 1 (D1) and day 7 (D7). (C) Relative expression of *β5*, *β1*, *cncC*, *keap1* and *trxr-1* genes in +/Gal4^{Tub} flies exposed (or not) to RU486 for 7 days. (D) Relative GLU and TREH content in flies' tissues exposed (or not) to RU486 for 7 days. (E) Longevity curves of +/Gal4^{Tub} flies treated (or not) with RU486. In all shown experiments, flies were treated with 320 μM RU486. Gene expression was plotted *vs*. the respective control set to 1. GAPDH probing (A_2) and *rp49* gene expression (C) were used as input reference. Statistics of the longevity curves are reported in Table S1. Bars, ± SD.

Figure S2. The *cncC/nrf2*, *maf-S* and *keap1* genes are ubiquitously expressed [albeit in lower levels as compared to the proteostatic *β5*, *atg8α* and *ref(2)P* genes] in flies' tissues. Q-PCR quantification cycle (Cq) values for the *rp49* (housekeeping), *cncC*, *maf-S*, *keap1*, *β5*, *atg8a* and *ref(2)P* genes in whole flies preparations or the indicated dissected male or female tissues. The mean Cq values/per gene for the various tissues analyzed are also indicated. Bars, ± SD.

Figure S3. CncC/Nrf2 OE activates cytoprotective proteostatic modules in a dose-dependent manner. (A_1) Schematic representation of full length CncC/Nrf2 (adapted from Pitoniak and Bohmann, 2015); the NHB1 subdomain (NTD domain) that relates to ER localization, the DNA binding region (bZIP, NEH1 domain), the Keap1 interaction motifs (DGL/ETGE, NEH2 domain), the Sgg/Gsk3 phosphorylated serine residues (S, NEH6 domain) and the start site of the truncated CncC/Nrf2$^{\Delta 1\text{-}87}$ are indicated. (A_2) Immunoblots of shown protein samples probed with an anti-FLAG antibody after immunoprecipitation of FLAG-tagged CncC/Nrf2. (B) Immunoblot analyses of tissues protein samples probed with antibodies against FLAG (binds to CncC/Nrf2 transgene), proteasomal subunits Rpn10, 20S-α, β5 and total protein ubiquitination (Ub; arrow denotes free ubiquitin) after ubiquitous CncC/Nrf2 OE. (C_1) Immunoblots after immunoprecipitation of tissue lysates with a 20S-α antibody and probing with antibodies against β5 and 20S-α. (C_2) Relative (%) 26S proteasome activities in control or CncC/Nrf2 overexpressing flies. (C_3) *In-gel* non-denatured native proteasome activity viewed by UV (upper panel; the various proteasomal forms, i.e. 30S, 26S and 20S are indicated) or after immunoblotting and probing with anti-20S-α and Rpn10 antibodies (lower panels). (D) Relative expression of *rpn11*, *rpn6*, *α7*, *β5*, *gstd1*, *trxr-1, keap1* and *maf-S* genes in control samples or after ubiquitous CncC/Nrf2 OE. (E) Relative (%) ROS levels in flies' tissues after CncC/Nrf2 or Keap1 OE, as well as after CncC/Nrf2 KD. (F) Immunoblot analyses of total protein carbonylation (DNP) in flies' tissues after CncC/Nrf2 OE or CncC/Nrf2 KD. In (A-D) data refer to isogenic transgenic flies. If not otherwise indicated, young mated flies were exposed to 320 μM RU486 for 7 days. Gene expression was plotted *vs*. the respective control set to 1; in (C_2, E) control values were set to 100%. GAPDH probing (A_2-C, F) and *rp49* gene expression (D) were used as input reference. Bars, ± SD; *$P < 0.05$; **$P < 0.01$.

Figure S4. RNAi mediated *keap1* KD activates cytoprotective antioxidant responses and proteostatic modules in a dose-dependent manner; yet, prolonged *keap1* KD results in significant

reduction of flies' longevity. (A) Immunoblotting analysis of GFP expression denoting AREs activation in control flies or after muscle targeted *keap1* KD. (B) Relative expression of *rpn11*, *α7*, *β5*, *gstd1* and *trxr-1* genes in control samples or after ubiquitous *keap1* KD; flies were treated with 10, 30 or 60 μM RU486. (C, D) Representative immunoblotting analyses of tissues protein samples probed with antibodies against proteasomal subunits Rpn6, 20S-α, β5 and total protein ubiquitination (Ub) (C); and relative (%) 26S proteasome peptidases activities (D) in control flies or after *keap1* KD. (E) Longevity curves of transgenic flies after inducible *keap1* KD. If not otherwise stated flies were exposed to 320 μM RU486; in all shown experiments the duration of young flies' exposure to RU486 was 7 days. Gene expression was plotted *vs*. the respective control set to 1; in (D) control values were set to 100%. GAPDH probing (C) and *rp49* gene expression (B) were used as reference. Bars, ± SD; $^*P < 0.05$; $^{**}P < 0.01$.

Figure S5. OE of a truncated CncC/Nrf2$^{\Delta 1\text{-}87}$ form activates cytoprotective antioxidant responses and proteostatic modules in a dose-dependent manner; yet, its sustained OE in adult flies accelerates aging. (A) Relative expression of *α7*, *β5* and *trxr-1* genes in control samples or after ubiquitous inducible CncC/Nrf2$^{\Delta 1\text{-}87}$ OE with the shown concentrations of RU486. (B_1) Representative immunoblotting analyses of tissues protein samples, after CncC/Nrf2$^{\Delta 1\text{-}87}$ OE (or not), probed with antibodies against proteasomal subunits Rpn7, 20S-α, β5 and total protein ubiquitination (Ub). (B_2) Relative (%) 26S proteasome peptidase activities in control or CncC/Nrf2$^{\Delta 1\text{-}87}$ overexpressing flies. (C) Immunoblot analyses after exposing flies to the shown concentrations of Chloroquine (CQ) or PS-341, and/or CncC/Nrf2$^{\Delta 1\text{-}87}$ induction by RU486; samples were probed with an anti-β5 antibody. (D) Relative (%) ROS levels in flies' tissues after CncC/Nrf2$^{\Delta 1\text{-}87}$ OE. (E) Longevity curves of control (+/Gal4^{Tub}) or CncC/Nrf2$^{\Delta 1\text{-}87}$ overexpressing transgenic flies cultured in the absence of RU846 (E_1) or with the indicated RU486 concentrations (E_2). Data refer to isogenic transgenic flies. If not otherwise indicated young mated flies were exposed to 320 μM RU486 for 7 days. Gene expression was plotted *vs*. the respective control set to 1; in (B_2, D) control values were set to 100%. GAPDH (B_1) or Tubulin (C) probing and *rp49* gene expression (A) were used as reference. Bars, ± SD; $^*P < 0.05$; $^{**}P < 0.01$.

Figure S6. Gene expression and proteomic analyses by nano-LC-ESI-MS/MS of CncC/Nrf2 overexpressing flies' somatic tissues indicate that CncC/Nrf2 is a main regulator of proteostatic modules. (A_1) Relative expression of *atg8α*, *ref(2)P*, *hdac6*, *hsp70* and *grp78* genes in adult flies overexpressing CncC/Nrf2. (A_2) Immunoblots after probing samples from CncC/Nrf2 overexpressing adult flies with antibodies against Atg8α and Ref(2)P. (A_3, B) Immunoblots after probing samples from CncC/Nrf2 overexpressing larvae with antibodies against Ref(2)P (A_3) or total protein ubiquitination (Ub) (B). (C) Graphical representation of the high resolution iTRAQ proteomics data; arrows denote selected proteostatic modules that were also studied at the gene expression level. Proteins identified by iTRAQ proteomics to be differentially expressed (*vs*. control flies) after inducible CncC/Nrf2 OE are reported in Table S2 (for their human orthologs, see Table S3). (D, E) Relative expression of the *ufd1*, *uch*, *ter94*, *p47* and *aos1* genes at the indicated transgenic adult flies (D) or larvae (E) tissues. For studies shown in (A), as well as for iTRAQ proteomics (C) young flies were exposed to RU486 for 7 days; in other cases (D_1, D_2) the duration of the RU486 treatment (in days) was as indicated (D1, D7 or D20). Gene expression in (A_1), (D), (E) was plotted *vs*. the respective control set to 1. In (D_1), (D_2) only the control values of *ufd1* and *ter94* genes respectively are shown. ND, not determined; GAPDH probing (A_2-B) and *rp49* gene expression (A_1, D, E) were used as reference. Bars, ± SD; $^*P < 0.05$; $^{**}P < 0.01$.

Figure S7. ChIP genomic analyses reveal CncC/Nrf2 binding to regulatory regions of proteostatic genes. Differential enrichment of CncC/Nrf2 binding to regulatory regions of the shown genes, following CncC/Nrf2 inducible OE with 320 μM RU486 for 7 days [UAS CncC/Gal4^{Tub} RU486 (+) *vs*. UAS CncC/Gal4^{Tub} RU486(-) flies]. ChIP assays were performed with a CncC/Nrf2 transgene specific anti-FLAG antibody. Bars, ± SD; $^*P < 0.05$; $^{**}P < 0.01$.

Figure S8. Prolonged CncC/Nrf2$^{\Delta 1\text{-}87}$ activation results in Diabetes-like phenotypes. (A) CLSM visualization of fat bodies after BODIPY staining of transgenic CncC/Nrf2$^{\Delta 1\text{-}87}$ overexpressing (or not) flies; samples were also stained by immunofluorescence for α-GLY and counterstained with DAPI. (B) CLSM visualization following immunofluorescence staining of adult CncC/Nrf2$^{\Delta 1\text{-}87}$

overexpressing flies' muscle tissues with an α-GLY antibody; samples were counterstained with Phalloidin and DAPI. Flies were exposed to RU486 for 1 or 7 days.

Figure S9. NMR-based metabolomics analysis in flies' somatic tissues after CncC/Nrf2 OE or KD. Results (depicted in box plots) are shown *vs*. corresponding controls of non-induced transgene. Dashed line denotes the mean value found in CncC/Nrf2 overexpressing flies and can be directly compared to the value noted after inducible CncC/Nrf2 KD. For metabolomics studies, young mated flies were exposed to RU486 for 7 days. A detailed description of the differences in metabolite concentrations observed at the NMR spectra (as depicted by z-score transformation) is reported in Table S6. For statistical analyses of the obtained data see Supporting Experimental Procedures.

Figure S10. Comparative short- and long-term genomic responses of metabolic genes after CncC/Nrf2 activation (by CncC/Nrf2 OE or Keap1 KD) or following CncC/Nrf2 KD. (A) Relative expression of shown genes (coloring denotes different metabolic pathways) at the indicated transgenic lines. The genotype per analyzed sample is indicated in each panel. Samples were collected from a single starting mixed population of young mated flies being exposed to 320 μM of RU486 for 1, 2 or 7 days (young), as well as after exposure to RU486 for 20 days (middle aged). Gene expression was plotted *vs*. the respective control set to 1; in all cases, only the control value of the *inr* gene is shown. (B) Relative expression of *sgg* gene in flies' tissues after inducible CncC/Nrf2 OE for 7 days. ND, not determined; *rp49* gene expression was used as reference. Bars, ± SD; *P < 0.05; **P < 0.01.

Figure S11. Prolonged CncC/Nrf2 OE results in autophagic activity. (A) Schematic presentation of the IIS pathway activation that culminates in Sgg/Gsk3 suppression; additional modules (e.g. Foxo, Nrf2 and Gys) and their reported regulatory inter-relationships (→ denotes positive regulation and ┤a negative regulatory effect) are also shown. Colored rectangles (blue KD, red OE) denote pathways that were modulated in rescue experiments (i.e. early IIS or Gys KD and ALP activation). (B) Immunoblot analyses of CncC/Nrf2 overexpressing (or not) somatic tissues protein samples probed with antibodies against phospho-Akt, Foxo, phospho-AMPKα and AMPKα (B_1) or, with antibodies against phospho-Gsk3$^{S21/S9}$ (inhibitory) and Gsk3 (B_2). (C) Relative expression of the *atg6* gene at the somatic tissues of the indicated transgenic lines. (D) CLSM visualization of the mCherry-Atg8α reporter in the nervous tissues of control or CncC/Nrf2 overexpressing transgenic larvae. (E) Relative (%) cathepsin B, L activities in flies' tissues of the indicated genotypes. (F) CLSM visualization of the GFP-Lamp1 reporter in nervous tissues of the shown transgenic lines. In the inducible Gal4^{Tub} ubiquitous driver, flies were exposed to 320 μM RU486 for 7 days. Gene expression was plotted *vs*. controls set to 1; in (E) control values were set to 100%. GAPDH (B_1) or Tubulin (B_2) probing and *rp49* gene expression (C) were used as reference. Bars, ± SD; *P < 0.05.

Figure S12. Targeted CncC/Nrf2 OE at high doses in muscle is lethal at early developmental stages, deregulates metabolic pathways and promotes systemic effects in the fat body; at low doses, it mildly decreases fat body lipids and in the adult exhausts the capability of flight muscles to contract. (A) Relative expression of proteasomal *β1*, *β2*, *β5*, *α5*, *α7*, *rpn6*, *rpn10* and *rpn11* genes, as well as of *ref(2)P*, *gstd1*, *trxr-1*, *inr*, *gys*, *pepck* and *g6p* genes in control larvae samples or after muscle-targeted (Gal4^{Mef2} driver) CncC/Nrf2 OE. (B) Relative (%) 26S proteasome or cathepsin B, L activities in control larvae or after CncC/Nrf2 OE in muscles. (C) Stereoscope viewing of 3rd instar larvae (left panel) and pupae (right panel) of the indicated genotypes. (D) CLSM visualization of CncC/Nrf2 overexpressing (or not) larvae muscles tissues following immunofluorescence staining with an α-GLY antibody; samples were counterstained with Phalloidin and DAPI. (E) CLSM visualization (E_1) and quantitative analysis (E_2) of mean lipid particle size (LPS), corrected total fluorescence (CTF) and lipid particle number/μm^2 after BODIPY staining of larvae fat bodies; samples were also stained by immunofluorescence for α-GLY and counterstained with DAPI. (F_1) Stereoscope viewing of 3rd instar control or transgenic larvae after CncC/Nrf2 OE in muscles with the Gal4^{MHC} driver. (F_2) CLSM visualization after BODIPY staining of transgenic CncC/Nrf2 muscle-overexpressing larvae fat bodies; samples were also stained by immunofluorescence for α-GLY and counterstained with DAPI. (G) Stereoscope viewing of wings position in middle aged flies of the shown genotypes. Gene expression was plotted *vs*. respective controls set to 1. In (B), (E_2; left panel) control values were set to 100%, while in (E_2; right panel) the absolute values are reported. Bars, ± SD; *P < 0.05; **P < 0.01.

Figure S13. Effects of Inr or Pdpk1 KD on proteasome activities, density of mitochondrial network and fat body lipids. (A) Relative (%) proteasome CT-L/β5 activities in flies tissues expressing the shown transgenes. (B) CLSM visualization of $Mito^{GFP}$ reporter in larval muscle tissues following Inr or Pdpk1 KD. (C) CLSM visualization of fat bodies after BODIPY staining of transgenic larvae expressing (or not) Inr or Pdpk1 RNAi. In (A) control values were set to 100%. Bars, ± SD; *P < 0.05.

Figure S14. Enhancing the expression of autophagic effectors (Atg8α) or suppressing Gys partially rescues the CncC/Nrf2 OE-induced effects on metabolic pathways, larvae growth and adult flies' longevity. (A_1) Stereoscope viewing of 3rd instar control (+/$Gal4^{Mef2}$) or transgenic larvae expressing the indicated transgenes. (A_2) CLSM visualization of fat bodies after BODIPY staining of transgenic larvae overexpressing (or not) CncC/Nrf2 and Atg8α. (A_3) Relative (%) content (*vs*. controls) of GLU, GLY and TREH in the indicated transgenic line somatic tissues. (B) Longevity curves of the indicated transgenic lines (the UAS CncC/$Gal4^{Tub}$ curve is also shown in Fig. 5C). (C) Immunoblots of protein samples probed with antibodies against total protein ubiquitination (Ub) and Ref(2)P (C_1); and relative (%) 26S proteasome activities (C_2) in tissue samples after inducible CncC/Nrf2 OE and Gys KD. (C_3-C_5) Relative mitochondrial ST2, ST3/ST4, FCCP/ST4 and ADP/ST3 ratios (C_3); absolute values of ST2, ST3, ST4 and FCCP (C_4), and relative expression of *marf*, *drp1*, *opa1* and *sdhA* (C_5) genes in the indicated transgenic flies. (D) CLSM visualization (D_1) and quantitative analysis (D_2) of mean lipid particle size (LPS), corrected total fluorescence (CFT) and lipid particle number/μm^2 after BODIPY staining of larval fat bodies of the shown genotypes. (E) Relative (%) content (*vs*. controls) of GLU, GLY and TREH after Gys KD in CncC/Nrf2 overexpressing flies. (F_1) Longevity curves of the indicated transgenic lines after facilitating increased flying periods (the UAS CncC/$Gal4^{Tub}$ curve is also shown in Fig. 3E_2). (F_2) Longevity curves of the indicated transgenic lines after RU486-mediated induction of the transgenes. Statistics of the longevity curves are reported in Table S1. Unless otherwise indicated flies were exposed to 320 μM RU486 for 7 days. Gene expression was plotted *vs*. the respective control set to 1. In (A_3), (C_2), (D_2; left panel), (E) control values were set to 100%, while in (D_2; right panel) the absolute values are reported. GAPDH probing (C_1) and *rp49* gene expression (C_5) were used as input reference. Bars, ± SD; *P < 0.05; **P < 0.01.

Figure S15. Graphical representation of non-targeted NMR-based metabolomics analysis in UAS CncC, UAS Gys^{RNAi}/$Gal4^{Tub}$ flies' somatic tissues. Results (depicted in box plots) are shown *vs*. the respective control of non-induced transgenes. Dashed line denotes the mean value found in CncC/Nrf2 overexpressing somatic tissues (also shown in Fig. S9) and can be directly compared to the value noted after combined inducible CncC/Nrf2 OE and Gys KD. A detailed description of the differences in metabolite concentrations observed in the NMR spectra (as depicted by z-score transformation) is reported in Table S7.

Figure S16. Suppression of IIS mitigates the transcriptional output of CncC/Nrf2 OE. Relative expression of the shown genes at the indicated transgenic fly lines. Samples were collected from young flies being exposed to 320 μM RU486 for 7 days. Gene expression was plotted *vs*. the respective control set to 1. Bars, ± SD; *P < 0.05; **P < 0.01.

Figure S17. Decreased IIS *via* dIlp2 KD suppresses CncC/Nrf2 activity and it partially rescues the CncC/Nrf2 OE-induced progeria. (A_1) Relative expression of the shown genes at the indicated two dIlp2 transgenic fly lines. (A_2) Relative expression of *rpn11*, *rpn6*, *α7* and *β5* genes at the shown transgenic lines; mean expression values/line/group of genes is also indicated. (B) Relative (%) 26S proteasome activities in CncC/Nrf2 overexpressing (or not) flies' tissues that also express dIlp2 RNAi. (C) Relative mitochondrial ST3/ST4 in flies expressing dIlp2 RNAi and overexpressing CncC/Nrf2. (D) Longevity curves of shown transgenic flies continuously exposed (or not) to oxidative stress (H_2O_2). (E) Longevity curves of transgenic flies expressing the shown transgenes. If not otherwise indicated samples were collected from young flies being exposed to 320 μM RU486 for 7 days. In (B) control values were set to 100%. Gene expression was plotted *vs*. the respective control set to 1; *rp49* gene expression, were used as reference. Bars, ± SD; *P < 0.05; **P < 0.01. **P of means < 0.01 *vs*. gene expression values found in RU486 induced UAS CncC/$Gal4^{Tub}$ flies (see Fig. 6B_1).

Figure S18. Our summarized findings highlight the notion that Nrf2 (a short-lived cellular stress sensor) is a central hub in the wiring of nutrients sensing, antioxidant and proteostatic signaling pathways. We report that Nrf2 induction activates dose-dependently a wide range of cytoprotective modules, modulates mitochondrial energetics and alters metabolic pathways. Although mild Nrf2 activation extends lifespan, prolonged high Nrf2 activity accelerates aging due to reprogramming of cellular bioenergetics and Diabetes Type 1-like metabolic stress. These effects can be partially alleviated by inhibition (red ┤ symbol) of anabolic pathways (e.g. IIS downregulation) that titrates CncC/Nrf2 activity (and thus emitted stress signaling) to lower levels.

Supporting Tables

Supporting Table S1. Summary of lifespan experiments.

Supporting Table S2. Proteins found to be differentially expressed [*vs*. control RU486(-) flies] in flies' somatic tissues after inducible (for 7 days) ubiquitous CncC/Nrf2 overexpression (nano-LC-ESI-MS/MS proteomics analysis).

Supporting Table S3. Human orthologs of fly proteins identified to be differentially expressed after inducible CncC/Nrf2 overexpression (nano-LC-ESI-MS/MS proteomics analysis).

Supporting Table S4. Proteins found to be increasingly ubiquitinated [*vs*. control RU486(-) flies] in flies' somatic tissues after inducible (for 7 days) ubiquitous CncC/Nrf2 overexpression (nano-LC-ESI-MS/MS proteomics analysis).

Supporting Table S5. Human orthologs of fly proteins identified to be increasingly ubiquitinated after inducible CncC/Nrf2 overexpression (nano-LC-ESI-MS/MS proteomics analysis).

Supporting Table S6. Differences [*vs*. control RU486(-) flies] of the shown metabolites concentration observed on the NMR spectra of UAS CncC/Gal4^{Tub} or UAS CncC^{RNAi}/Gal4^{Tub} RU486(+) flies (transgenes were induced for 7 days) as depicted by z-score transformation.

Supporting Table S7. Differences [*vs*. control RU486(-) flies] of the shown metabolites concentration observed on the NMR spectra of UAS CncC/Gal4^{Tub} or UAS CncC, UAS Gys^{RNAi}/Gal4^{Tub} RU486(+) flies (transgenes were induced for 7 days) as depicted by z-score transformation.

Supporting List of Abbreviations

ALP, Autophagy Lysosome Pathway; AMPK, AMP-activated kinase; ARE, Antioxidant Response Element; Atgl, Adipose Triglyceride Lipase/Brummer; C-L/β1, Caspase-Like proteasomal activity; CncC, Cap'-n'-collar isoform-C; CR, Caloric Restriction; CT-L/β5, Chymotrypsin-Like proteasomal activity; DDR, DNA Damage Responses; DT1, Diabetes Type I; ER, Endoplasmic Reticulum; dIlp, *Drosophila* Insulin-Like-Peptide; Foxo, Forkhead box O; G6P, Glucose 6-Phosphatase; GLU, Glucose; GLY, Glycogen; Gsk3β, Glycogen Synthase Kinase 3β; HSF1, Heat Shock transcription Factor-1; HSP, Heat Shock Protein; IIS, Insulin/IGF-like Signaling; ImpL2, Imaginal Morphogenesis Protein-Late 2; InR, Insulin Receptor; IPCs, Insulin Producing Cells; Keap1, Kelch-like ECH-Associated Protein 1; Nrf2, Nuclear factor erythroid 2-related factor; Pdpk1, 3-Phosphoinositide Dependent Protein Kinase-1; Pepck; Phosphoenolpyruvate Carboxykinase; PDR, Proteome Damage Responses; PN, Proteostasis Network; Pyk, Pyruvate Kinase; ROS, Reactive Oxygen Species; T-L/β2, Trypsin-Like proteasomal activity; Tgl, Triglyceride Lipase, phosphatidic acid phospholipase A1/Papla 1; ToR, Target of Rapamycin; TREH, trehalose; Ub, Ubiquitin; UPR^{ER}, ER Unfolded Protein Response; UPP, Ubiquitin Proteasome Pathway.

Key Resources Tables

REAGENT OR RESOURCE	SOURCE	IDENTIFIER
Antibodies		
anti-β5	M. Figueiredo-Pereira	N/A
anti-Atg8α	K. Koehler	N/A

anti-α-GLY	O. Babba	N/A
anti-Ref(2)P	G. Juhász	N/A
anti-dIlp2	Ernst Hafen	N/A
anti-ImpL2	Ernst Hafen	N/A
anti-20S-α	Santa Cruz Biotechnology	sc-65755
anti-Rpn10	Santa Cruz Biotechnology	sc-65748
anti-Rpn7	Santa Cruz Biotechnology	sc-65750
anti-phospho-Gsk3α/β$^{Y279/Y216}$	Santa Cruz Biotechnology	sc-11758
anti-Ubiquitin	Santa Cruz Biotechnology	sc-8017
anti-AMPKα	Santa Cruz Biotechnology	sc-25792
anti-β-Tubulin	Santa Cruz Biotechnology	sc-20852
anti-Histone 3	Santa Cruz Biotechnology	sc-8654
anti-H_2AvD	Rockland	600-401-914
anti-GFP	Santa Cruz Biotechnology	sc-9996
HRP-conjugated anti-mouse IgG	Santa Cruz Biotechnology	sc-2005
HRP-conjugated anti-rabbit IgG	Santa Cruz Biotechnology	sc-2004
HRP-conjugated anti-goat IgG	Santa Cruz Biotechnology	sc-2020
anti-Gsk3	Millipore S.A.	05-412
anti-GAPDH	Sigma-Aldrich	G9545
anti-FLAG	Sigma-Aldrich	F3165
anti Rabbit-IgG AlexaFlour 647	Jackson ImmunoResearch	711-605-152
anti-dFoxo	Cosmo Bio Co	CAC-THU-A-DFOXO
anti-Ndufs3	Abcam	ab14711
anti-Complex V subunit-ATP5a	Abcam	ab14748
anti-Grp75	Abcam	ab2799
anti-phospho-Gsk3$^{S21/S9}$	Cell Signaling	#9331
anti-phospho-Akt^{S505}	Cell Signaling	#4054
anti-Akt	Cell Signaling	#9272
anti-phospho-AMPKα^{T172}	Cell Signaling	#2535
Chemicals, Peptides		
ATP	Sigma-Aldrich	1905
DTT	Applichem	A2948
Glycerol	Applichem	A2926
Boric acid	Applichem	A2940
Trizma base	Sigma-Aldrich	T1503
TEMED	Applichem	A1148
Ammonium persulfate	Applichem	A1142
Sucrose	Applichem	A2211
EDTA	Applichem	A2937
BSA	Sigma-Aldrich	A9418
KH_2PO_4	Applichem	A1043
Hepes	Biosera	PM-B2093
Diethyl Malate	Sigma-Aldrich	W237418
Oligomycin	Sigma-Aldrich	75351
FCCP	Cayman Chemical	370-86-5
EGTA	Applichem	A0878
Triton X-100	Applichem	A4975
Paraformaldehyde	Scharlau	PA00950500
Glutaraldehyde	Applichem	A0589
MeOH	Scharlau	ME0316005l
NaN_3	Applichem	A1430
KCl	Applichem	131494
$CDCl_3$	Applichem	133101

Chloroquine diphosphate salt	Sigma-Aldrich	C6628
PS-341	Millennium Pharmaceuticals	Velcade
RU486	Sigma-Aldrich	M8046
Protein-A Sepharose beads	GE Healthcare	17-0974-01
Suc-Leu-Leu-Val-Tyr-AMC-LLVY	Enzo Life Sciences	BML-P802-0005
Z-Leu-Leu-Glu-AMC-LLE	Enzo Life Sciences	BML-ZW9345-0005
Boc-Leu-Arg-Arg-AMC-LRR	Enzo Life Sciences	BML-BW8515-0005
z-FR-AMC	Enzo Life Sciences	BML-P139-0050
G250	Invitrogen	BN2004
Amyloglucosidase	Sigma-Aldrich	A7420
Trehalase	Sigma-Aldrich	T8778
Critical Commercial Assays/Kits		
Bradford assay	Bio-Rad	5000006
OxyBlot	Millipore	#s7150
iTRAQ kit	ABSciex	4393529
Native sample buffer	Invitrogen™	BN20032
GLU Reagent	Sigma-Aldrich	GAGO-20
Mowiol®	Sigma-Aldrich	4-88
Genomic DNA Kit	Thermo Fisher Scientific	#K0512
DreamTaq Green PCR Master Mix	Thermo Fisher Scientific	#K1082
Maxima First Strand cDNA Synthesis Kit	Thermo Fisher Scientific	#K1642
SYBR Green/ROX qPCR Master Mix	Thermo Fisher Scientific	#K0223
Nitrocellulose membrane	Macherey-Nagel GmbH	741280
PVDF membrane	Macherey-Nagel GmbH	741290
Fluorescent dyes		
BODIPY	Molecular Probes	D3922
DAPI	Molecular Probes	D1306
Phalloidin	Molecular Probes	R415
Experimental Models: *Drosophila* lines		
UAS CncC	D. Bohmann	Sykiotis and Bohmann, 2008
UAS CncC$^{\Delta 1\text{-}87}$	D. Bohmann	Sykiotis and Bohmann, 2008
UAS CncC^{RNAi}	D. Bohmann	Sykiotis and Bohmann, 2008
UAS Keap1	D. Bohmann	Sykiotis and Bohmann, 2008
UAS Keap1^{RNAi}	D. Bohmann	Sykiotis and Bohmann, 2008
gstD-ARE:GFP/II	D. Bohmann	Sykiotis and Bohmann, 2008
gstD-mARE:GFP/III	D. Bohmann	Sykiotis and Bohmann, 2008
Tubulin GeneSwitch Gal4	D. Bohmann	Sykiotis and Bohmann, 2008
UAS Atg8α	BDSC	ID: 51656
UAS Gys^{RNAi}	BDSC	ID: 34930
UAS Pdpk1^{RNAi}	BDSC	ID: 34936
UAS Inr^{RNAi}	BDSC	ID: 51518
UAS Keap1^{RNAi}	BDSC	ID: 57801
UAS dIlp2^{RNAi}	BDSC	ID: 32475
UAS mCherry-Atg8α	A. Daga	N/A
UAS GFP-Lamp1	A. Daga	N/A
UAS Mito^{GFP}	A. Daga	N/A
Gal4^{Actin}	A. Daga	N/A
Gal4^{Elav}	A. Daga	N/A
Gal4^{D42}	A. Daga	N/A
Gal4^{Mef2}	A. Daga	N/A
Gal4^{MHC}	A. Daga	N/A
Isogenic UAS CncC	This study	N/A

Isogenic tubGSGal4	This study	N/A
UAS CncC, UAS dIlp2^{RNAi}	This study	N/A
UAS CncC, UAS Pdpk1^{RNAi}	This study	N/A
UAS CncC, UAS Inr^{RNAi}	This study	N/A
UAS CncC, UAS Atg8α	This study	N/A
UAS CncC, UAS Gys^{RNAi}	This study	N/A
Oligonucleotides		
See Method Details for primer sequences	This study	N/A
Software and Algorithms		
MS Excel	Microsoft	N/A
IBM SPSS; version 19.0	IBM	N/A
ImageJ	Wayne Rasband (NIH)	N/A
Digital Eclipse Nikon C1 software	Nikon Inc.	N/A
INGENUITY® PATHWAY ANALYSIS	QIAGEN Bioinformatics	N/A
ICON-NMR v. 4.2.6.	Bruker Biospin	N/A
AMIX Statistics v. 3.9.14	Bruker Biospin	N/A
SIMCA-P+ v. 11.5	Umetrics AB, Sweden	N/A

Supporting Experimental Procedures

Locomotion (climbing), UV-exposure and longevity assays

The mobility of flies was assayed as described previously (Tsakiri, Sykiotis, Papassideri, Terpos, et al., 2013). Briefly, 30 flies (15 males and 15 females) were placed in a 100-ml cylinder with a line drawn at the 66 ml mark. Flies were gently tapped to the bottom of the cylinder and the number of flies that climbed above the 66 ml mark after 20 s was recorded. Larvae were exposed (or not) to UV for 30 min. In oxidative stress assays adult flies were treated with 0.8% H_2O_2 added in culture medium.

For longevity assays female and male flies (equal numbers per sex) were collected and cultured in vials; flies were transferred to vials with fresh food every 3-4 days and deaths were scored every day.

Genomic DNA Extraction and conventional PCR analyses

To verify the establishment of transgenic flies carrying more than one transgene, genomic DNA from larvae or flies' tissues was extracted with the Genomic DNA Kit of Thermo Fisher Scientific (#K0512). DNA was then subjected to conventional PCR analysis using the DreamTaq Green PCR Master Mix of Thermo Fisher Scientific (#K1082). PCR products were analyzed by agarose gel electrophoresis where needed. Primers where designed using the primer-BLAST tool (http://www.ncbi.nlm.nih.gov/tools/primer-blast/) and were the following: *Valium 20*-F: ACCAGCAACCAAGTAAATCAAC, *Valium 20*-R: TAATCGTGTGTGATGCCTACC; *cncC*-F: TGGAATTGGGCACCCATGGCG, *cncC*-R: AGTTTGAGTACGTCGTTCAACA

Total RNA extraction and Quantitative Real-Time PCR (Q-RT-PCR) analyses

Extracted total RNA was converted to cDNA with the Maxima First Strand cDNA Synthesis Kit of Thermo Fisher Scientific (#K1642). cDNA was then subjected to Q-RT-PCR analysis using the SYBR Green/ROX qPCR Master Mix of Thermo Fisher Scientific (#K0223) as described previously (Tsakiri *et al.*, 2017). Primers were designed using the primer-BLAST tool (http://www.ncbi.nlm.nih.gov/tools/primer-blast/) and were the following:
rpn11-F: ACAACAAGTCACTGGAGGACG, *rpn11*-R: TGCTTGCCCACGTTCTTGAT; *rpn10*-F: TGCCTTCGCTATGCAGATGT, *rpn10*-R: GTTTGCCTCGTCCGTCTTTG; *rpn6*-F: TCTACTGTCCGCCAAAGGTG, *rpn6*-R: TTCCACTGACGAGCTGGTTG; *α5*-F: ACGCCAGAAGGAGTGGTTTTG, *α5*-R: GCTTGTCCACCTCCACAATCT; *α7*-F: ACCGACGAATTGGTGGAGAG, *α7*-R: ACCCATTTCGAAGCGGAAGT; *β1*-F: GCGACGCATCTCTACAACAC, *β1*-R: CGAGGAAATGAAGCTGGGAGT; *β2*-F: AGCCACCGACCACCACCAAGA, *β2*-R: CCACAACGCGCACCTCACGA; *β5*-F: GCCATCTACCATGCCACCTT, *β5*-R: TTACCCAGCCGTCCTCCTTA; *atg6*-F: GTTCCTGCTGCCCTACAAGA, *atg6*-R: TCCACTGCTCCTCCGAGTTA; *atg8α* -F: ACGCCTTCGAGAAGCGTCGC, *atg8α*-R: CCAAATCACCGATGCGCGCC; *ref(2)P*-F:

GGTCAGCTGGGCGAACTATT, *ref(2)P*-R: ATCAGTGTGGTAGACGGGGA; *hdac6*-F: TGCTGGAGCGCATTTACCTT, *hdac6*-R: ATGGTATGGTGACTGGTGCC; *hsp70*-F: AAGAACCTCAAGGGTGAGCG, *hsp70*-R: CGTCGATGGTCAGGATGGAG; *grp78* (*hsc70-3*)-F: ATATTACTGGCCGTCGTGGC, *grp78* (*hsc70-3*)-R: CACACCAACGCAGGAATACG; *cncC*-F (conventional PCR): TGGAATTGGGCACCCATGGCG, *cncC*-R: ATCATTGAGGGCGGCGGTGC; *cncC*-F (Q-RT-PCR): CCAACGAGGTGGAAATCGGA, *cncC*-R: ACTTGACATTGGTAGCCGCA; *keap1*-F: GCGCTCGTCAGCCCATTTT, *keap1*-R: GGATGCGCATAATTCCTCTTCTT; *maf-S*-F: AAAGTCATCCCTGGCACCAC, *maf-S*-R: CATCTGCTCCAGTTCCGTCC; *gstd1*-F: TGATCAATCAGCGCCTGTACT, *gstd1*-R: GCAATGTCGGCTACGGTAAG; *trxr-1*-R: ATTTTGAGTGCATGTCGGCG, *trxr-1*-F: GACACCAGTGGAACTACCCG; *inr*-F: CAGCTGTCAAGCAAGCAGTG, *inr*-R: GTCTGCGGCACAGTACGATA; *pdpk1*-F: TTCAAACCAGTCGCTTCGTG, *pdpk1*-R: TGTGTATGTATATCAATGCCAGCG; *tor*-F: TTTATCCTCCGTGAGCTGGC, *tor*-R: CTGTGGTTCACTGGACTGCT; *akt*-F: ATTCAGCTGGCAGCAATCCT, *akt*-R: AAGCGCATGACCCGATGTAA; *g6p*-F: GTCGGTGCGAGAGGCATTTA, *g6p*-R: TATAGTGGCGAGGCCAAAGC; *pepck*-F: TGAACGCAAAGTCCTCGACA, *pepck*-R: ACATTGCCGGAGATAATTTTGC; *sgg*-F: TGCGCAAATTGGAGCATTGT, *sgg*-R: TGTTCTCTTGTCGGTGTGCC; *gys*-F: TCACTTTTACGGCCACATTGA, *gys*-R: AGCGAGTCCACGTTGAAGTT; *gyp*-F: GTG-TCC-ACC-CTG-AGG-GAC-TA, *gyp*-R: AGACCCAACTGGTACATGGC; *pyk*-F: CGCATTGTCACCGTCGAATA, *pyk*-R: AGCCTCCTGCAGTTTCCAAT; *pdk*-F: CATGGACTTTGGTCAGAATGC, *pdk*-R: TCGCTTACGGATCTGGTGTG; *pdp*-F: CATACGATGTTAACCTTGTTCTGAG, *pdp*-R: TGCCACAAATGAAGCCGTTG; *pek*-F: TCGTGCGATACTTCCATTCC, *pek*-R: AGTCGTGACTACAAGCGGTG; *tps1*-F: AGGGCCAGTGCCGGT, *tps1*-R: GTCGGAGTCTGATCGTTGGG; *treh*-F: GACAGGCACATGTACGAGAAGT, *treh*-R: AGCCACAACCGGATCCAATG; *dilp2*-F: ATCCCCTGCAGTTTGTCCAG, *dilp*2-R: ACCTCTCCACGATTCCTTGC; *dilp6*-F: CAAAGTGCCGACGTCCAAAG, *dilp6*-R: AAATACATCGCCAAGGGCCA; *impL2*-F: CCGCGAATCATCTACACCGA, *impL2*-R: TTCAACCAGGTGATCTCGGC; *atgl*-F: TCCTTGTGGATGAATGTGGAG, *atgl*-R: GGGGATTGGGGTGATCTTCG; *tgl*-F: CACCCAAGTTCTACGCACCT, *tgl*-R: AGGGTTTGCTGCGTACGTTA; *marf*-F: CCGCTATCCCGGTTCAACTC, *marf*-R: AGCGGTGATTTGTCGTTGGA; *drp1*-F: CAACGCACGTGGTCAACCTA, *drp1*-R: GATCTGCGCCTCGATATCCTC; *opa1*-F: CGAGGAGTTCCTACTTGC, *opa1*-R: TGAGATTCCGCGAGAACTGG; *miro*-F: ACTGCCATCCCCATCACTCT, *miro*-R: TGAGATATGCCGCTCCTGTG; l*on*-F: TCTCGCGAGTGCTCTTCATC, *lon*-R: ATTCCCGGCAATAACTGCGA; *sdhA*-F: CACCGGTGGATATGGACGAG, *sdhA*-R: CCATAGCAGTACCGTCACCG; *ATPsynβ*-F: CCCGTGGTGTGCAGAAAATC, *ATPsynβ*-R; AAACGCTGAATCTTGCGAGC; *p47*-F: TGTCATGCGCGGGGAAAT, *p47*-R: CTAGGCGAATCTGCAGCGTA; *ter94*-F: GCTATCGCAGGCGAAGATG, *ter94*-R: TGCACGGAAACCACATCTGA; *aos1*-F: GCTACGTCGAGGATGTTATTAAGC, *aos1*-R: TGCGGAACTTTTGCAGGACA; *ufd1*-F: AGGGCGGAAAGATTATTATGCCT, *ufd1*-R: GGTGATGTCCAGGAAGTCGG; *uch*-F: GGCCAACAACAAAGAAGTGGA, *uch*-R: CTCCTTGTTCACCAGTGCGA. The ribosomal gene *rp49* (*rp49*-F: AGCACTTCATCCGCCACC, *rp49*-R: ATCTCGCCGCAGTAAACG) was used as a normalizer.

Chromatin immunoprecipitation (ChIP)

For ChIP analyses somatic tissues (100 isolated heads/thoraces) were fixed with fixation buffer (50 mM HEPES pH 7.6, 100 mM NaCl, 0.1 mM EDTA pH 8.0, 0.5 mM EGTA pH 8.0, 4 % formaldehyde) for 1 hr at RT. Fixed tissues were homogenized in nuclear lysis buffer (50 mM HEPES pH 8.0, 10 mM EDTA, 0.5 % N-lauryl-sarkosin, 1x fresh protease inhibitor cocktail, 1 mM PMSF) and were sonicated for 30 sec with a Vibracell^{TM} sonicator (Sonic and Materials InC, Danbury, Connecticut USA) for 16 cycles (30 sec on/ 60 sec off). Lysates were cleared of debris by centrifugation for 10 min at 20,000 x g at 4°C; gel electrophoresis revealed a smear of small DNA fragments of an average size of ~200-350 nucleotides. For immunoprecipitation, an equal volume of 2x RIPA buffer was added and lysates were precleared for non-specific binding by adding Protein A

agarose beads for 1 hr at 4° C. Mouse anti-FLAG (for FLAG-tagged CncC/Nrf2 immunoprecipitation) or goat anti-Histone 3 antibodies were added overnight at 4°C; a mock sample (without antibody addition) was also performed. Protein A agarose beads were blocked with 1mg/ml BSA in RIPA overnight and were added to the lysate/antibody mixture for 3 hrs at 4°C; washed once for 10 min in RIPA buffer, four times in RIPA buffer containing 0.5M NaCl, once in LiCl buffer (250 mM LiCl, 10mM Tris-HCl pH 8, 1mM EDTA, 0.5% NP-40, 0.5% sodium deoxycholate) and once in TE pH 8.0. Chromatin was harvested by digestion with 50μg/ml RNase A for 30 min at 37°C; then 200 mM NaCl were added and the cross-linking was reversed by overnight incubation of the samples at 65° C. The following day, after proteinase K treatment, the DNA was recovered by phenol/chloroform extraction and ethanol precipitation. Q-RT-PCR was performed as described above. Binding enrichment was calculated as fold enrichment *vs*. the mock sample, while binding to the genomic regions of *u1*, *rp49* and *gapdh* genes was used as negative control. Primers used were the following:
*impL2*_1-F: *TAACTGCAACTGCAGCCTGA*, *impL2*_1-R: *GCTTCGTTGGCACTTTCACT*; *impL2*_2-F: *CTCCGTTTGACTCATCGCCT*, *impL2*_2-R: *CCCTTTGTACCCACGTCACA*; *impL2*_4-F: *ACCAAGTCCACGTCCACATC*, *impL2*_4-R: *GCGCTTCAGGATCGGGATAA*; *impL2*_5-F: *CCGCGAGTCATTCCGTTTTC*, *impL2*_5-R: *GCTCTCGGAGTCCGGAATTT*; *impL2*_6-F: *GGCTATTGGATTGACGACGGA*, *impL2*_6-R: *AGCCGGCATTAGATACGGTT*; *u1*-F: *CCGAGAGTGATGAGCATTTGCC*, *u1*-R: *AACTTGCTCCCCTTTTGCGTGG; rp49*-F: *TCCTTCCAGCTTCAAGATGAC*, *rp49*-R: *GTGCGCTTCTTCACGATCT; gapdh*-F: *CATTCTCCTAATTTGCGA*, *gapdh*-R: *AATTCCGATCTTCGACATGG*.
rpt6-F: *GGCATGTGTCATCTCGAAAAGC*, *rpt6*-R: *GGACCACAATGCAAGATGTCG; cathD*-F: *AGCCACCAGAGAAGCGTAAC*, *cathD*-R: *GCCAGGAAGGCGACTAGAAG*; *p47*_1-F: *CGGCGATTTGATTGCACAGT*, *p47*_1-R: *CCTGGGTGCTCCAGTAGTTG*; *p47*_2- F: *ACCAAGGCCAAGCCAAAGTA*, *p47*_2-R: CCACCTCTGCGATGTTTTGC; *p47*_3-F: *GCAAGAGCTGCTGGAAATGG*; *p47*_3-R: *GTTAATGGCATCACGGGCAC; p47*_4-F: *CGTCTCCGACATTCGTCGTT*, *p47*_4-R: *GTTCTTGAGGCCAGCCTTCT*; *hsp26*_1-F: *GCTTGACGAACAGAGCACAG*, *hsp26*_1-R: *GGCTCCTGGAGTTCATCCAC*; *hsp26*_2-F: *TCCAAGGAGCGCATCATTCA*, *hsp26*_2-R: *TACTTGTCCTTGCCGTTGGG*; *ref(2)P*_1-F: *AGCACCGTGATTTGCAACAA*, *ref(2)P*_1-R: *TACAAGTGCAGCTGGGTGAG*; *ref(2)P*_2-F: *GCCCAACAAAGCAGGGATTC*, *ref(2)P*_2-R: *GTTGTACCAGCACAACCCGT*; *ref(2)P*_3-F: *AGCTTAGTTTTTCTGTGGTGCT*, *ref(2)P*_3-R: *TGTGCCTTTATTGCAATTTCACAC*; *ter94*_1-F: *CTGTGTCGGTCCAAGTCGAA*, *ter94*_1-R: *CAAATGGCGATTCTCGCTGG*; *ter94*_2-F: *CGGCAGGCCTAATTATCCAGA*, *ter94*_2-R: *TCTTATCGTTCTTGCCGCC*; *ter94*_3-F: *TTGGCTAAGACATCCAGCCG*; *ter94*_3-R: *AGCCACTCTACCGACAATGC*; *impL2*_1-F: *TAACTGCAACTGCAGCCTGA*, *impL2*_1-R: *GCTTCGTTGGCACTTTCACT*; *impL2*_2-F: *CTCCGTTTGACTCATCGCCT*, *impL2*_2-R: *CCCTTTGTACCCACGTCACA*; *impL2*_4-F: *ACCAAGTCCACGTCCACATC*, *impL2*_4-R: *GCGCTTCAGGATCGGGATAA*; *impL2*_5-F: *CCGCGAGTCATTCCGTTTTC*, *impL2*_5-R: *GCTCTCGGAGTCCGGAATTT*; *impL2*_6-F: *GGCTATTGGATTGACGACGGA*, *impL2*_6-R: *AGCCGGCATTAGATACGGTT*; *keap1*-F: *AAGGTTTTGCCTCTTCAGCA*, *keap1*-R: *CGGCTTTAAATTTCCGCATA*; *gstd1*-F: *CATGTGCCTTTGACTCATCATT*, *gstd1*-R: *GACCATAAAAATATAACCGTTTTTCG*; *gapdh*-F: *CATTCTCCTAATTTGCGA*, *gapdh*-R: *AATTCCGATCTTCGACATGG*; *u1*-F: *CCGAGAGTGATGAGCATTTGCC*, *u1*-R: *AACTTGCTCCCCTTTTGCGTGG; rp49*-F: *TCCTTCCAGCTTCAAGATGAC*, *rp49*-R: *GTGCGCTTCTTCACGATCT*.

Full names of analyzed genes

akh, Adipokinetic hormone; *akt1*, Akt1; *aos1*, Activator of SUMO 1; *atg6*, Autophagy-related 6; *atg8a*, Autophagy-related 8a; *atgl*, adipose triglyceride lipase (*atgl/brummer*); *ATPsynβ*, ATP synthase β subunit; *dilp2*, Insulin-like peptide 2; *dilp6*, Insulin-like peptide 6; *drp1*, Dynamin related protein 1; *g6p*, Glucose-6-Phosphatase: *gyp*, Glycogen phosphorylase (*glyp*); *gys*, Glycogen synthase (*glys*); *gstd1*, Glutathione S transferase D1; *grp78*, Heat shock 70-kDa protein cognate 3 (*hsc70-3*); *hdac6*, Histone deacetylase 6; *hsp70*, Heat shock protein 70; *ide*, Insulin degrading metalloproteinase; *impL2*, Imaginal morphogenesis protein-Late 2 (Ecdysone-inducible gene L2); *inr*, Insulin-like receptor; *keap1*, Kelch-like ECH-associated protein 1; *lon*, Lon protease; *maf-S*, Musculoaponeurotic fibrosarcoma oncogene; *marf*, Mitochondrial assembly regulatory factor; *miro*, Mitochondrial Rho;

opa1, Optic atrophy 1; *p47*, p47; *pdk*, Pyruvate dehydrogenase kinase; *pdp*, Pyruvate dehydrogenase phosphatase; *pdpk1*, 3-Phosphoinositide-dependent protein kinase 1; *pek*, Pancreatic eIF-2α kinase; *pepck*, Phosphoenolpyruvate carboxykinase (*pepck1*); *pyk*, Pyruvate kinase; *ref(2)P*, RNA and export factor binding protein 2; *rp49*, Ribosomal protein L32; *rpn10*, Regulatory particle non-ATPase 10; *rpn11*, Regulatory particle non-ATPase 11; *rpn6*, Regulatory particle non-ATPase 6; *sdhA*, Succinate dehydrogenase, subunit A; *sgg*, Shaggy; *ter94*, Valosin-containing protein (p97); *tgl*, Phosphatidic Acid Phospholipase A1 (triglyceride lipase, *palpa1*); *tor*, Target of rapamycin; *tps1*, Trehalose-6-phosphate synthase 1; *treh*, Trehalase; *tret1-2*, Trehalose transporter 1-2; *trxr-1*, Thioredoxin reductase-1; *uch*, Ubiquitin carboxy-terminal hydrolase; *ufd1-like*, Ubiquitin fusion-degradation 1-like; *α5*, Proteasome α5 subunit (*prosα5*); *α7*, Proteasome α7 subunit (*prosα7*); *β1*, Proteasome β1 subunit (*prosβ1*); *β2*, Proteasome β2 subunit (*prosβ2*); *β5*, Proteasome β5 subunit (*prosβ5*).

Preparation of tissue protein extracts, immunoblot analysis, immunoprecipitation and detection of protein carbonyl groups

Tissue extracts preparation, adjustment of protein content of each sample lysate by Bradford assay (Bio-Rad) and immunoblotting were performed as described previously (Tsakiri, Sykiotis, Papassideri, Terpos, et al., 2013). Analysis of blots quantification was done by scanning densitometry.

Hemolymph was isolated from equal numbers of young male and female flies according to standard procedures as described previously (Tsakiri, Sykiotis, Papassideri, Gorgoulis, et al., 2013). For the detection of dIlp2 and ImpL2 proteins by immunoblotting, protein extracts from dissected heads or isolated hemolymph (obtained from equal number of female and male flies) were separated on 16% polyacrylamide Tris-Tricine-SDS gels.

For proteasome immunoprecipitation analyses, tissue protein extracts were cleared by adding protein-A Sepharose beads (Code Number: 17-0974-01, GE Healthcare) and the target antigen was then overnight immunoprecipitated at 4ºC with 2 μg of the corresponding antibody, followed by the addition of protein-A sepharose beads for 2 hours at 4°C. Immunoprecipitated protein complexes were then analyzed by immunoblotting.

For the detection of protein carbonyl groups, the OxyBlot protein oxidation detection kit (Millipore, Billerica, MA; #s7150) was used, as per manufacturer's instruction.

Measurement of reactive oxygen species (ROS), proteasome and cathepsin B, L enzymatic activities in tissue extracts

Tissue extracts preparation and measurement by fluorometry (using a VersaFluor Fluorometer System; Bio-Rad Laboratories, Hercules, CA, USA) of ROS, as well as of proteasome and cathepsin B, L enzymatic activities were done as described previously (Tsakiri, Sykiotis, Papassideri, Terpos, et al., 2013). Fluorescence intensity was normalized to the total protein level per sample and expressed as the relative percentage *vs.* the corresponding control; in adult flies, equal numbers of male and female flies were used.

In-gel proteasome activity detection

For *in-gel* proteasome detection and activity viewing flies' somatic tissues were lysed in 50 mM Tris-HCl (pH 7.4), 5 mM $MgCl_2$, 5 mM ATP (grade 1; Sigma, St. Louis, MO, USA) with 1 mM DTT and 10% glycerol. Protein content was measured with Bradford assay and equal protein amount of xylene cyanol dyed samples were loaded to a 3-5 % acrylamide gradient non-denaturing minigel. Gel buffer contained 0.18 M boric acid, 0.18 M Trizma base, 5 mM $MgCl_2$, 1 mM ATP, 1 mM DTT, Rhinohide polyacrylamide gel strengthener and the appropriate amount of acrylamide; gels were polymerized with 0.1%N,N,N′,N′-tetramethylethylenediamine (TEMED) and 0.1% ammonium persulfate (APS). Non-denaturing minigels were run at 125 V for 3 h (4°C) and were then incubated for 30 min (at 37°C) in a buffer containing 1mM ATP and 0.3 mM of the proteasome substrate LLVY-AMC. The emitted fluorescence was observed in a UV light recorder. Native proteins were transferred for 2.5h (110 mA) onto a nitrocellulose membrane and probed with the indicated antibodies; to verify equal loading dyed samples were boiled and analyzed by SDS-PAGE.

Mitochondria isolation and measurement of mitochondrial respiration

For mitochondria isolation somatic tissues of flies were homogenized in ice-cold isolation buffer (0.32 M sucrose, 10 mM EDTA, 10 mM Tris/HCl, pH 7.3) containing 2% (w/v) BSA. Samples were filtered through a layer of gauze which was then washed with additional isolation buffer up to final

volume of 1.5 ml. Following centrifugation for 10 min at 2,200 xg the pellet was washed with BSA-free isolation buffer and resuspended in 200 µl of the same buffer. The protein content of isolated mitochondria was measured by the Bradford method.

Respiration of isolated mitochondria was determined using a Clark-type oxygen electrode connected to a computer-operated Oxygraph control unit (Hansatech Instruments, Norfolk, UK). In brief, freshly isolated mitochondria (150 µg of protein) were added to the respiration buffer (120 mM KCl, 5 mM KH_2PO_4, 3 mM Hepes, 1 mM EGTA, 1 mM $MgCl_2$ and 0.2% BSA, pH 7.2) containing 5 mM glutamate/2.5 mM malate. Basal O_2 consumption was recorded (*State 2*) and after 2 min 500 µM of ADP was added (*State 3*; indicates rate of ATP production, O_2 consumption*)*, followed by the addition of 6 µM of the ATP synthase inhibitor oligomycin (*State 4*; denotes coupling) and 100 nM of the uncoupler (causes maximal respiration) carbonyl cyanide p-trifluoromethoxyphenylhydrazone (FCCP) (*State FCCP*). For all the experiments, the temperature was maintained at 25°C and the total reaction volume was 300 µl. The Respiratory Control Ratio (RCR) was calculated as the ratio of State 3/State 4 (*ST3/ST4*).

Blue Native PAGE (BN-PAGE) for mitochondrial Respiratory Chain Supercomplexes (RCS)

For BN-PAGE and RCS analyses isolated mitochondria (250 µg) from adult flies' somatic tissues were suspended in BSA-free mitochondria isolation buffer and centrifuged at 10,000 ×g (4°C). The pellet (mitochondria) was resuspended in 100 µl 1X Native sample buffer (BN20032, Invitrogen^{TM}) containing 4% digitonin and incubated for 10 min on ice before re-centrifuging at 20,000 ×g (4°C, 30 min). The supernatant (extracted solubilized complexes) was collected and the Native PAGE sample additive G250 5% (BN2004 Invitrogen^{TM}) was added. Samples were separated by 3-12% gradient BN-PAGE and after electrophoresis the complexes were transferred on a polyvinylidene fluoride (PVDF) membrane; PVDF membranes were then probed with the indicated antibodies.

Measurement of GLU, TREH and GLY levels

For GLU, TREH and GLY measurement, somatic tissues of 6 flies (3 males and 3 females) were homogenized either in cold PBS for GLU or GLY measurement, or in Trehalase buffer (5 mM Tris pH 6.6, 137 mM NaCl, 2.7 mM KCl) for TREH measurement. Extracts were cleared by centrifugation at 1,200 ×g and the supernatant was incubated for 10 min at 70°C; a small amount of the cleared extract was used for protein quantification by Bradford assay. After centrifugation at max speed for 3 min, 30 µl of diluted (1/4) (no sample dilution was done in the case of TREH measurement) supernatant was transferred to a 96-well plate. GLU measurement was performed by adding 100 µl of GLU Reagent (Sigma, GAGO-20) followed by 30 min incubation at 37ºC. For GLY measurement, the same procedure as for the GLU assay was followed except that the samples were incubated with or without 1 unit of amyloglucosidase (Sigma, A7420). For TREH measurement 100 µl of GLU reagent were added and samples were incubated with or without 0.05 units/ml of trehalase (Sigma, T8778) for 18 hours at 37ºC. Absorbance was recorded at 540 nm and the TREH or GLY levels were calculated after the substraction of the GLU measured at this step from the total amount of free GLU measured after trehalase or amyloglucosidase digestion. At least 3 replicates per genotype or experimental condition were performed.

Larvae body wall or fat body preparations and dissection of adult brain, muscles or fat body for immunofluorescence and Confocal Laser Scanning Microscope (CLSM) visualization

Third instar larvae were collected and dissected in PBS. Larvae were placed into a Petri anatomy dish and immobilized with two small pins (one to the top and one to the edge of larvae) so that the abdominal region was upwards. Larvae were cut just above the edge and then a slit was made until the larvae mouth to enable removal of the internal organs. The body of the larvae was then opened and stretched with small pins.

Isolation of brains from adult flies started by tearing the connective tissue between the proboscis and the eye, of dissected heads. After tearing away the retina and the cuticle and removing the trachea, the intact brain was isolated. For adult muscles dissection, individual flies were grabbed from their wings by forceps and were placed into a petri anatomy dish. Head and abdomen were pinned down and using the forceps the thorax was opened in order to remove the exoskeleton. Head and abdomen were also removed, and PBS was added into muscles. Then, muscles were separated to distinct muscle fibers. Dissected larvae or adult tissues were fixed in 4% formaldehyde for 15 min, washed in PBS

containing 0.3% Triton X-100 and incubated with primary antibody overnight at 4ºC. Secondary antibodies, DAPI or Phalloidin (Thermo Fisher Scientific; for nuclear and actin cytoskeleton visualization, respectively) staining were applied for 1 hour at RT. Following three washes with PBS, samples were mounted in Mowiol® 4-88 (Sigma) and viewed in a Digital Eclipse Nikon C1 (Nikon, Melville, NY, USA) CLSM equipped with 40× 1.0 NA differential interference contrast (DIC), and 60× 1.4 NA DIC Plan Apochromat objectives; image capturing was done using the EZC1 acquisition and images were analyzed with the CLSM software (Nikon Inc.). Z-stacks with a step size of 0.5 μm were taken using identical settings. Each stack consisted of 15 to 20 plane images (at least 10 animals per genotype were viewed and representative captures are shown).

For boron-dipyrromethene fluorescent dye (BODIPY 493/503; Molecular Probes™ - Thermo Fisher Scientific) staining of larvae fat body, the procedure of larvae body wall preparation was followed without removing the fat body. For BODIPY staining of adult fat body, anesthetized flies were placed (dorsal side down) into a Petri dish coated with a thin layer of petroleum jelly. The head and thorax were removed with a single cut. Using small scissors, the posterior tip of the abdomen was also removed with a single cut. Then, a cut along both edges of the abdomen was made and abdominal organs were carefully removed without affecting the fat body. Dissected larva or adult fat body were fixed in 4% formaldehyde for 15 min, washed in PBS containing 0.3% Triton X-100 and incubated with the BODIPY dye or DAPI (Thermo Fisher Scientific) for 15 min at RT. After three washes with PBS, samples were mounted in Mowiol® 4-88 (Sigma) and viewed in CLSM. Measurement of CLSM stained structures (e.g. lipid particles or mitochondria) was done by ImageJ (NIH).

Electron microscopy

Sample preparation and Electron microscopy (EM) viewing was done according to standard procedures. Briefly, muscles of adult flies were incubated for 2 hrs (at RT) in fixation solution containing 4% paraformaldehyde, 2% glutaraldehyde in 0.1 mM sodium cacodylate buffer, pH 7.2, and were then washed overnight at 4 °C. Samples were post-fixed with 1% OsO_4 and were then embedded in Epon. EM images were acquired from thin sections using a transmission electron microscope Tecnai 12 (FEI, North America NanoPort, Hillsboro, Oregon, USA).

Sample preparation and nano-LC-ESI-MS/MS analysis of iTRAQ labeled peptides

Flies' somatic tissues were homogenized with the dissolution buffer (0.5 M triethylammonium bicarbonate) from the iTRAQ kit (ABSciex), mixed with vortex and after the addition of 0.05 % SDS they were sonicated (20% power with 0.1-0.2 pulses) for 20 sec in ice. Homogenates were then centrifuged for 15 min at 13,000 ×g (4° C); supernatant was collected, and protein content was measured with the Bradford assay. 50 μg of recovered protein were diluted in dissolution buffer (solution C) up to a final volume of 20 μl, followed by the addition of 2 μl reducing reagent from the iTRAQ kit. Samples were then incubated for 1 h at 60°C. Following the addition of 1 μl cystein blocking reagent (200 mM methyl methanethiosulfonate in isopropanol) from the iTRAQ kit, samples were incubated for 10 min at RT. Proteins were digested with overnight incubation (at RT in dark) after the addition of 75 ng/μl trypsin (protein/trypsin 30:1) and were subsequently labeled with the iTRAQ reagents as per manufacturer's instructions.

LC-MS experiments were performed on a Dionex Ultimate 3000 UHPLC system coupled with the high resolution nano-ESI Orbitrap-Elite mass spectrometer (Thermo Fisher Scientific). Individual high-pH RP peptide fractions were reconstituted in 50 μl loading solution composed of 0.1 % formic acid. A 5 μl volume was injected and loaded on the Acclaim PepMap 100, 100 μm × 2 cm C18, 5 μm, 100 Å trapping column with the ulPickUp Injection mode; the loading pump was operating at flow rate of 5 μl/min. For the peptide separation, the Acclaim PepMap RSLC, 75 μm × 50 cm, nanoViper, C18, 2 μm, 100 Å column retrofitted to a PicoTip emitter was used for multi-step gradient elution. Mobile phase (A) was composed of 0.1 % formic acid and mobile phase (B) was composed of 100% acetonitrile, 0.1% formic acid. The peptides were eluted under a 315-minute gradient from 2% (B) to 33% (B). Flow rate was 300 nl/min and column temperature was set at 35°C. Gaseous phase transition of the separated peptides was achieved with positive ion electrospray ionization applying a voltage of 2.5 kV. For every MS survey scan, the top 10 most abundant multiply charged precursor ions between m/z ratio 300 and 2200 and intensity threshold 500 counts were selected with FT mass resolution of 60,000 and subjected to HCD fragmentation. Tandem mass spectra were acquired with FT resolution

of 15,000. Normalized collision energy was set to 33 and already targeted precursors were dynamically excluded for further isolation and activation for 45 sec with 5 ppm mass tolerance.

Flies' tissues extraction, preparation of samples for Nuclear Magnetic Resonance (NMR) and NMR-based metabolomics analysis

Tissues extraction and NMR sample preparation was done according to the following protocol that allows the collection of polar and lipid metabolites separately. Briefly, liquid nitrogen snap frozen somatic tissues were homogenized with 300 μl ice cold $CHCl_3$/MeOH (2:1) solution, followed by the addition of 300 μl HPLC-grade water and vortex for 1 min. The homogenate was kept on ice for 15 min; samples were centrifuged for 15 min at 14,000 ×g (4° C), the aqueous and organic phases were collected into separate clean glass vials and the extraction step was repeated. The organic solvents and the residual water were removed from the samples using a speed vacuum concentrator. Dried aqueous extracts were reconstituted in 600 μl buffer containing 0.2 M Na_2HPO_4, 0.043 M NaH_2PO_4, 1 mM TSP, 3 mM NaN_3, 100% D_2O, pH 7.4 (not corrected for isotope effects), while the organic extract was reconstituted in 650 μl of $CDCl_3$ containing 0.03% (v/v) TMS; 550 μl of the samples were pipetted in 5 mm diameter NMR tubes. ^1H 1D NMR spectra of the polar metabolites collected by the above protocol showed a flat baseline, a feature that facilitates the further spectroscopic data comparison using statistical analysis.

NMR spectra were recorded on a Bruker (Karlsruhe, Germany) AVANCE III spectrometer (operating at 600.11 MHz for ^1H nucleus), equipped with a z-gradient inverse detection 5 mm probe, at 300.0 K. Sample loading, field homogeneity optimization, 90-degree pulse calibration, acquisition and processing including Fourier transformation, phase correction, and axis calibration were fully automated using ICON-NMR v. 4.2.6. software controlling a 60 position-Bruker Autosampler (B-ACS). Noesypr1D pulse sequence as implemented in Bruker library (noesygppr1d) was used for sample profiling and a J-resolved spectrum (jresgpprqf; Bruker library) was also recorded for the elucidation of the unresolved peaks due to overlapping. For the ^1H 1D spectra 192 scans were acquired with 64k points analysis each for a spectral width of 12019 Hz. The acquisition period was 2.72 s, the presaturation power adjusted at 25 Hz, and the mixing time at 0.010 s. The J-resolved spectra were recorded with an analysis of 8k points over a 10000 Hz spectral width, 4 scans and 40 transients in F1 dimension. For selected samples 2D homonuclear (TOCSY) and heteronuclear (HSQC-DEPT 135) experiments were recorded to facilitate the signal assignment. TOCSY experiments were recorded with 4k data points for a 9615 Hz spectral width, 128 scans and 380 transients in F1 dimension, while for HSQC-DEPT 135 spectra 1k x 200 data points for 9615 and 27163 Hz spectral widths for F2 and F1 dimensions, respectively were acquired with 180 scans. Spectra were processed with TopSpin v. 3.1 software (Bruker BioSpin).

Statistical analysis of NMR-based metabolomics

For NMR-based metabolomics statistical analysis all 1D ^1H NMR spectra were reduced to a series of descriptors using AMIX Statistics v. 3.9.14 software (Bruker Biospin). Simple rectangular buckets of 0.02 ppm width were generated from the ^1H 1D NMR spectra for the spectral area 9.40 to 0.80 ppm. After the exclusion of the residual solvent signal region (4.90-4.68 ppm) the spectral area was normalized to total intensity. Furthermore, a data set of 0.005 ppm-width buckets was used in order to analyze the variation of the identified metabolites. The generated data sets were *pareto* scaled by dividing each variable by the square root of the standard deviation (SD) and subjected to multivariate analysis using SIMCA-P^+ v. 11.5 (Umetrics AB, Sweden) software. Principal component analysis (PCA) was used to visualize the non-supervised clustering of samples, while Partial Least Squares-Discriminant Analysis (PLS-DA) and orthogonal PLS-DA (oPLS-DA) were used to reveal the most affected metabolites in experimental *vs.* control samples. The quality of the generated PLS-DA models was verified through with permutation test for 100 random permutations. The predictive capacity of the generated models was evaluated by Q2 cumulative index. The loadings plot of the PLS-DA models, the S-plots of the oPLS-DA, as well as the Variable Importance in Projection (VIP) values were used to identify the characteristic metabolites of each experimental group. Spectroscopic data were also subjected to univariate analysis tools, as t-test and one-way analysis of variance (ANOVA) with Bonferroni correction and with Tukey post-hoc analysis, for the investigation of statistical significant changes between each pair of control *vs.* experimental groups. Deviations from the

corresponding control samples were depicted using z-score values using the formula ($mean_{group}$-$mean_{control}$)/$SD_{control}$ in combination with the related t-test results.

Antibodies used

The following antibodies were kind gifts. The polyclonal antibody against the *Drosophila* β5 proteasome subunit was from Prof. M. Figueiredo-Pereira (Hunter College, New York, USA); the antibody against Atg8α was from Prof K. Koehler (ETH Zurich, Switzerland); the anti-α-GLY antibody was from Prof O. Babba (Ohu University, Japan); the antibodies against dIlp2 and Imp-L2 were from Prof. Ernst Hafen (ETH Zurich, Switzerland), and the anti-Ref(2)P antibody was from Gábor Juhász (Eotvos Lorand University, Hungary). Additional antibodies are described in Key Resources Tables.

Statistical analyses

Experiments were performed at least in duplicates (for each biological replicate, $n \geq 2$). Assays were done after pooling isolated male/female somatic tissues from 10-20 flies, unless otherwise stated. For statistical analysis, the MS Excel and the Statistical Package for Social Sciences (IBM SPSS; version 19.0 for Windows, NY, USA) were used. Statistical significance was evaluated using one-way analysis of variance (ANOVA). Data points correspond to the mean of the independent experiments and error bars denote standard deviation (SD); significance at $P<0.05$ or $P<0.01$ is indicated in graphs by one or two asterisks, respectively. For LC-MS experiments (n=3) differences in Log_2 ratio values at $P<0.05$ were accepted (see also above).

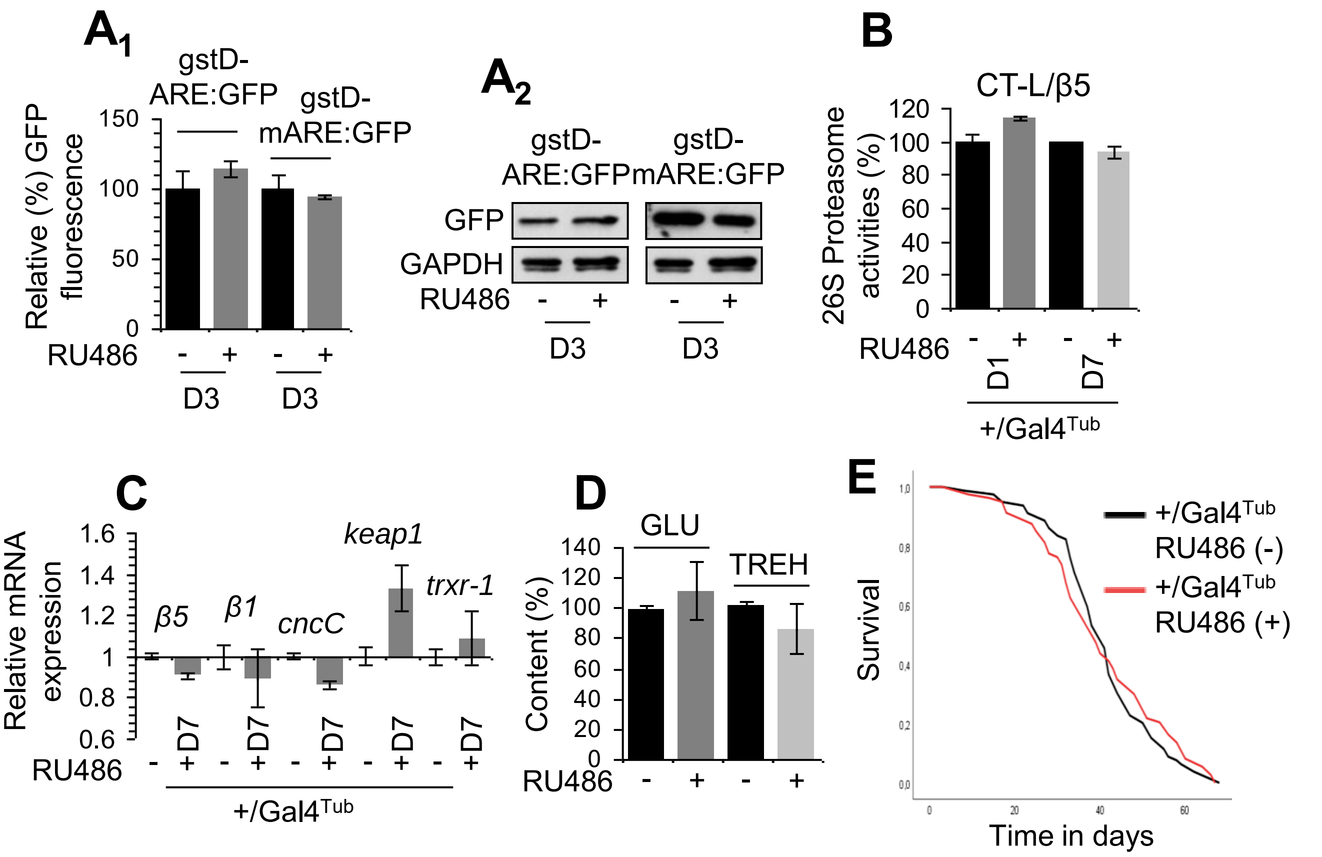
A1
gstD-ARE:GFP
gstD-mARE:GFP
Relative (%) GFP fluorescence
150
100
50
0
RU486
- + - +
D3 D3
A2
gstD-ARE:GFP
gstD-mARE:GFP
GFP
GAPDH
RU486
- + - +
D3 D3
B
CT-L/β5
26S Proteasome activities (%)
120
100
80
60
40
20
0
RU486
- + - +
D1 D7
+/Gal4Tub
C
Relative mRNA expression
1.6
1.4
1.2
1
0.8
0.6
β5
β1
cncC
keap1
trxr-1
D7 D7 D7 D7 D7
RU486
- + - + - + - + - +
+/Gal4Tub
D
GLU
TREH
Content (%)
140
120
100
80
60
40
20
0
RU486
- + - +
E
Survival
+/Gal4Tub RU486 (-)
+/Gal4Tub RU486 (+)
Time in days

Figure S1

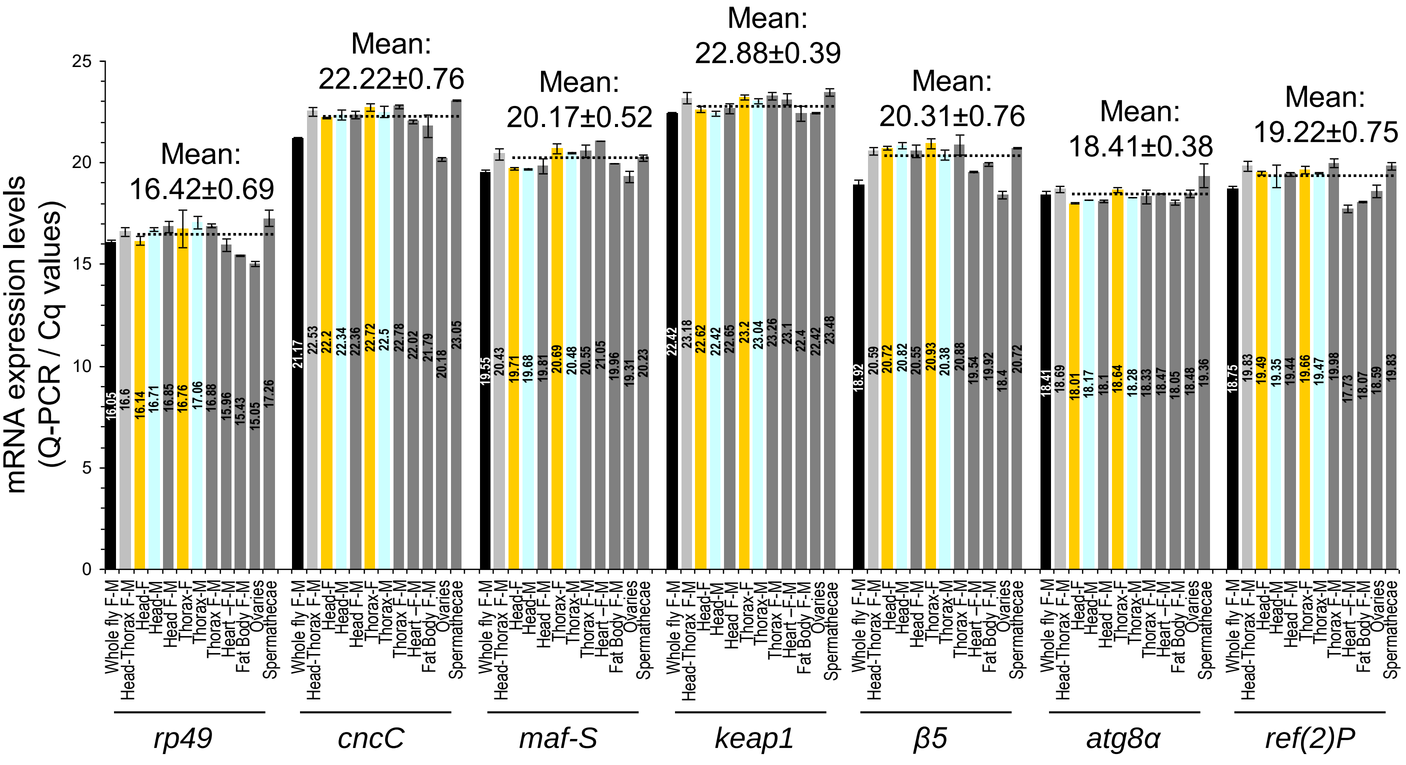
mRNA expression levels
(Q-PCR / Cq values)
0
5
10
15
20
25
Mean: 16.42±0.69
Mean: 22.22±0.76
Mean: 20.17±0.52
Mean: 22.88±0.39
Mean: 20.31±0.76
Mean: 18.41±0.38
Mean: 19.22±0.75
16.05
16.6
16.14
16.71
16.85
16.76
17.06
16.88
15.96
15.43
15.05
17.26
21.17
22.53
22.2
22.34
22.36
22.72
22.5
22.78
22.02
21.79
20.18
23.05
19.55
20.43
19.71
19.68
19.81
20.69
20.48
20.55
21.05
19.96
19.31
20.23
22.42
23.18
22.62
22.42
22.65
23.2
23.04
23.26
23.1
22.4
22.42
23.48
18.92
20.59
20.72
20.82
20.55
20.93
20.38
20.88
19.54
19.92
18.4
20.72
18.41
18.69
18.01
18.17
18.1
18.64
18.28
18.33
18.47
18.05
18.48
19.36
18.75
19.83
19.49
19.35
19.44
19.66
19.47
19.98
17.73
18.07
18.59
19.83
Whole fly F-M
Head-Thorax F-M
Head-F
Head-M
Head F-M
Thorax-F
Thorax-M
Thorax F-M
Heart –F-M
Fat Body F-M
Ovaries
Spermathecae
rp49
cncC
maf-S
keap1
β5
atg8α
ref(2)P

Figure S2

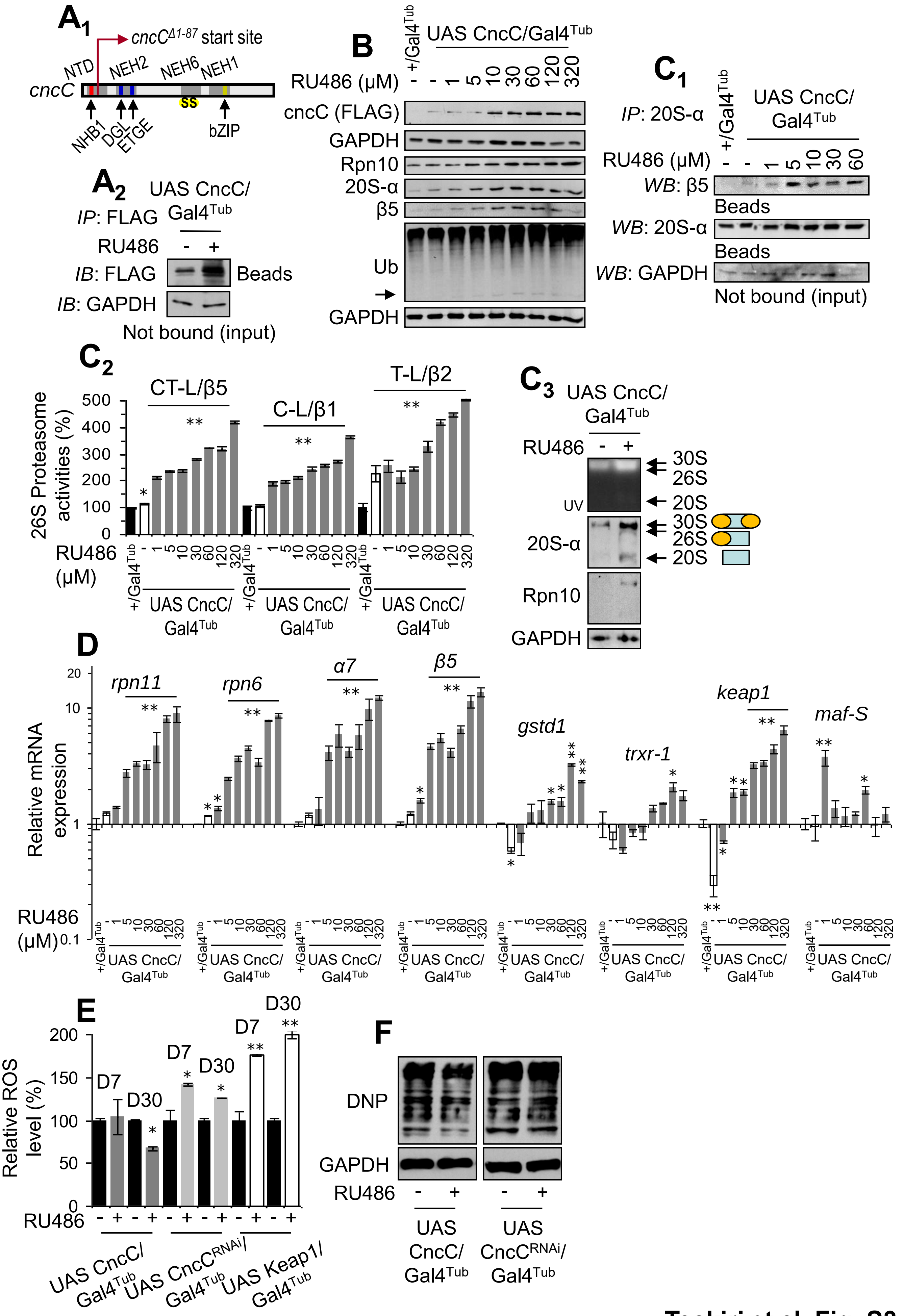

Tsakiri et al. Fig. S3

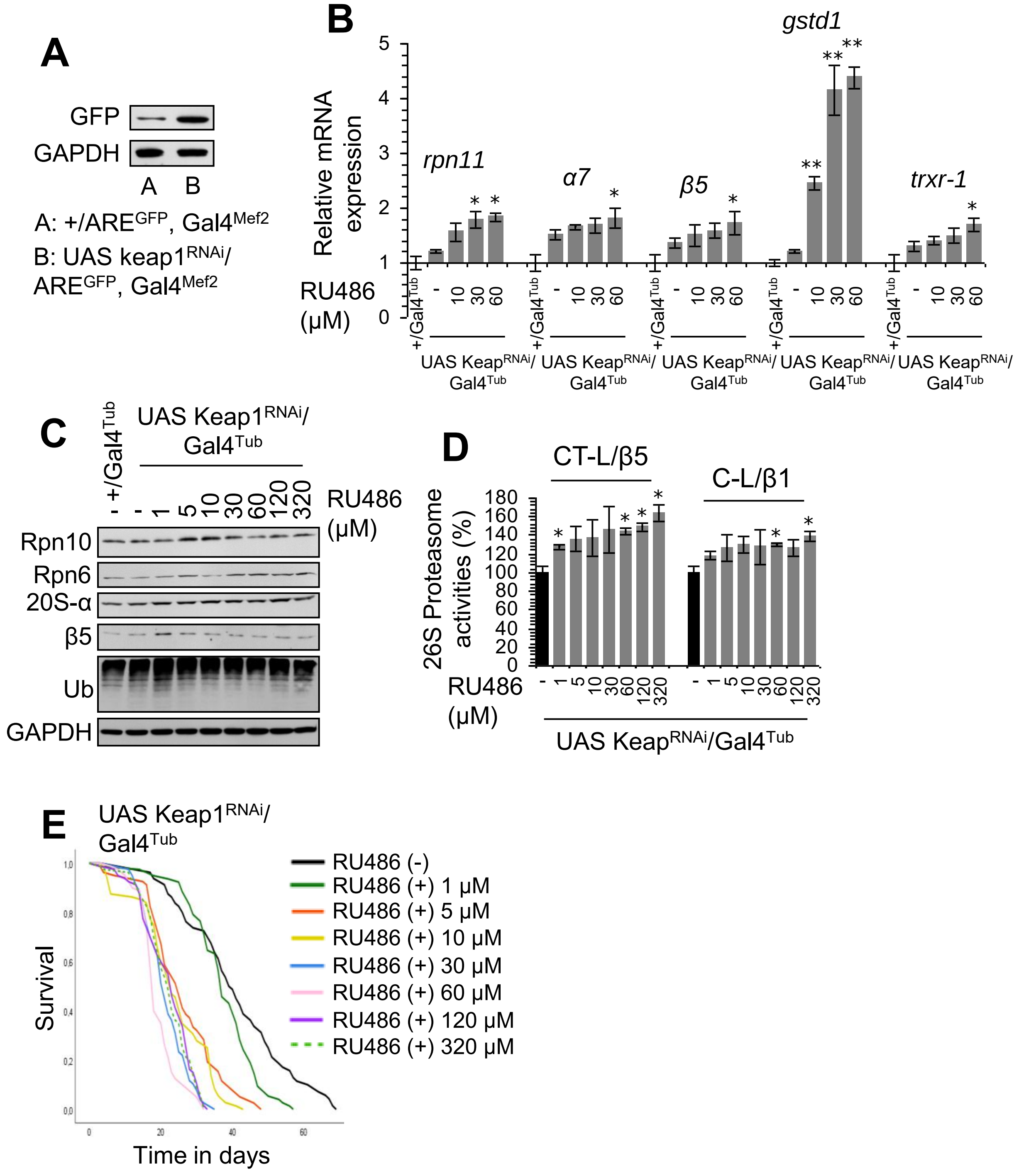

A
GFP
GAPDH
A B
A: +/ARE^GFP, Gal4^Mef2
B: UAS keap1^RNAi/ ARE^GFP, Gal4^Mef2
B
Relative mRNA expression
rpn11
α7
β5
gstd1
trxr-1
RU486 (μM)
+/Gal4^Tub
- 10 30 60
UAS Keap^RNAi/ Gal4^Tub
C
+/Gal4^Tub
UAS Keap1^RNAi/ Gal4^Tub
- - 1 5 10 30 60 120 320
RU486 (μM)
Rpn10
Rpn6
20S-α
β5
Ub
GAPDH
D
CT-L/β5
C-L/β1
26S Proteasome activities (%)
RU486 (μM)
- 1 5 10 30 60 120 320
UAS Keap^RNAi/Gal4^Tub
E
UAS Keap1^RNAi/ Gal4^Tub
RU486 (-)
RU486 (+) 1 μM
RU486 (+) 5 μM
RU486 (+) 10 μM
RU486 (+) 30 μM
RU486 (+) 60 μM
RU486 (+) 120 μM
RU486 (+) 320 μM
Survival
Time in days

Figure S4

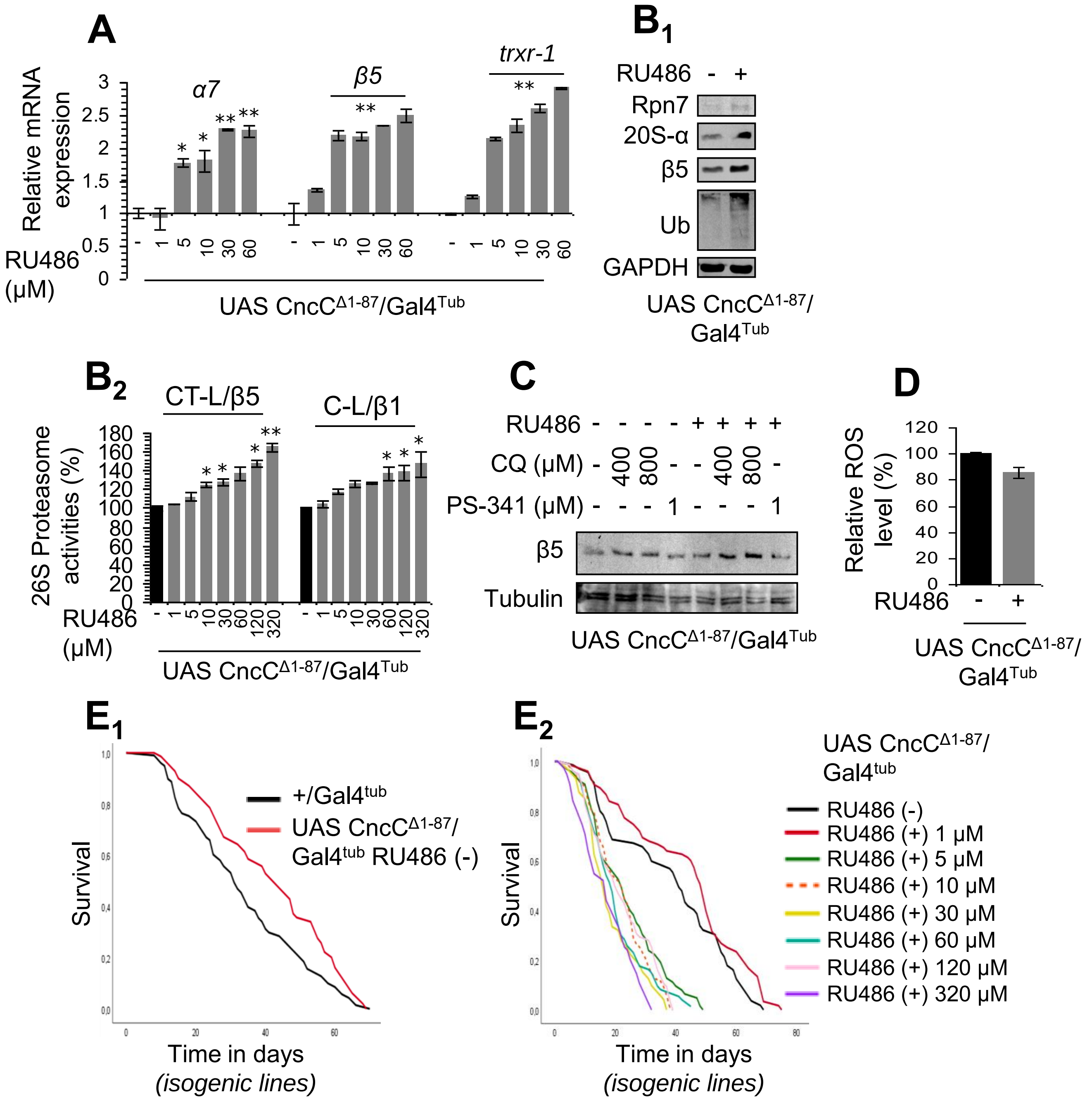

A
Relative mRNA expression
α7
β5
trxr-1
RU486 (μM)
UAS CncCΔ1-87/Gal4Tub
B1
RU486 - +
Rpn7
20S-α
β5
Ub
GAPDH
UAS CncCΔ1-87/Gal4Tub
B2
CT-L/β5
C-L/β1
26S Proteasome activities (%)
RU486 (μM)
UAS CncCΔ1-87/Gal4Tub
C
RU486 - - - - + + + +
CQ (μM) - 400 800 - - 400 800 -
PS-341 (μM) - - - 1 - - - 1
β5
Tubulin
UAS CncCΔ1-87/Gal4Tub
D
Relative ROS level (%)
RU486 - +
UAS CncCΔ1-87/Gal4Tub
E1
+/Gal4tub
UAS CncCΔ1-87/Gal4tub RU486 (-)
Survival
Time in days (isogenic lines)
E2
UAS CncCΔ1-87/Gal4tub
RU486 (-)
RU486 (+) 1 μM
RU486 (+) 5 μM
RU486 (+) 10 μM
RU486 (+) 30 μM
RU486 (+) 60 μM
RU486 (+) 120 μM
RU486 (+) 320 μM
Survival
Time in days (isogenic lines)

Figure S5

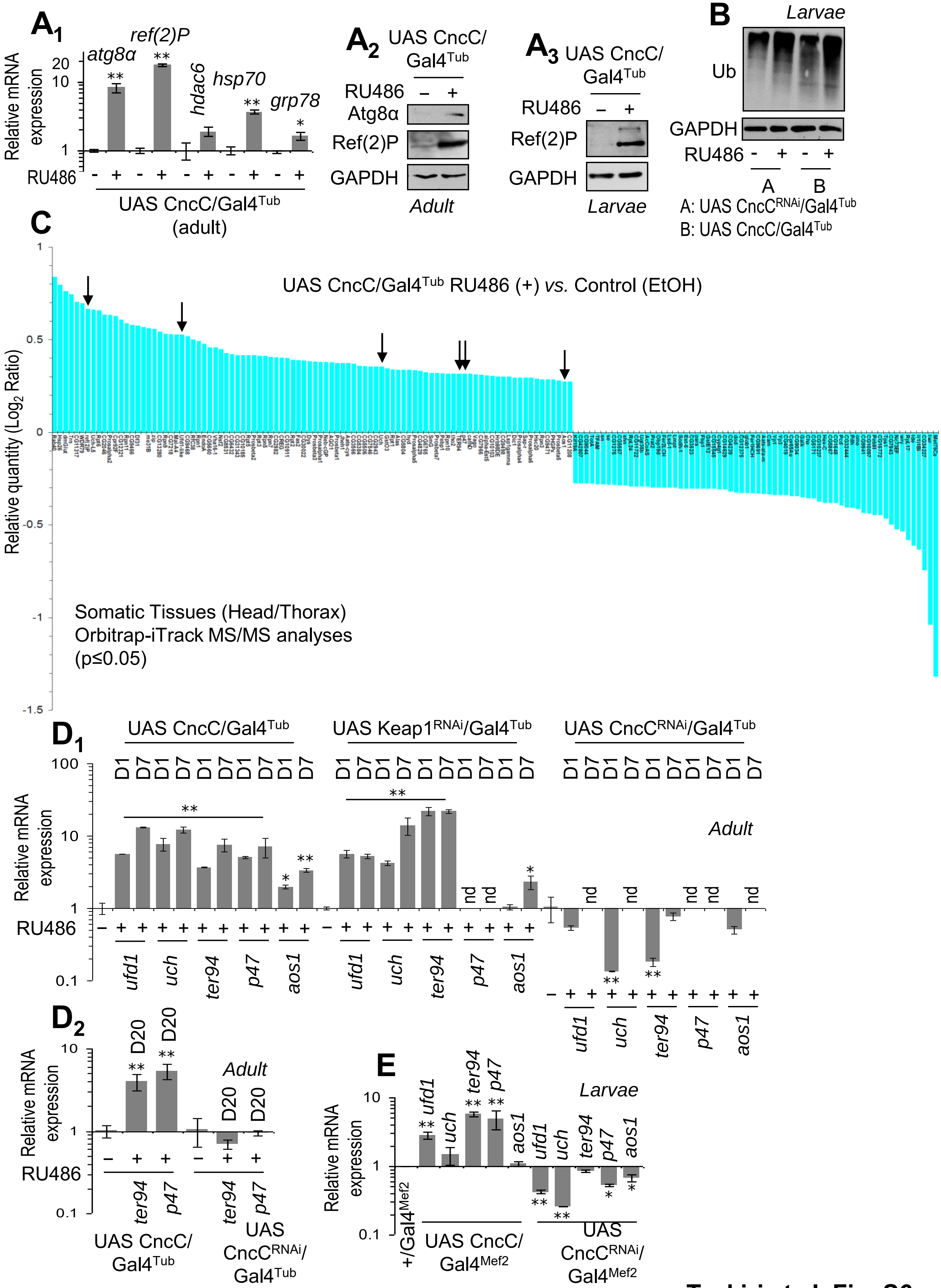

Tsakiri et al. Fig. S6

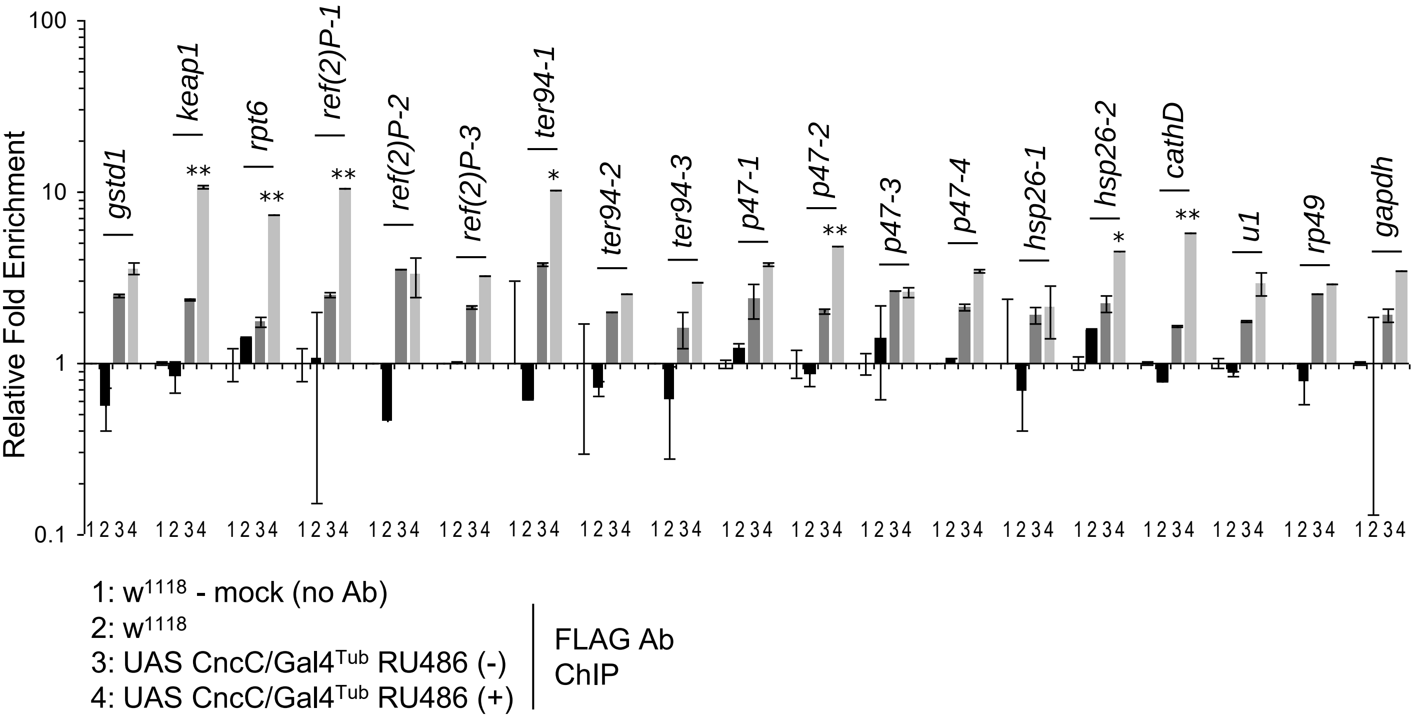
Relative Fold Enrichment
100
10
1
0.1
gstd1
keap1
rpt6
ref(2)P-1
ref(2)P-2
ref(2)P-3
ter94-1
ter94-2
ter94-3
p47-1
p47-2
p47-3
p47-4
hsp26-1
hsp26-2
cathD
u1
rp49
gapdh
**
**
**
*
**
*
**
1 2 3 4
1: w1118 - mock (no Ab)
2: w1118
3: UAS CncC/Gal4Tub RU486 (-)
4: UAS CncC/Gal4Tub RU486 (+)
FLAG Ab ChIP

Figure S7

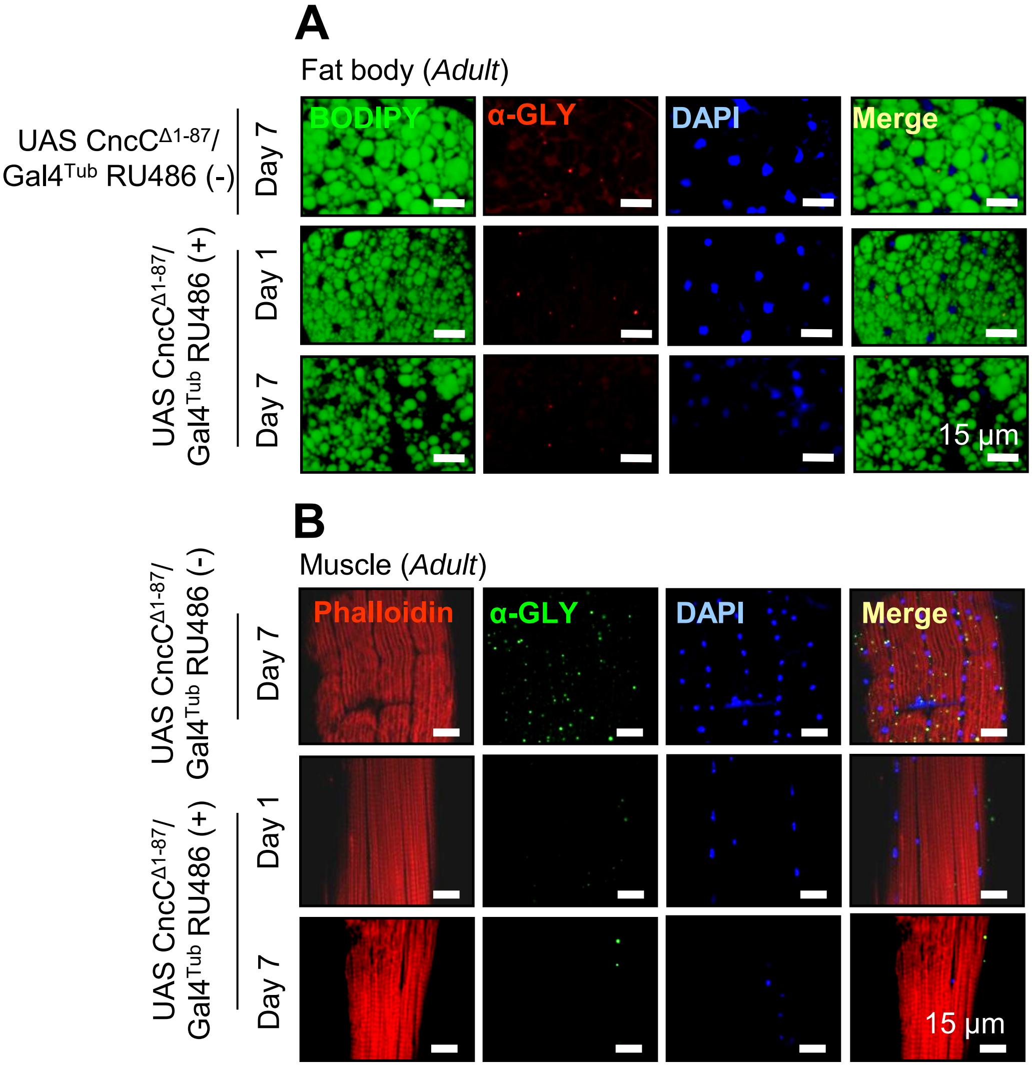
A
Fat body (Adult)
UAS CncCΔ1-87/
Gal4Tub RU486 (-)
Day 7
BODIPY
α-GLY
DAPI
Merge
UAS CncCΔ1-87/
Gal4Tub RU486 (+)
Day 1
Day 7
15 µm
B
Muscle (Adult)
UAS CncCΔ1-87/
Gal4Tub RU486 (-)
Day 7
Phalloidin
α-GLY
DAPI
Merge
UAS CncCΔ1-87/
Gal4Tub RU486 (+)
Day 1
Day 7
15 µm

Figure S8

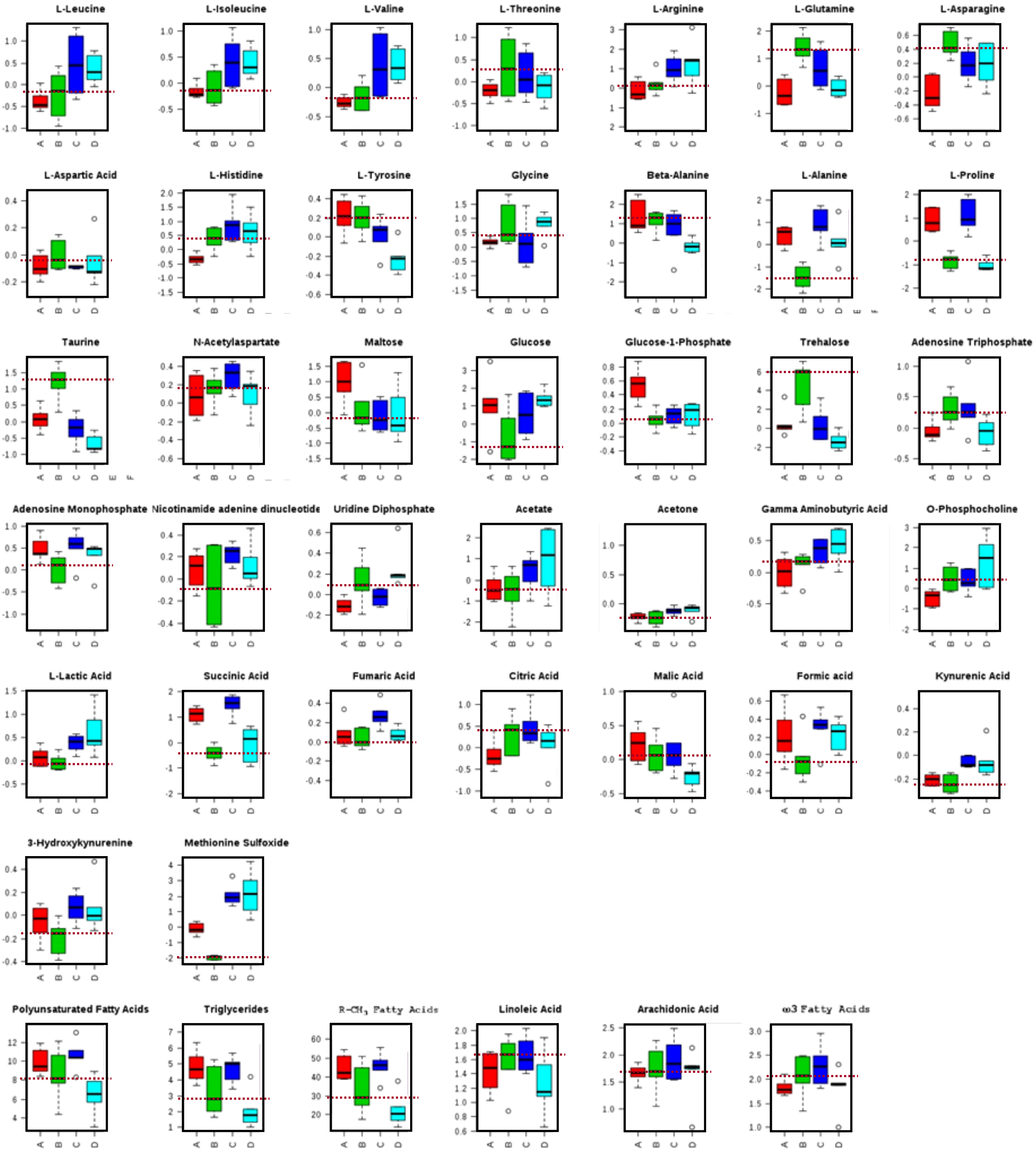

A: UAS CncC/Gal4^{Tub} RU486 (-); B: UAS CncC/Gal4^{Tub} RU486 (+)

C: UAS CncC^{RNAi}/Gal4^{Tub} RU486 (-); D: UAS CncC^{RNAi}/Gal4^{Tub} RU486 (+)

Tsakiri et al. Fig. S9

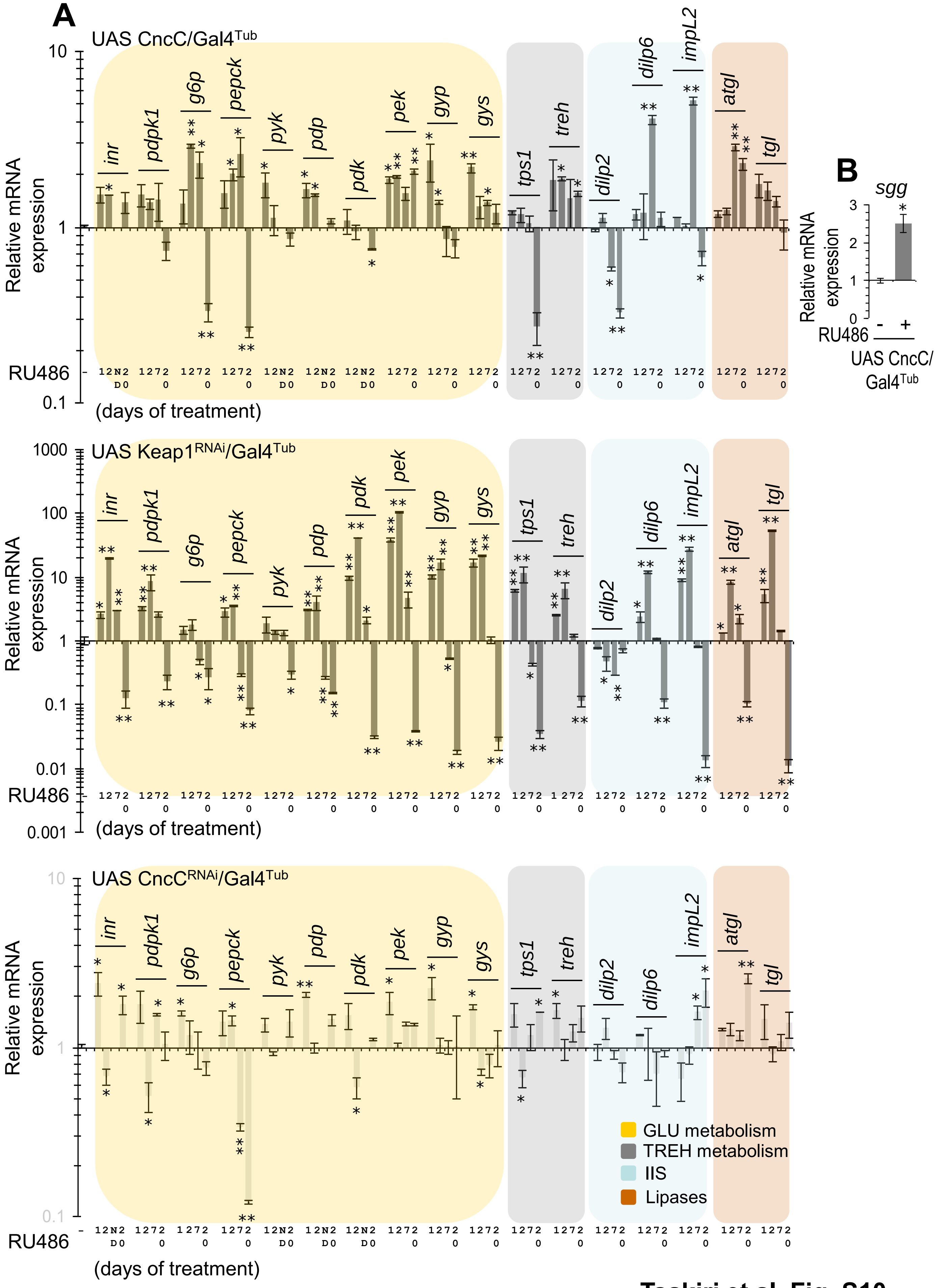

Tsakiri et al. Fig. S10

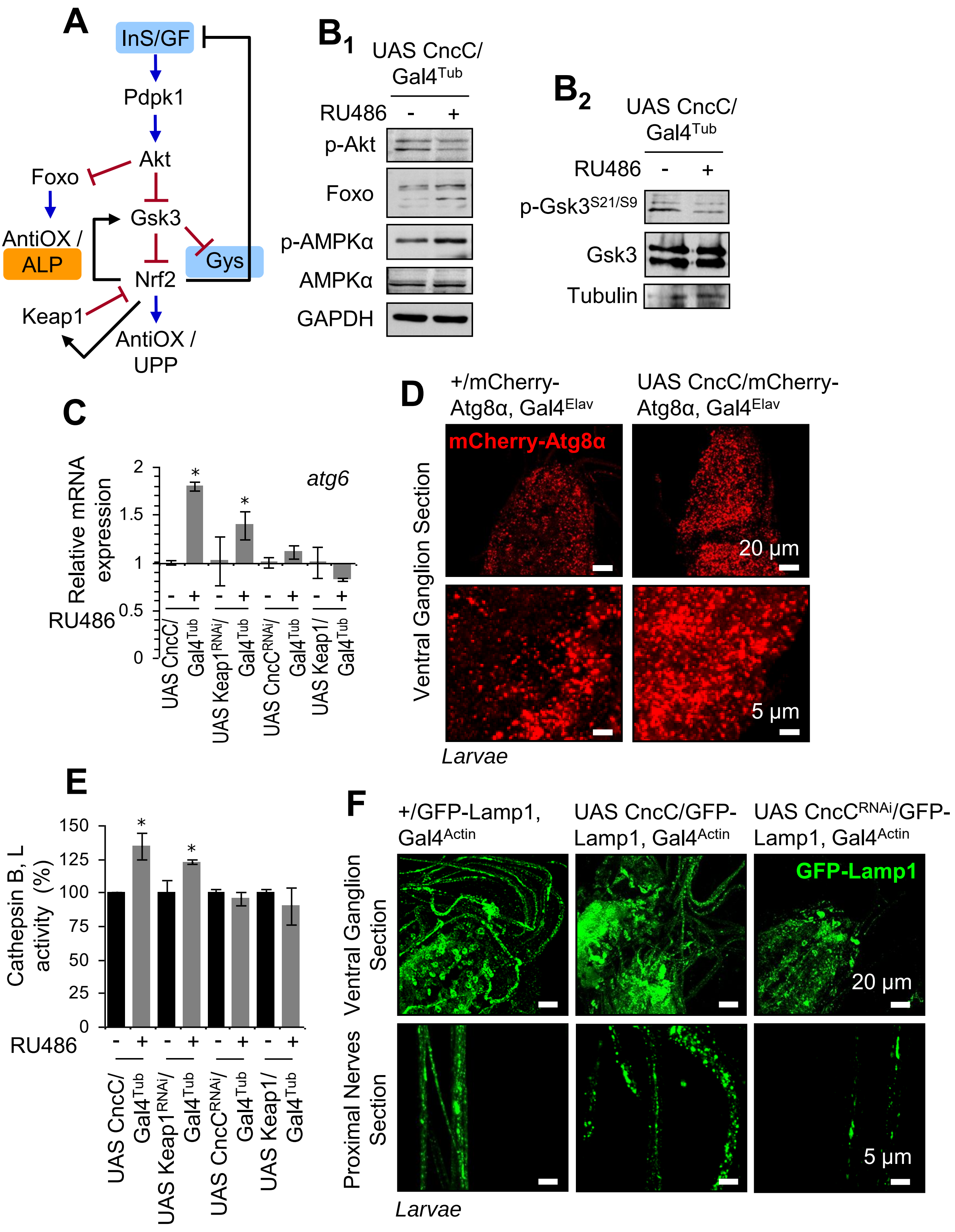

Tsakiri et al. Fig. S11

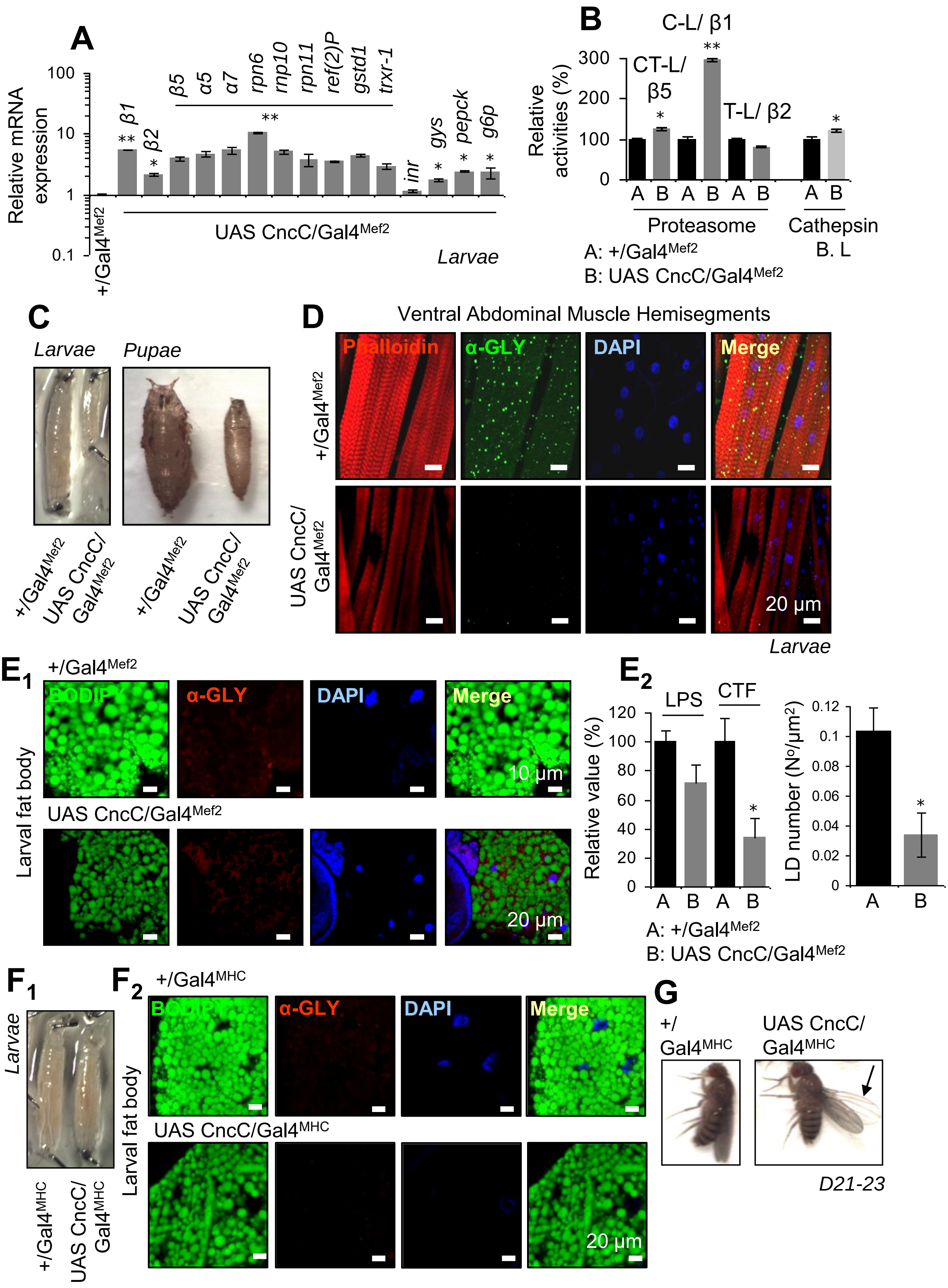

Tsakiri et al. Fig. S12

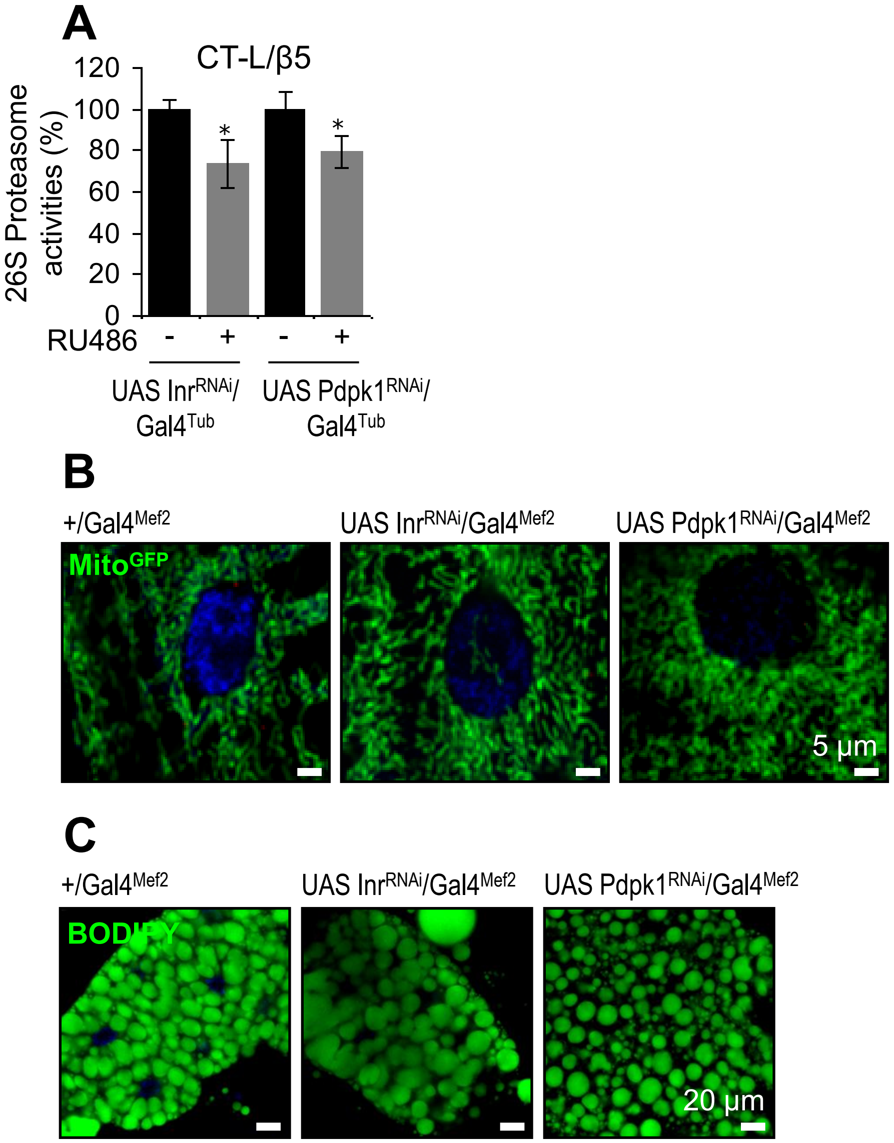

Tsakiri et al. Fig. S13

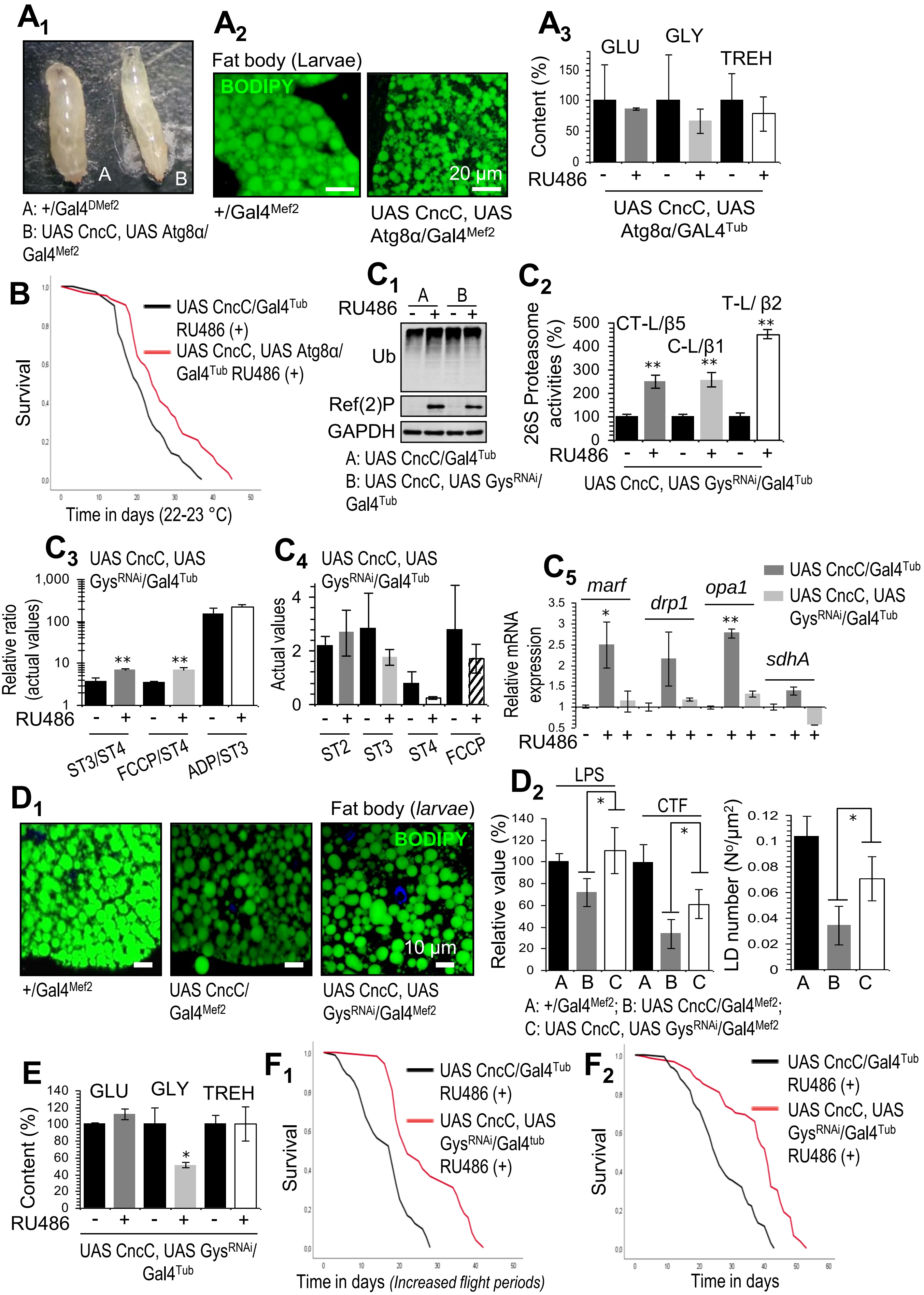

Tsakiri et al. Fig. S14

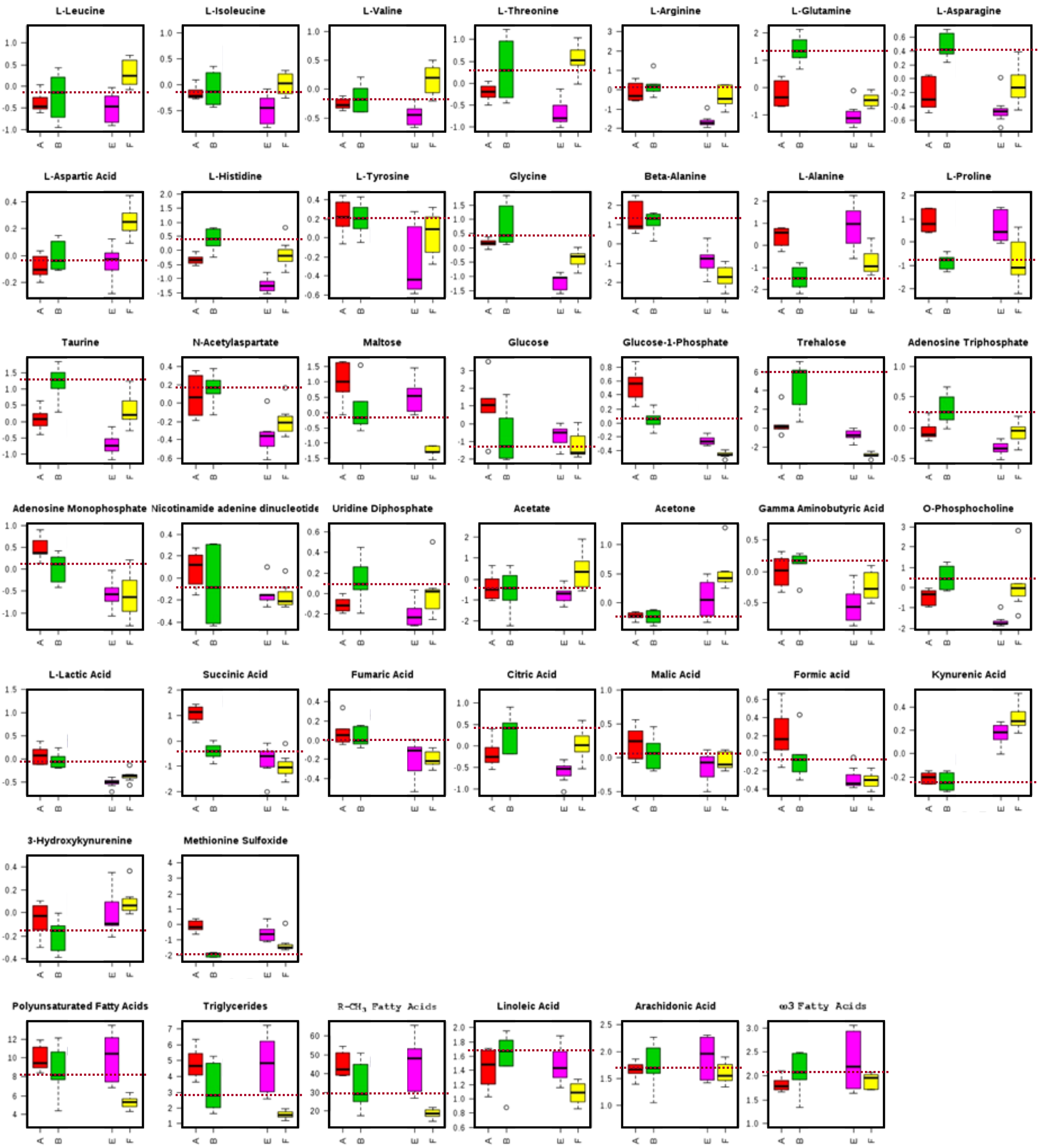

A: UAS CncC/Gal4^{Tub} RU486 (-)*

B: UAS CncC/Gal4^{Tub} RU486 (+)*

E: UAS CncC, UAS Gys^{RNAi}/Gal4^{Tub} RU486 (-)

F: UAS CncC, UAS Gys^{RNAi}/Gal4^{Tub} RU486 (+)

** Data also shown in Fig. S9*

Figure S15

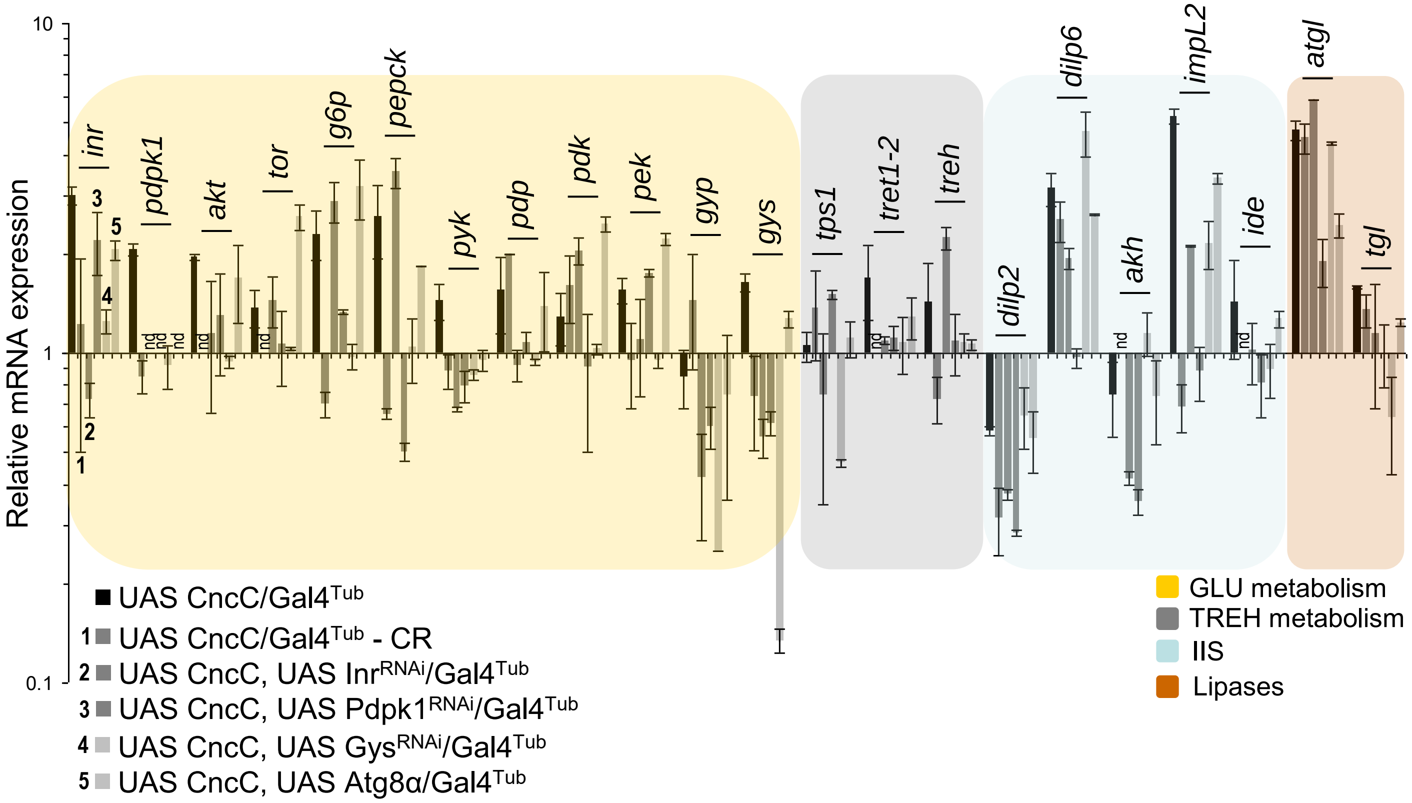

Relative mRNA expression
10
1
0.1
inr
pdpk1
akt
tor
g6p
pepck
pyk
pdp
pdk
pek
gyp
gys
tps1
tret1-2
treh
dilp2
dilp6
akh
impL2
ide
atgl
tgl
nd
UAS CncC/Gal4Tub
1 UAS CncC/Gal4Tub - CR
2 UAS CncC, UAS InrRNAi/Gal4Tub
3 UAS CncC, UAS Pdpk1RNAi/Gal4Tub
4 UAS CncC, UAS GysRNAi/Gal4Tub
5 UAS CncC, UAS Atg8α/Gal4Tub
GLU metabolism
TREH metabolism
IIS
Lipases

Figure S16

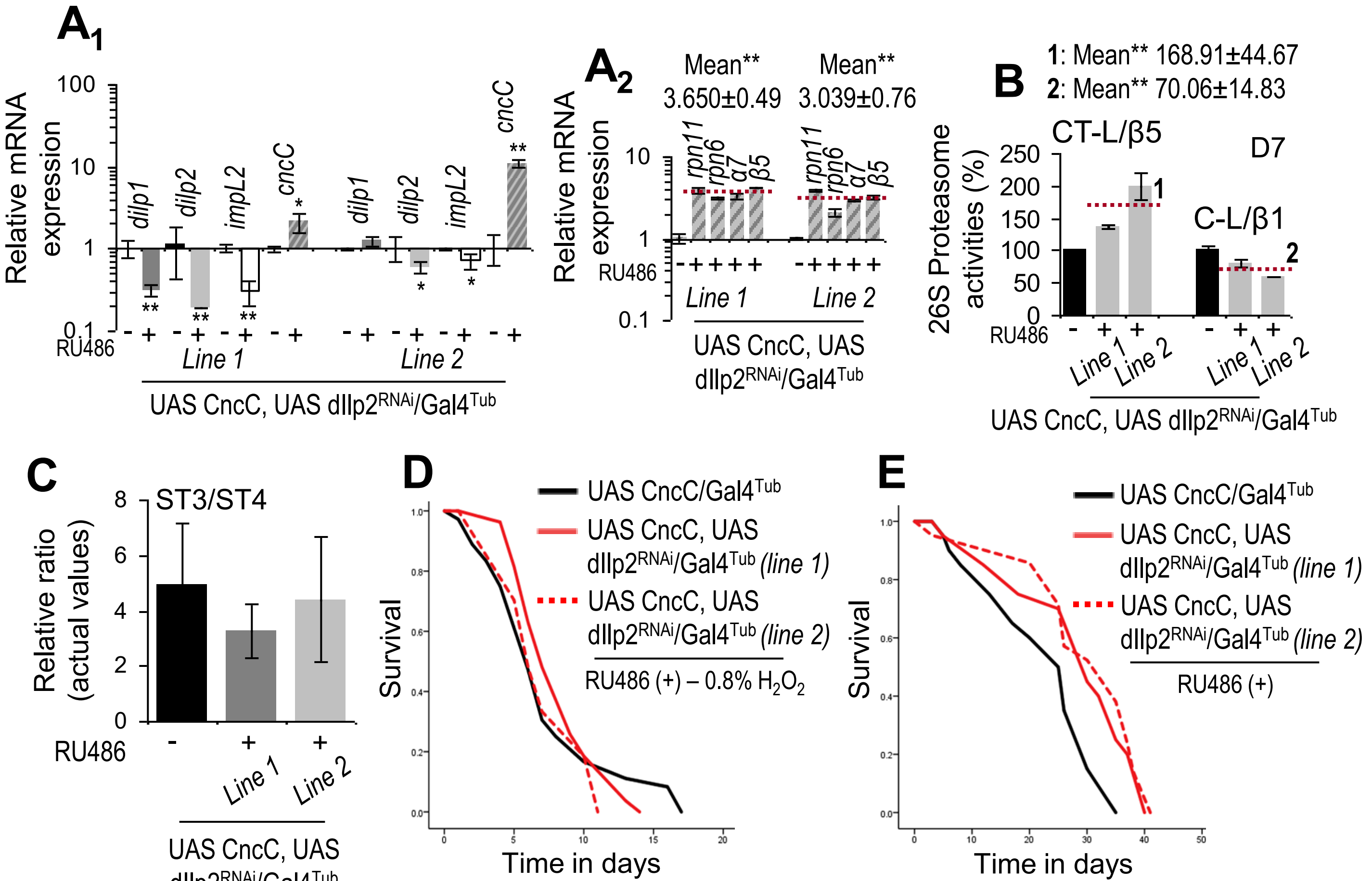

Tsakiri et al. Fig. S17

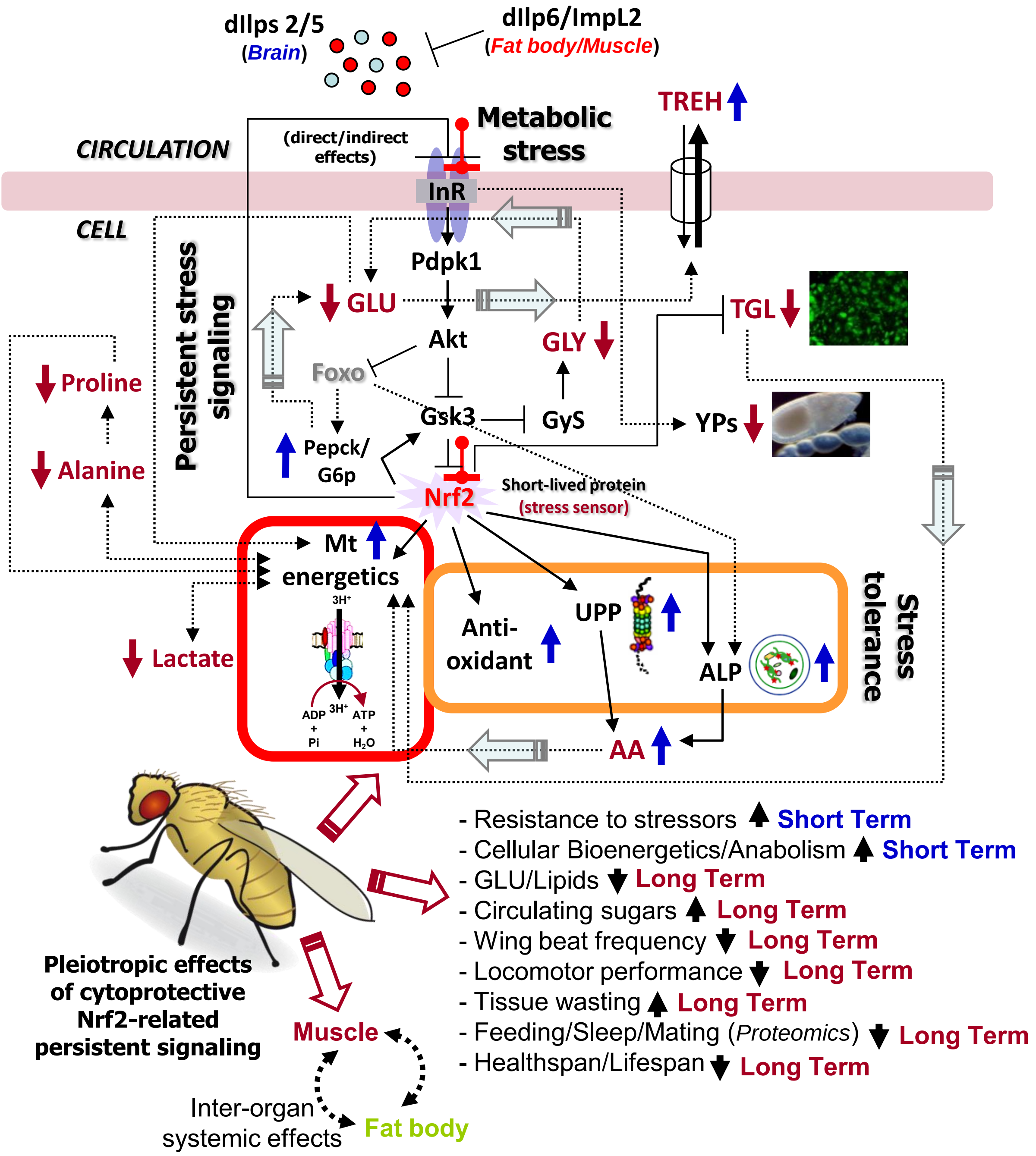

Tsakiri et al. Fig. S18

Supporting Table S1. Summary of lifespan experiments.

Figure	Sample	Mean Lifespan (LF) +/- s.e.m. (Days)		Median Lifespan +/- s.e.m. (Days)		% Median LF vs. control	Max (Days)	Log Rank P Value: +/Gal4^{Tub} RU486(-)	+/Gal4^{Tub} RU486(+)	Total Animals Died/Total
Fig. S1E	+/Gal4^{Tub} RU486(-)	40.443	1.337	40	1.234	100	68		0.669	79/80
	+/Gal4^{Tub} RU486(+)	40.132	1.620	38	1.988	95	67	0.669		78/80

Figure	Sample	Mean LF	s.e.m.	Median LF	s.e.m.	% Median LF vs. control	Max (Days)	+/Gal4^{Tub} RU486(-)	UAS CncC/Gal4^{Tub} RU486(-)	*Total Animals Died/Total*
Fig. 1D	+/Gal4^{Tub} RU486(-)	36.181	1.462	36	2.178	100	69		0.000	79/101
	UAS CncC/Gal4^{Tub} RU486(-)	50.051	1.778	54	3.385	150	72	0.000		79/87

Figure	Sample	Mean LF	s.e.m.	Median LF	s.e.m.	% Median LF vs. control	Max (Days)	UAS CncC/Gal4Tub RU486(-)	UAS CncC/Gal4Tub RU486 1 μM	UAS CncC/Gal4^{Tub} RU486 5 μM	UAS CncC/Gal4^{Tub} RU486 10 μM	UAS CncC/Gal4^{Tub} RU486 30 μM	UAS CncC/Gal4^{Tub} RU486 60 μM	UAS CncC/Gal4^{Tub} RU486 120 μM	UAS CncC/Gal4^{Tub} RU486 320 μM	*Total Animals Died/Total*
Fig. 1E$_1$	UAS CncC/Gal4^{Tub} RU486(-)	43.286	2.047	46	2.566	100	65		0.000	0.000	0.000	0.000	0.000	0.000	0.000	54/62
	UAS CncC/Gal4^{Tub} RU486 1 μM	35.647	1.458	36	3.103	78	54	0.000		0.000	0.000	0.000	0.000	0.000	0.000	73/74
	UAS CncC/Gal4^{Tub} RU486 5 μM	25.893	0.854	24	1.139	52	44	0.000	0.000		0.209	0.000	0.154	0.002	0.000	75/76
	UAS CncC/Gal4^{Tub} RU486 10 μM	23.193	1.072	22	1.139	47	38	0.000	0.000	0.209		0.000	0.853	0.074	0.001	69/74
	UAS CncC/Gal4^{Tub} RU486 30 μM	17.419	0.794	16	0.485	34	34	0.000	0.000	0.000	0.000		0.000	0.001	0.031	70/74
	UAS CncC/Gal4^{Tub} RU486 60 μM	22.933	1.114	22	2.886	47	42	0.000	0.000	0.154	0.853	0.000		0.107	0.001	75/76
	UAS CncC/Gal4^{Tub} RU486 120 μM	21.471	0.815	20	0.712	43	38	0.000	0.000	0.002	0.074	0.001	0.107		0.096	87/87
	UAS CncC/Gal4^{Tub} RU486 320 μM	20.962	0.593	22	0.842	47	32	0.000	0.000	0.000	0.001	0.031	0.001	0.096		80/81

Figure	Sample	Mean LF	s.e.m.	Median LF	s.e.m.	% Median LF vs. control	Max (Days)	UAS CncC/Gal4^{Tub} RU486(-)	UAS CncC/Gal4^{Tub} RU486(+) - *for 2-3 days every 10 days*	*Total Animals Died/Total*
Fig. 1F	UAS CncC/Gal4^{Tub} RU486(-)	54.638	2.443	53	3.265	100	74.0		0.000	30/35
	UAS CncC/Gal4^{Tub} RU486(+) - *for 2-3 days every 10 days*	37.506	1.079	39	1.212	73	46.0	0.000		26/33

Figure	Sample	Mean LF	s.e.m.	Median LF	s.e.m.	% Median LF vs. control	Max (Days)	UAS Keap1^{RNAi}/Gal4^{Tub} RU486(-)	UAS Keap1^{RNAi}/Gal4^{Tub} RU486 1 μM	UAS Keap1^{RNAi}/Gal4^{Tub} RU486 5 μM	UAS Keap1^{RNAi}/Gal4^{Tub} RU486 10 μM	UAS Keap1^{RNAi}/Gal4^{Tub} RU486 30 μM	UAS Keap1^{RNAi}/Gal4^{Tub} RU486 60 μM	UAS Keap1^{RNAi}/Gal4^{Tub} RU486 120 μM	UAS Keap1^{RNAi}/Gal4^{Tub} RU486 320 μM	Total Animals Died/Total
Fig. S4E	UAS Keap1^{RNAi}/Gal4^{Tub} RU486(-)	40.703	1.592	39	1.987	100	69		0.004	0.000	0.000	0.000	0.000	0.000	0.000	78/80
	UAS Keap1^{RNAi}/Gal4^{Tub} RU486 1 μM	37.071	1.093	37	1.280	95	57	0.004		0.000	0.000	0.000	0.000	0.000	0.000	75/80
	UAS Keap1^{RNAi}/Gal4^{Tub} RU486 5 μM	25.867	1.099	25	1.472	64	48	0.000	0.000		0.147	0.000	0.000	0.001	0.000	79/80
	UAS Keap1^{RNAi}/Gal4^{Tub} RU486 10 μM	23.487	1.104	23	1.347	59	43	0.000	0.000	0.147		0.002	0.000	0.012	0.003	76/80
	UAS Keap1^{RNAi}/Gal4^{Tub} RU486 30 μM	21.383	0.651	21	0.635	54	35	0.000	0.000	0.000	0.002		0.016	0.264	0.441	79/80
	UAS Keap1^{RNAi}/Gal4^{Tub} RU486 60 μM	19.062	0.593	18	0.312	46	32	0.000	0.000	0.000	0.000	0.016		0.000	0.001	80/80
	UAS Keap1^{RNAi}/Gal4^{Tub} RU486 120 μM	22.24	0.754	23	0.81	59	33	0.000	0.000	0.001	0.012	0.264	0.000		0.615	78/80
	UAS Keap1^{RNAi}/Gal4^{Tub} RU486 320 μM	21.95	0.715	22	0.958	56	32	0.000	0.000	0.000	0.003	0.441	0.001	0.615		80/80

Figure	Sample	Mean LF	s.e.m.	Median LF	s.e.m.	% Median LF vs. control	Max (Days)	+/Gal4^{Tub} RU486(-)	UAS CncC$^{\Delta1-87}$/Gal4^{Tub} RU486(-)	Total Animals Died/Total
Fig. S5E$_1$	+/Gal4^{Tub} RU486(-)	33.545	1.815	31	2.413	100	70		0.021	82/100
	UAS CncC$^{\Delta1-87}$/Gal4^{Tub} RU486(-)	41.271	2.100	42	3.660	135	69	0.021		64/70

Figure	Sample	Mean LF	s.e.m.	Median LF	s.e.m.	% Median LF vs. control	Max (Days)	UAS CncC$^{\Delta1-87}$/Gal4^{Tub} RU486(-)	UAS CncC$^{\Delta1-87}$/Gal4^{Tub} RU486 1 μM	UAS CncC$^{\Delta1-87}$/Gal4^{Tub} RU486 5 μM	UAS CncC$^{\Delta1-87}$/Gal4^{Tub} RU486 10 μM	UAS CncC$^{\Delta1-87}$/Gal4^{Tub} RU486 30 μM	UAS CncC$^{\Delta1-87}$/Gal4^{Tub} RU486 60 μM	UAS CncC$^{\Delta1-87}$/Gal4^{Tub} RU486 120 μM	UAS CncC$^{\Delta1-87}$/Gal4^{Tub} RU486 320 μM	Total Animals Died/Total
Fig. S5E$_2$	UAS CncC$^{\Delta1-87}$/Gal4^{Tub} RU486(-)	38.293	2.277	41	2.016	100	69		0.046	0.000	0.000	0.000	0.000	0.000	0.000	64/70
	UAS CncC$^{\Delta1-87}$/Gal4^{Tub} RU486 1 μM	43.735	2.237	49	1.308	119	75	0.046		0.000	0.000	0.000	0.000	0.000	0.000	70/76
	UAS CncC$^{\Delta1-87}$/Gal4^{Tub} RU486 5 μM	23.436	1.494	22	2.260	53	49	0.000	0.000		0.106	0.003	0.064	0.194	0.000	63/69
	UAS CncC$^{\Delta1-87}$/Gal4^{Tub} RU486 10 μM	21.739	0.997	22	2.326	53	39	0.000	0.000	0.106		0.019	0.642	0.517	0.000	87/92
	UAS CncC$^{\Delta1-87}$/Gal4^{Tub} RU486 30 μM	18.375	1.043	16	1.058	39	37	0.000	0.000	0.003	0.019		0.178	0.01	0.143	72/78
	UAS CncC$^{\Delta1-87}$/Gal4^{Tub} RU486 60 μM	20.224	1.152	19	1.225	46	45	0.000	0.000	0.064	0.642	0.178		0.542	0.029	73/77
	UAS CncC$^{\Delta1-87}$/Gal4^{Tub} RU486 120 μM	21.878	1.199	20	2.360	48	39	0.000	0.000	0.194	0.517	0.01	0.542		0.000	75/77
	UAS CncC$^{\Delta1-87}$/Gal4^{Tub} RU486 320 μM	16.856	0.980	17	1.717	48	32	0.000	0.000	0.000	0.000	0.143	0.029	0.000		75/81

Figure	Sample	Mean LF	s.e.m.	Median LF	s.e.m.	% Median LF vs. control	Max (Days)	UAS CncC/Gal4^{Tub} RU486(-) - *Starvation*	UAS CncC/Gal4^{Tub} RU486(+) - *Starvation*	*Total Animals Died/Total*
Fig 3C	UAS CncC/Gal4^{Tub} RU486(-) - *Starvation*	8.733	0.167	9	0.186	100	12		0.000	90/90
	UAS CncC/Gal4^{Tub} RU486(+) - *Starvation*	4.422	0.147	4	0.12	44	8	0.000		90/90

Figure	Sample	Mean LF	s.e.m.	Median LF	s.e.m.	% Median LF vs. control	Max (Days)	UAS CncC/Gal4^{Tub} RU486(-) - *Increased flight periods*	UAS CncC/Gal4^{Tub} RU486(+) - *Increased flight periods*	*Total Animals Died/Total*
Fig. 3E$_2$	UAS CncC/Gal4^{Tub} RU486(-) - *Increased flight periods*	63.058	1.335	63	0.917	100	79		0.000	102/102
	UAS CncC/Gal4^{Tub} RU486(+) - *Increased flight periods*	16.00	0.569	18	1.194	28	28	0.000		146/146

Figure	Sample	Mean LF	s.e.m.	Median LF	s.e.m.	% Median LF vs. control	Max (Days)	UAS CncC/Gal4^{Tub} RU486(+)	UAS CncC, UAS In^{RNAi}/Gal4^{Tub} RU486(+)	UAS CncC, Pdpk1^{RNAi}/Gal4^{Tub} RU486(+)	*Total Animals Died/Total*
Fig. 5C	UAS CncC/Gal4^{Tub} RU486(+)	21.445	0.843	20	1.287	100	37		0.000	0.000	69/70
	UAS CncC, UAS In^{RNAi}/Gal4^{Tub} RU486(+)	26.60	0.997	26	2.047	130	50	0.000		0.202	105/105
	UAS CncC, UAS Pdpk1^{RNAi}/Gal4^{Tub} RU486(+)	26.274	0.772	26	0.555	130	42	0.000	0.202		124/124

Figure	Sample	Mean LF	s.e.m.	Median LF	s.e.m.	% Median LF vs. control	Max (Days)	UAS CncC/Gal4^{Tub} RU486(+)	UAS CncC/Gal4^{Tub} RU486(+) - CR	*Total Animals Died/Total*
Fig. 5D	UAS CncC/Gal4^{Tub} RU486(+)	22.95	0.781	23	1.112	100	37		0.000	80/80
	UAS CncC/Gal4^{Tub} RU486(+) - CR	34.319	1.186	35	2.200	152	48	0.000		76/80

Figure	Sample	Mean LF	s.e.m.	Median LF	s.e.m.	% Median LF vs. control	Max (Days)	UAS CncC/Gal4^{Tub} RU486(+)	UAS CncC, UAS Atg8α/Gal4^{Tub} RU486(+)	*Total Animals Died/Total*
Fig. S14B	UAS CncC/Gal4^{Tub} RU486(+)#	21.445	0.843	20	1.287	100	37		0.000	69/70
	UAS CncC, UAS Atg8α/Gal4^{Tub} RU486(+)	26.353	1.238	24	1.267	120	45	0.000		60/62

Figure	Sample	Mean LF	s.e.m.	Median LF	s.e.m.	% Median LF vs. control	Max (Days)	UAS CncC/Gal4^{Tub} RU486(+) - *Increased flight periods*	UAS CncC, UAS Gys^{RNAi}/Gal4^{Tub} RU486(+) - *Increased flight periods*	*Total Animals Died/Total*
Fig. S14F$_1$	UAS CncC/Gal4^{Tub} RU486(+) - *Increased flight periods**	16.00	0.569	18	1.194	100	28		0.000	146/146
	UAS CncC, UAS Gys^{RNAi}/Gal4^{Tub} RU486(+) - *Increased flight periods*	26.461	0.857	22	1.819	122	42	0.000		104/106

Figure	Sample	Mean LF	s.e.m.	Median LF	s.e.m.	% Median LF vs. control	Max (Days)	UAS CncC/Gal4^{Tub} RU486(+)	UAS CncC, UAS Gys^{RNAi}/Gal4^{Tub} RU486(+)	*Total Animals Died/Total*
Fig. S14F$_2$	UAS CncC/Gal4^{Tub} RU486(+)	23.173	0.636	23	0.748	100	37		0.000	110/110
	UAS CncC, UAS Gys^{RNAi}/Gal4^{Tub} RU486(+)	36.905	1.084	41	1.051	164	53	0.000		111/115

Figure	Sample	Mean LF	s.e.m.	Median LF	s.e.m.	% Median LF vs. control	Max (Days)	UAS CncC/Gal4^{Tub} RU486(+) - H_2O_2	UAS CncC, UAS dIlp2^{RNAi}/Gal4^{Tub} RU486(+) - H_2O_2 *Line 1*	UAS CncC, UAS dIlp2^{RNAi}/Gal4^{Tub} RU486(+) - H_2O_2 *Line 2*	*Total Animals Died/Total*
Fig. S17D	UAS CncC/Gal4^{Tub} RU486(+) - H_2O_2	7.250	0.732	6	0.545	100	17		0.68	0.92	20/20
	UAS CncC, UAS dIlp2^{RNAi}/Gal4^{Tub} RU486(+) - H_2O_2 *Line 1*	8.111	0.563	7	0.742	117	14	0.68		0.27	20/20
	UAS CncC, UAS dIlp2^{RNAi}/Gal4^{Tub} RU486(+) - H_2O_2 *Line 2*	7.370	0.463	6	0.519	100	11	0.92	0.27		20/20

Figure	Sample	Mean LF	s.e.m.	Median LF	s.e.m.	% Median LF vs. control	Max (Days)	UAS CncC/Gal4^{Tub} RU486(+)	UAS CncC, UAS dIlp2^{RNAi}/Gal4^{Tub} RU486(+) - *Line 1*	UAS CncC, UAS dIlp2^{RNAi}/Gal4^{Tub} RU486(+) - *Line 2*	*Total Animals Died/Total*
Fig. S17E	UAS CncC/Gal4^{Tub} RU486(+)	22.001	2.132	25	2.683	100	35		0.14	0.02	20/20
	UAS CncC, UAS dIlp2^{RNAi}/Gal4^{Tub} RU486(+) *Line 1*	28.005	2.340	30	2.225	120	40	0.14		0.65	20/20
	UAS CncC, UAS dIlp2^{RNAi}/Gal4^{Tub} RU486(+) *Line 2*	30.021	1.305	35	1.589	140	41	0.02	0.65		20/20

# same to Fig. 5C
* same to Fig. 3E$_2$

Supporting Table S2. Proteins found to be differentially expressed [*vs.* control RU486(-) flies] in flies' somatic tissues after inducible (for 7 days) ubiquitous CncC/Nrf2 overexpression (nano-LC-ESI-MS/MS proteomics analysis).

Uniprot_Acc	Fly GeneID	FlyBaseID	Fly Symbol	UAS cncC RU486(+) *vs.* UAS cncC RU486(-) L2R	UAS cncC RU486(+) *vs.* UAS cncC RU486(-) L2R_p.values	GO- Biological Process (UniProt; top 2 terms)	GO- Biological Process (UniProt; top 2 terms)
Q8IR80	32195	FBgn0030391	Rab40	0.840030174	1.58038E-09	Rab protein signal transductior	regulation of cell shape
P02517	39075	FBgn0001225	Hsp26	0.795289382	1.10052E-08	chaperone-mediated protein folding	cold acclimation
Q9VKC9	47253	FBgn0010497	dmGlut	0.762191572	4.33475E-08	glutamine metabolic process	L-glutamate import
Q9VRV8	38721	FBgn0024921	Trn	0.744550467	8.80111E-08	larval lymph gland hemopoiesis	neurogenesis
Q9VA15	43676	FBgn0039816	CG11317	0.704043193	4.21853E-07	neurogenesis	
Q6NL34	33865	FBgn0031782	WDR79	0.696204324	5.65924E-07	Cajal body organization	
P14199	35246	FBgn0003231	ref(2)P	0.66594156	1.70947E-06	mitochondrion organization	positive regulation of macroautophagy
Q9XZ61	39102	FBgn0011327	Uch-L5	0.661554497	1.99901E-06	neurogenesis	proteasome-mediated ubiquitin-dependent protein catabolic proces
O18413	33105	FBgn0020369	Rpt6	0.657154053	2.33645E-06	proteasome subunit	
Q9VNI4	40719	FBgn0037378	CG2046	0.634544619	5.12882E-06	proteasome core complex assembly	
P40301	41531	FBgn0086134	Prosalpha2	0.634097745	5.20781E-06	proteasome subunit	
Q9VDJ8	42450	FBgn0038819	Cpr92F	0.626809103	6.67284E-06	chitin-based cuticle developmen	
Q9VEC2	42174	FBgn0038577	CG12321	0.607876281	1.25489E-05	proteasome assembly	
Q9V3H2	33738	FBgn0028694	Rpn11	0.587888188	2.39787E-05	proteasome subunit	
Q9VLI0	34205	FBgn0032068	CG9466 (LManV)	0.57889639	3.18813E-05	mannose metabolic process	protein deglycosylation
O16043	35418	FBgn0022893	Df31	0.576131143	3.47722E-05	chromatin organization	nucleosome assembly
A1Z9C2	*Obsolete Entry*			0.566973535	4.62276E-05		
P23128	34364	FBgn0004419	me31B	0.561987655	5.38862E-05	cytoplasmic mRNA processing body assembly	gene silencing by miRNA
Q99323	38001	FBgn0265434	zip	0.557387215	6.20063E-05	anterior midgut development	border follicle cell migration
M9PG29	35015	FBgn0032609	CG13280	0.543540953	9.40106E-05		
Q7KMP8	42802	FBgn0028691	Rpn9	0.530473975	0.000138036	proteasome subunit	
Q9VED0	42165	FBgn0038569	CG7218	0.529564059	0.000141734		
A1Z7E8	35827	FBgn0033294	Mal-A4	0.527205138	0.000151759		
Q9VTF9	39254	FBgn0036136	Ufd1-like	0.526338004	0.00015561	cellular response to virus	positive regulation of proteasomal ubiquitin-dependent protein catabolic proces
Q9VLH9	34206	FBgn0032069	CG9468 (LManVI)	0.518378845	0.000195507	mannose metabolic process	protein deglycosylation
Q9U9Q1	34550	FBgn0028700	RfC38	0.499140433	0.000335124	DNA-dependent DNA replication	neurogenesis
Q9VW54	40174	FBgn0028695	Rpn1	0.492195172	0.000405294	proteasome subunit	
Q8T390	42265	FBgn0038659	EndoA	0.476779585	0.000612889	membrane tubulation	neurotransmitter secretion
Q9VSR7	39023	FBgn0035942	CG5660 (ValRS-m)	0.458648205	0.000982241	valyl-tRNA aminoacylation	
P23380	44307	FBgn0262736	Vha16-1	0.457324208	0.001016025	ATP hydrolysis coupled proton transpor	dsRNA transport
P54351	41694	FBgn0266464	Nsf2	0.449078463	0.001251819	ER to Golgi vesicle-mediated transpor	Golgi to plasma membrane protein transport
Q7K0W1	36584	FBgn0033918	CG8531	0.426052508	0.002203399	protein import into mitochondrial matrix	
Q9VC92	42901	FBgn0039184	CG6432	0.421803909	0.002438872	metabolic process	
Q8SWX4	326133	FBgn0051343	CG31343	0.417139253	0.002723764	peptide catabolic process	proteolysis
Q9VEZ2	41953	FBgn0038397	CG10185	0.416558245	0.002761303		
Q9V3V6	42805	FBgn0028684	Rpt5	0.416251388	0.002781318	proteasome subunit	
Q9VUJ1	39628	FBgn0023174	Prosbeta2	0.415449455	0.002834254	proteasome subunit	
Q9V405	32047	FBgn0028686	Rpt3	0.411167694	0.003132788	proteasome subunit	
Q9V3Z4	40717	FBgn0028690	Rpn5	0.410766783	0.00316216	proteasome subunit	
Q9V3G7	42641	FBgn0028688	Rpn7	0.406469773	0.003493105	proteasome subunit	
Q7K4H4	31374	FBgn0266570	CG2982	0.403572341	0.003733731	chromatin remodeling	histone H3-K36 demethylation
Q9VEK7	42092	FBgn0025456	CREG	0.402157526	0.00385662	negative regulation of transcription from RNA polymerase II promote	
Q7JW48	37058	FBgn0034295	CG10911	0.399130709	0.004131951		
P48601	42828	FBgn0015282	Rpt2	0.391380471	0.004920084	proteasome subunit	
P34082	31364	FBgn0000635	Fas2	0.388048792	0.005298863	axonal fasciculation	behavioral response to ethanol
Q86PD3	36233	FBgn0050022	CG30022	0.387523598	0.005360919	glutathione metabolic process	
Q9VDW6	42327	FBgn0260003	Dys	0.382747921	0.005956039	establishment of cell polarity	imaginal disc-derived wing vein morphogenesi
Q9XYN7	41079	FBgn0026380	Prosbeta3	0.380684029	0.006231172	proteasome subunit	
Q9XZJ4	45780	FBgn0263121	Prosalpha1	0.379510573	0.006392656	proteasome subunit	
Q9W2U8	50417	FBgn0083167	Neb-cGP	0.379119633	0.006447286	regulation of growth	
Q32KD4	36544	FBgn0262739	AGO1	0.376423611	0.006835598	chromatin silencing	female germ-line stem cell population maintenance
A0AQH0	46058	FBgn0010590	Prosbeta1	0.374827901	0.007075211	proteasome subunit	
Q7JRC3	251984	FBgn0010053	Jheh1	0.374827901	0.007075211	juvenile hormone catabolic process	
Q7KN90	36784	FBgn0027091	Aats-cys	0.37354566	0.007273188	cysteinyl-tRNA aminoacylation	
M9NEX3	31562	FBgn0029854	CG3566	0.37012998	0.007825046		
Q9VKZ8	34387	FBgn0032216	CG5384 (Usp14)	0.359396972	0.009811403	protein deubiquitination	protein ubiquitination
Q9VVM2	39992	FBgn0036766	CG5506	0.355943726	0.010539929		
B7Z0D3	38844	FBgn0035793	CG7546	0.35510361	0.010724287	negative regulation of apoptotic process	
Q9VHY6	40933	FBgn0037530	CG2943 (EMC1)	0.354649738	0.010825074	protein folding in endoplasmic reticulum	
P35122	33397	FBgn0010288	Uch	0.354649738	0.010825074	protein deubiquitination	ubiquitin-dependent protein catabolic process
Q9VSL2	38972	FBgn0035904	GstO3	0.34510815	0.013147988	glutathione metabolic process	
Q7KMQ0	35701	FBgn0028687	Rpt1	0.338999566	0.014857134	proteasome subunit	
O18680	37815	FBgn0020764	Alas	0.338236963	0.015083693	chitin-based cuticle developmen	heme biosynthetic process
Q9VSP9	39005	FBgn0035926	CG5804	0.33798254	0.015159953		
P51592	41181	FBgn0002431	hyd	0.336418144	0.015636364	compound eye developmen	eye-antennal disc morphogenesis
Q95083	36951	FBgn0016697	Prosalpha5	0.334301645	0.01630185	proteasome subunit	
A8JV19	32672	FBgn0030796	CG4829	0.331594565	0.017189221	glutathione metabolic process	neuron projection morphogenesis
M9PEJ3	38971	FBgn0035903	CG6765	0.323578225	0.020070108		
Q9VXE0	32636	FBgn0261791	SmG	0.319212344	0.021809801	mitotic spindle organization	mRNA splicing, via spliceosome
Q9VNA5	40639	FBgn0250746	Prosbeta7	0.319212344	0.021809801	proteasome subunit	
Q9VD01	42644	FBgn0038973	Pebp1	0.317271944	0.022624207	defense response to Gram-negative bacterium	defense response to Gram-positive bacterium
P55035	40388	FBgn0015283	Rpn10	0.314449438	0.02385598	proteasome subunit	
E2QCS8	326153	FBgn0031390	tho2	0.314449438	0.02385598	mRNA export from nucleus	mRNA export from nucleus in response to heat stres
Q7KN62	36040	FBgn0261014	TER94	0.314449438	0.02385598	cellular response to virus	dendrite morphogenesis
Q7K3Z3	35674	FBgn0033179	p47	0.314449438	0.02385598	Golgi organization	membrane fusion
Q7K485	45268	FBgn0029093	cathD	0.314449438	0.02385598	apoptotic process	autophagic cell death
Q9VZ35	32040	FBgn0030262	Vago	0.312780232	0.024611481	defense response to virus	

Q9VFZ4	41610	FBgn0038115	CG7966	0.309670755	0.026074251		
Q95U30	40905	FBgn0261393	alpha-Est5	0.305257645	0.028278991	modulation of synaptic transmissior	neuron cell-cell adhesior
M9PBS9	38750	FBgn0035715	CG10103	0.304345005	0.028754461	endosome transport via multivesicular body sorting pathwa	protein transport
P07909	43385	FBgn0001215	Hrb98DE	0.301223599	0.030433049	compound eye morphogenesis	female germ-line stem cell population maintenanc
Q9VMF0	33885	FBgn0031801	CG9498	0.300447094	0.030863473		
P11997	38015	FBgn0002564	Lsp1gamma	0.299684289	0.03129138		
Q9Y166	41640	FBgn0027610	Dic1	0.295238996	0.033887623	malate transmembrane transport	mitochondrial transport
P22769	32584	FBgn0004066	Prosalpha4	0.294320238	0.034446597	proteasome subunit	
Q8IMH4	43662	FBgn0000416	Sap-r	0.294320238	0.034446597	dsRNA transport	sphingolipid metabolic process
P12881	34359	FBgn0250843	Prosalpha6	0.294320238	0.034446597	proteasome subunit	
A8JNT7	5740624	FBgn0263606	Hsc20	0.2894743	0.037526931	iron-sulfur cluster assembly	protein folding
P25161	35176	FBgn0261396	Rpn3	0.284612031	0.040850178	proteasome subunit	
Q9VH72	41197	FBgn0037749	CG9471	0.284252262	0.041105686	multicellular organism reproductio	
Q8IRD3	38413	FBgn0035438	PHGPx	0.284230679	0.041121057	response to lipid hydroperoxide	response to oxidative stress
Q7K148	45269	FBgn0029134	Prosbeta5	0.279861675	0.044334506	proteasome subunit	
Q7KSM5	41532	FBgn0029512	Aos1	0.274710674	0.048392842	neurogenesis	positive regulation of MyD88-dependent toll-like receptor signaling pathwa
Q7K3B7	37285	FBgn0034488	CG11208	0.274173382	0.048833586	fatty acid alpha-oxidation	
Q7KSK2	41717	FBgn0038205	Kif19A	-0.272925348	0.049870375	microtubule-based movement	
A1Z9F9	36511	FBgn0261989	CG42807	-0.272925348	0.049870375		
M9PCP3	33825	FBgn0031752	CG9044	-0.273231983	0.049613946	protein localization	
Q8IN44	44121	FBgn0028396	TotA	-0.273845058	0.049104568	cellular response to hea	cellular response to mechanical stimulus
Q86BR8	42433	FBgn0038805	TFAM	-0.277753573	0.045959312	mitochondrial DNA replicatior	positive regulation of transcription, DNA-templated
Q24524	31717	FBgn0003447	sn	-0.278700459	0.045223404	actin cytoskeleton organizatior	actin filament bundle assembl
Q9VSL3	38973	FBgn0086348	se	-0.279007094	0.044987231	eye pigment biosynthetic process	glutathione metabolic process
Q7KV99	38286	FBgn0035321	CG1275	-0.279313665	0.044752148	oxidation-reduction process	
Q9VVL5	39986	FBgn0036760	CG5567	-0.283913619	0.041347426	dephosphorylatior	protein dephosphorylation
A1Z8F4	36171	FBgn0003396	shn	-0.284514967	0.04091898	cell proliferation	compound eye morphogenesis
Q9VJY6	34754	FBgn0032518	RpL24	-0.288946793	0.037875973	assembly of large subunit precursor of preribosome	centrosome duplication
Q9VH39	41227	FBgn0037777	CG11722	-0.289920905	0.037233563	mitochondrial respiratory chain complex I assembl	
Q9VGT0	41333	FBgn0026314	Ugt35b	-0.289920905	0.037233563	flavonoid biosynthetic process	flavonoid glucuronidatior
Q9VP61	40348	FBgn0012034	AcCoAS	-0.289920905	0.037233563	acetyl-CoA biosynthetic process from acetate	acetyl-CoA metabolic process
Q967S0	38753	FBgn0041194	Prat2	-0.295349442	0.033820953	'de novo' IMP biosynthetic process	nucleoside metabolic process
P54192	33040	FBgn0011280	Obp19d	-0.295650148	0.033640001	sensory perception of chemical stimulus	
Q9VA83	44965	FBgn0015221	Fer2LCH	-0.29595079	0.033459916	cellular iron ion homeostasis	chaeta development
Q9VCI3	42810	FBgn0039114	Lsd-1	-0.29595079	0.033459916	lipid particle organization	lipid storage
Q7KUQ6	39889	FBgn0261565	Lmpt	-0.296972239	0.032854214	defense response to fungus	
O97479	40836	FBgn0024289	Sodh-1	-0.30080076	0.030666789	sensory perception of pain	sleep
P08171	39392	FBgn0000592	Est-6	-0.30080076	0.030666789	courtship behavior	mating
Q9VQL7	33524	FBgn0027571	CG3523 (FASN1)	-0.30080076	0.030666789	cellular response to sucrose stimulus	glycogen metabolic process
P35500	32619	FBgn0264255	para	-0.301101528	0.030500371	male courtship behavior, veined wing generated song productic	mechanosensory behavior
Q04691	39566	FBgn0000639	Fbp1	-0.301402233	0.030334764	storage protein import into fat body	
Q9XYZ9	37960	FBgn0027590	GstE12	-0.301402233	0.030334764	glutathione metabolic process	
Q9VA31	43658	FBgn0039806	CG15545	-0.301702876	0.030169964		
Q9V3S0	30986	FBgn0010019	Cyp4g1	-0.306575871	0.027604184	lipid metabolic process	
Q9W5B4	31055	FBgn0040398	CG14629	-0.306876639	0.027452179		
Q9VXG9	32606	FBgn0030745	CG4239	-0.317256617	0.022630744	monovalent inorganic cation transport	
P54353	33111	FBgn0015379	dod	-0.322690799	0.02041358	epidermal growth factor receptor signaling pathwa	
Q9VLS9	34106	FBgn0031987	CG12375	-0.325154352	0.019472501		
Q9VF15	41930	FBgn0027657	glob1	-0.325154352	0.019472501	oxygen transport	sleep
Q7KRU8	46415	FBgn0015222	Fer1HCH	-0.326056468	0.019137497	cell proliferation	cellular iron ion homeostasis
Q9W306	31923	FBgn0030160	CG9691	-0.329032946	0.018067644		
Q9VRJ1	38595	FBgn0028962	Aats-ala-m	-0.329622766	0.017861974	alanyl-tRNA aminoacylation	sensory perception of pain
P62152	36329	FBgn0000253	Cam	-0.331629452	0.017177521	actin filament-based movement	adaptation of rhodopsin mediated signaling
P02843	31939	FBgn0004045	Yp1	-0.335317727	0.015979321	sex differentiation	vitellogenesis
P06607	32339	FBgn0004047	Yp3	-0.335317727	0.015979321	embryo development	neurogenesis
Q6NR72	37739	FBgn0034885	CG4019	-0.335907789	0.015794608	renal system process	transmembrane transport
Q9I7Q5	59158	FBgn0042119	Cpr65Au	-0.342005481	0.013993056	chitin-based cuticle development	
Q9W380	50405	FBgn0040931	CG9034	-0.342300361	0.013910728		
Q95U34	39031	FBgn0263199	Galk	-0.346946666	0.012668814	galactose metabolic process	
P07668	42249	FBgn0000303	Cha	-0.353091537	0.011177528	acetylcholine biosynthetic process	nervous system developmen
C9QPE7	34016	FBgn0031907	CG5171 (Tpp)	-0.353681478	0.011042906	trehalose biosynthetic process	
Q8INW5	35222	FBgn0032783	CG10237	-0.370784647	0.007716455		
Q7JYW9	44008	FBgn0001187	Hex-C	-0.377863302	0.006625698	cellular glucose homeostasis	glycolytic process
Q9Y143	34728	FBgn0027586	CG5867	-0.378158303	0.006583411	circadian rhythm	multicellular organism reproductio
A1Z7Z4	36009	FBgn0033446	CG1648	-0.378453243	0.006541374	protein transport	
O76454	43499	FBgn0024841	Pcd	-0.392233996	0.004827072	tetrahydrobiopterin biosynthetic process	
Q9VP02	40406	FBgn0043783	CG32444	-0.403821636	0.00371245	hexose metabolic process	
B7Z061	39812	FBgn0011693	Pdh	-0.405269757	0.003590998	phagocytosis	retinal metabolic process
Q29R16	44297	FBgn0259175	ome	-0.412383315	0.003045244		
Q9VY98	32330	FBgn0030514	CG9941	-0.433014401	0.001862177	protein ubiquitinatior	
Q2MGJ8	3771756	FBgn0038033	CG10097	-0.43671947	0.001701059		
Q9W1C9	49821	FBgn0011695	PebIII	-0.442372733	0.001479784	metamorphosis	post-mating behavior
Q9VIQ6	35281	FBgn0032835	CG16772	-0.443535793	0.001437686		
Q9Y119	33642	FBgn0027560	Tps1	-0.46288254	0.000881051	photoreceptor cell maintenance	trehalose biosynthetic process
Q7K483	43574	FBgn0039741	CG7943	-0.494861701	0.00037687	mitochondrial transpor	translation
Q8IQQ0	39899	FBgn0010352	Nc73EF	-0.521275285	0.000179989	tricarboxylic acid cycle	
M9NEN9	33361	FBgn0051665	wry	-0.531835513	0.000132673	heart process	Notch signaling pathway
Q9W3W8	31613	FBgn0029897	RpL17	-0.577971625	3.28217E-05	centrosome duplication	mitotic spindle elongation
P22817	40248	FBgn0001247	Ide	-0.609360225	1.19505E-05	defense response to Gram-negative bacteriun	determination of adult lifespan S
Q9W5E4	31010	FBgn0001341	l(1)1Bi	-0.63046666	5.89427E-06		
A1Z8H0	36186	FBgn0085256	CG34227	-0.742151865	9.67908E-08		
O97066	40815	FBgn0262801	twr	-1.035514095	1.00252E-13	signal peptide processing	
Q9V449	44482	FBgn0028416	Met75Ca	-1.316378123	3.11838E-21	multicellular organism reproductior	

Supporting Table S3. Human orthologs of fly proteins identified to be differentially expressed after inducible CncC/Nrf2 overexpression (nano-LC-ESI-MS/MS proteomics analysis).

Uniprot_Acc_Fly Search Term	Fly GeneID	FlyBaseID	Fly Symbol	Human GeneID	HGNCID	Human Symbol	DIOPT Score	Weighted Score	Rank	Best Score	Best Score Reverse	Prediction Derived From
Q8IR80	32195	FBgn0030391	Rab40	57799	18285	RAB40C	10	9.669	high	Yes	Yes	Compara, Homologene, Inparanoid, Isobase, OMA, OrthoDB, orthoMCL, Phylome, RoundUp, TreeFar
Q8IR80	32195	FBgn0030391	Rab40	10966	18284	RAB40B	7	6.819	moderate	No	Yes	Compara, Inparanoid, OMA, OrthoDB, Phylome, RoundUp, TreeFan
Q8IR80	32195	FBgn0030391	Rab40	142684	18283	RAB40A	5	4.806	moderate	No	Yes	Compara, Inparanoid, OrthoDB, Phylome, TreeFam
Q8IR80	32195	FBgn0030391	Rab40	282808	25410	RAB40AL	5	4.806	moderate	No	Yes	Compara, Inparanoid, OrthoDB, Phylome, TreeFam
P02517	39075	FBgn0001225	Hsp26	3315	5246	HSPB1	3	2.934	moderate	Yes	No	Compara, OrthoDB, RoundUp
P02517	39075	FBgn0001225	Hsp26	1410	2389	CRYAB	3	2.833	moderate	Yes	No	Compara, orthoMCL, RoundUp
P02517	39075	FBgn0001225	Hsp26	102724652	-	LOC102724652	2	1.933	low	No	No	Compara, RoundUp
P02517	39075	FBgn0001225	Hsp26	126393	26511	HSPB6	2	1.931	low	No	No	Compara, OrthoDB
P02517	39075	FBgn0001225	Hsp26	26353	30171	HSPB8	2	1.931	low	No	No	Compara, OrthoDB
P02517	39075	FBgn0001225	Hsp26	1409	2388	CRYAA	2	1.931	low	No	No	Compara, OrthoDB
P02517	39075	FBgn0001225	Hsp26	8988	5248	HSPB3	2	1.931	low	No	No	Compara, OrthoDB
P02517	39075	FBgn0001225	Hsp26	3316	5247	HSPB2	2	1.89	moderate	No	Yes	Compara, TreeFam
Q9VKC9	47253	FBgn0010497	dmGlut	246213	20151	SLC17A8	2	2.004	moderate	Yes	No	OrthoDB, RoundUp
Q9VKC9	47253	FBgn0010497	dmGlut	57084	16703	SLC17A6	2	2.004	moderate	Yes	No	OrthoDB, RoundUp
Q9VKC9	47253	FBgn0010497	dmGlut	57030	16704	SLC17A7	2	2.004	moderate	Yes	No	OrthoDB, RoundUp
Q9VKC9	47253	FBgn0010497	dmGlut	10786	10931	SLC17A3	2	1.951	high	Yes	Yes	Isobase, OrthoDB
Q9VKC9	47253	FBgn0010497	dmGlut	10050	10932	SLC17A4	1	1.001	low	No	No	OrthoDB
Q9VKC9	47253	FBgn0010497	dmGlut	26503	10933	SLC17A5	1	1.001	low	No	No	OrthoDB
Q9VKC9	47253	FBgn0010497	dmGlut	10246	10930	SLC17A2	1	1.001	low	No	No	OrthoDB
Q9VRV8	38721	FBgn0024921	Trn	3842	6401	TNPO1	9	8.719	high	Yes	Yes	Compara, Homologene, Inparanoid, OMA, OrthoDB, orthoMCL, Phylome, RoundUp, TreeFar
Q9VRV8	38721	FBgn0024921	Trn	30000	19998	TNPO2	7	6.709	moderate	No	Yes	Compara, Inparanoid, OrthoDB, orthoMCL, Phylome, RoundUp, TreeFan
Q9VA15	43676	FBgn0039816	CG11317				0	0	None			
Q6NL34	33865	FBgn0031782	WDR79	55135	25522	WRAP53	10	9.669	high	Yes	Yes	Compara, Homologene, Inparanoid, Isobase, OMA, OrthoDB, orthoMCL, Phylome, RoundUp, TreeFar
P14199	35246	FBgn0003231	ref(2)P	8878	11280	SQSTM1	4	3.864	high	Yes	Yes	Isobase, OrthoDB, Phylome, RoundUp
Q9XZ61	39102	FBgn0011327	Uch-L5	51377	19678	UCHL5	10	9.669	high	Yes	Yes	Compara, Homologene, Inparanoid, Isobase, OMA, OrthoDB, orthoMCL, Phylome, RoundUp, TreeFar
Q9XZ61	39102	FBgn0011327	Uch-L5	8314	950	BAP1	1	1.001	low	No	No	OrthoDB
O18413	33105	FBgn0020369	Rpt6	5705	9552	PSMC5	10	9.669	high	Yes	Yes	Compara, Homologene, Inparanoid, Isobase, OMA, OrthoDB, orthoMCL, Phylome, RoundUp, TreeFar
O18413	33105	FBgn0020369	Rpt6	5706	9553	PSMC6	1	1.001	low	No	No	OrthoDB
O18413	33105	FBgn0020369	Rpt6	5701	9548	PSMC2	1	1.001	low	No	No	OrthoDB
O18413	33105	FBgn0020369	Rpt6	55759	14098	WDR12	1	1.001	low	No	No	OrthoDB
O18413	33105	FBgn0020369	Rpt6	79170	28149	PRR15L	1	0.91	low	No	Yes	Phylome
Q9VNI4	40719	FBgn0037378	CG2046				0	0	None			
P40301	41531	FBgn0086134	Prosalpha2	5683	9531	PSMA2	9	8.668	high	Yes	Yes	Compara, Homologene, Inparanoid, Isobase, OMA, orthoMCL, Phylome, RoundUp, TreeFar
Q9VDJ8	42450	FBgn0038819	Cpr92F				0	0	None			
Q9VEC2	42174	FBgn0038577	CG12321	56984	24929	PSMG2	9	8.719	high	Yes	Yes	Compara, Homologene, Inparanoid, OMA, OrthoDB, orthoMCL, Phylome, RoundUp, TreeFar
Q9V3H2	33738	FBgn0028694	Rpn11	10213	16889	PSMD14	9	8.659	high	Yes	Yes	Compara, Homologene, Inparanoid, Isobase, OrthoDB, orthoMCL, Phylome, RoundUp, TreeFar
Q9VLI0	34205	FBgn0032068	CG9466	4125	6826	MAN2B1	7	6.656	moderate	Yes	No	Compara, Inparanoid, Isobase, OrthoDB, orthoMCL, Phylome, TreeFam
O16043	35418	FBgn0022893	Df31				0	0	None			
A1Z9C2												
P23128	34364	FBgn0004419	me31B	1656	2747	DDX6	10	9.669	high	Yes	Yes	Compara, Homologene, Inparanoid, Isobase, OMA, OrthoDB, orthoMCL, Phylome, RoundUp, TreeFar
Q99323	38001	FBgn0265434	zip	4628	7568	MYH10	9	8.659	high	Yes	Yes	Compara, Homologene, Inparanoid, Isobase, OrthoDB, orthoMCL, Phylome, RoundUp, TreeFar
Q99323	38001	FBgn0265434	zip	4627	7579	MYH9	7	6.709	moderate	No	Yes	Compara, Inparanoid, OrthoDB, orthoMCL, Phylome, RoundUp, TreeFan
Q99323	38001	FBgn0265434	zip	4629	7569	MYH11	6	5.706	moderate	No	Yes	Compara, Inparanoid, OrthoDB, orthoMCL, Phylome, TreeFam
Q99323	38001	FBgn0265434	zip	79784	23212	MYH14	6	5.706	moderate	No	Yes	Compara, Inparanoid, OrthoDB, orthoMCL, Phylome, TreeFam
Q99323	38001	FBgn0265434	zip	4645	7603	MYO5B	1	0.9	low	No	No	orthoMCL
Q99323	38001	FBgn0265434	zip	4622	7574	MYH4	1	0.9	low	No	No	orthoMCL
Q99323	38001	FBgn0265434	zip	4625	7577	MYH7	1	0.9	low	No	No	orthoMCL
Q99323	38001	FBgn0265434	zip	8735	7571	MYH13	1	0.9	low	No	No	orthoMCL
Q99323	38001	FBgn0265434	zip	57644	15906	MYH7B	1	0.9	low	No	No	orthoMCL
Q99323	38001	FBgn0265434	zip	4619	7567	MYH1	1	0.9	low	No	No	orthoMCL
Q99323	38001	FBgn0265434	zip	55930	7604	MYO5C	1	0.9	low	No	No	orthoMCL
Q99323	38001	FBgn0265434	zip	4621	7573	MYH3	1	0.9	low	No	No	orthoMCL
Q99323	38001	FBgn0265434	zip	4626	7578	MYH8	1	0.9	low	No	No	orthoMCL
Q99323	38001	FBgn0265434	zip	22989	31073	MYH15	1	0.9	low	No	No	orthoMCL
Q99323	38001	FBgn0265434	zip	4624	7576	MYH6	1	0.9	low	No	No	orthoMCL
Q99323	38001	FBgn0265434	zip	4620	7572	MYH2	1	0.9	low	No	No	orthoMCL
Q99323	38001	FBgn0265434	zip	4644	7602	MYO5A	1	0.9	low	No	No	orthoMCL
M9PG29	35015	FBgn0032609	CG13280	27294	17887	DHDH	5	4.751	moderate	Yes	No	Compara, Isobase, OrthoDB, Phylome, TreeFam
Q7KMP8	42802	FBgn0028691	Rpn9	5719	9558	PSMD13	10	9.669	high	Yes	Yes	Compara, Homologene, Inparanoid, Isobase, OMA, OrthoDB, orthoMCL, Phylome, RoundUp, TreeFar
Q9VED0	42165	FBgn0038569	CG7218	202018	26887	TAPT1	8	7.656	high	Yes	Yes	Compara, Homologene, Inparanoid, Isobase, OrthoDB, orthoMCL, Phylome, TreeFan
A1Z7E8	35827	FBgn0033294	Mal-A4	6519	11025	SLC3A1	7	6.699	moderate	Yes	No	Compara, Inparanoid, Isobase, OrthoDB, orthoMCL, Phylome, RoundUp
A1Z7E8	35827	FBgn0033294	Mal-A4	6520	11026	SLC3A2	1	1.003	low	No	No	RoundUp
Q9VTF9	39254	FBgn0036136	Ufd1-like	7353	12520	UFD1L	10	9.669	high	Yes	Yes	Compara, Homologene, Inparanoid, Isobase, OMA, OrthoDB, orthoMCL, Phylome, RoundUp, TreeFar
Q9VLH9	34206	FBgn0032069	CG9468	4125	6826	MAN2B1	5	4.701	moderate	Yes	No	Compara, OrthoDB, orthoMCL, Phylome, TreeFam
Q9VLH9	34206	FBgn0032069	CG9468	23324	29623	MAN2B2	1	0.95	low	No	Yes	Isobase
Q9U9Q1	34550	FBgn0028700	RfC38	5983	9971	RFC3	9	8.719	high	Yes	Yes	Compara, Homologene, Inparanoid, OMA, OrthoDB, orthoMCL, Phylome, RoundUp, TreeFar
Q9VW54	40174	FBgn0028695	Rpn1	5708	9559	PSMD2	9	8.719	high	Yes	Yes	Compara, Homologene, Inparanoid, OMA, OrthoDB, orthoMCL, Phylome, RoundUp, TreeFar
Q8T390	42265	FBgn0038659	EndoA	6457	10832	SH3GL3	10	9.669	high	Yes	Yes	Compara, Homologene, Inparanoid, Isobase, OMA, OrthoDB, orthoMCL, Phylome, RoundUp, TreeFar
Q8T390	42265	FBgn0038659	EndoA	6456	10831	SH3GL2	8	7.719	moderate	No	Yes	Compara, Inparanoid, OMA, OrthoDB, orthoMCL, Phylome, RoundUp, TreeFan
Q8T390	42265	FBgn0038659	EndoA	6455	10830	SH3GL1	8	7.719	moderate	No	Yes	Compara, Inparanoid, OMA, OrthoDB, orthoMCL, Phylome, RoundUp, TreeFan
Q8T390	42265	FBgn0038659	EndoA	2885	4566	GRB2	1	1.003	low	No	No	RoundUp
Q8T390	42265	FBgn0038659	EndoA	10254	11358	STAM2	1	1.003	low	No	No	RoundUp
Q8T390	42265	FBgn0038659	EndoA	8027	11357	STAM	1	1.003	low	No	No	RoundUp
Q9VSR7	39023	FBgn0035942	CG5660	7407	12651	VARS	3	2.851	moderate	Yes	No	Isobase, OrthoDB, orthoMCL
Q9VSR7	39023	FBgn0035942	CG5660	57176	21642	VARS2	3	2.811	high	Yes	Yes	OrthoDB, orthoMCL, Phylome
P23380	44307	FBgn0262736	Vha16-1	527	855	ATP6V0C	8	7.716	moderate	Yes	No	Compara, Homologene, Inparanoid, OMA, OrthoDB, orthoMCL, Phylome, TreeFan
P54351	41694	FBgn0266464	Nsf2	4905	8016	NSF	9	8.666	high	Yes	Yes	Compara, Homologene, Inparanoid, Isobase, OMA, OrthoDB, orthoMCL, Phylome, TreeFam
Q7K0W1	36584	FBgn0033918	CG8531	55735	25570	DNAJC11	9	8.719	high	Yes	Yes	Compara, Homologene, Inparanoid, OMA, OrthoDB, orthoMCL, Phylome, RoundUp, TreeFar
Q9VC92	42901	FBgn0039184	CG6432	79611	24723	ACSS3	10	9.669	high	Yes	Yes	Compara, Homologene, Inparanoid, Isobase, OMA, OrthoDB, orthoMCL, Phylome, RoundUp, TreeFar
Q9VC92	42901	FBgn0039184	CG6432	84532	16091	ACSS1	1	0.9	low	No	No	orthoMCL
Q9VC92	42901	FBgn0039184	CG6432	55902	15814	ACSS2	1	0.9	low	No	No	orthoMCL
Q8SWX4	326133	FBgn0051343	CG31343	29953	30748	TRHDE	3	2.841	high	Yes	Yes	Compara, OrthoDB, Phylome
Q8SWX4	326133	FBgn0051343	CG31343	290	500	ANPEP	3	2.811	moderate	Yes	No	OrthoDB, orthoMCL, Phylome
Q8SWX4	326133	FBgn0051343	CG31343	9520	7900	NPEPPS	2	1.951	low	No	No	Isobase, OrthoDB
Q8SWX4	326133	FBgn0051343	CG31343	206338	26904	LVRN	2	1.911	low	No	No	OrthoDB, Phylome
Q8SWX4	326133	FBgn0051343	CG31343	2028	3355	ENPEP	2	1.901	low	No	No	OrthoDB, orthoMCL
Q8SWX4	326133	FBgn0051343	CG31343	51752	18173	ERAP1	1	1.001	low	No	No	OrthoDB

Q8SWX4	326133	FBgn0051343	CG31343	64167	29499	ERAP2	1	1.001	low	No	No	OrthoDB
Q8SWX4	326133	FBgn0051343	CG31343	4012	6656	LNPEP	1	1.001	low	No	No	OrthoDB
Q9VEZ2	41953	FBgn0038397	CG10185	57495	29229	NWD2	8	7.709	high	Yes	Yes	Compara, Homologene, Inparanoid, OrthoDB, orthoMCL, Phylome, RoundUp, TreeFar
Q9VEZ2	41953	FBgn0038397	CG10185	284434	27619	NWD1	3	2.833	moderate	No	Yes	Compara, orthoMCL, RoundUp
Q9V3V6	42805	FBgn0028684	Rpt5	5702	9549	PSMC3	9	8.666	high	Yes	Yes	Compara, Homologene, Inparanoid, Isobase, OMA, OrthoDB, orthoMCL, Phylome, TreeFan
Q9VUJ1	39628	FBgn0023174	Prosbeta2	5695	9544	PSMB7	10	9.669	high	Yes	Yes	Compara, Homologene, Inparanoid, Isobase, OMA, OrthoDB, orthoMCL, Phylome, RoundUp, TreeFar
Q9VUJ1	39628	FBgn0023174	Prosbeta2	5699	9538	PSMB10	4	3.801	moderate	No	Yes	Compara, OrthoDB, Phylome, TreeFam
Q9V405	32047	FBgn0028686	Rpt3	5704	9551	PSMC4	10	9.669	high	Yes	Yes	Compara, Homologene, Inparanoid, Isobase, OMA, OrthoDB, orthoMCL, Phylome, RoundUp, TreeFar
Q9V405	32047	FBgn0028686	Rpt3	5700	9547	PSMC1	1	1.001	low	No	No	OrthoDB
Q9V3Z4	40717	FBgn0028690	Rpn5	5718	9557	PSMD12	10	9.669	high	Yes	Yes	Compara, Homologene, Inparanoid, Isobase, OMA, OrthoDB, orthoMCL, Phylome, RoundUp, TreeFar
Q9V3G7	42641	FBgn0028688	Rpn7	9861	9564	PSMD6	9	8.659	high	Yes	Yes	Compara, Homologene, Inparanoid, Isobase, OrthoDB, orthoMCL, Phylome, RoundUp, TreeFar
Q7K4H4	31374	FBgn0266570	CG2982	79697	20968	C14orf169	4	3.938	high	Yes	Yes	Compara, Homologene, Inparanoid, RoundU
Q7K4H4	31374	FBgn0266570	CG2982	84864	19441	MINA	4	3.811	high	Yes	Yes	Isobase, OrthoDB, orthoMCL, TreeFam
Q9VEK7	42092	FBgn0025456	CREG	8804	2351	CREG1	9	8.719	high	Yes	Yes	Compara, Homologene, Inparanoid, OMA, OrthoDB, orthoMCL, Phylome, RoundUp, TreeFar
Q9VEK7	42092	FBgn0025456	CREG	200407	14272	CREG2	5	4.746	moderate	No	Yes	Compara, Inparanoid, OrthoDB, orthoMCL, Phylome
Q7JW48	37058	FBgn0034295	CG10911				0	0	None			
P48601	42828	FBgn0015282	Rpt2	5700	9547	PSMC1	9	8.719	high	Yes	Yes	Compara, Homologene, Inparanoid, OMA, OrthoDB, orthoMCL, Phylome, RoundUp, TreeFar
P48601	42828	FBgn0015282	Rpt2	5704	9551	PSMC4	1	1.001	low	No	No	OrthoDB
P34082	31364	FBgn0000635	Fas2	4685	7657	NCAM2	7	6.718	high	Yes	Yes	Compara, Inparanoid, OMA, orthoMCL, Phylome, RoundUp, TreeFan
P34082	31364	FBgn0000635	Fas2	4684	7656	NCAM1	7	6.715	high	Yes	Yes	Compara, Homologene, Inparanoid, OMA, orthoMCL, Phylome, TreeFan
P34082	31364	FBgn0000635	Fas2	81607	19688	PVRL4	1	0.91	low	No	Yes	Phylome
Q86PD3	36233	FBgn0050022	CG30022	23474	23287	ETHE1	9	8.719	high	Yes	Yes	Compara, Homologene, Inparanoid, OMA, OrthoDB, orthoMCL, Phylome, RoundUp, TreeFar
Q9VDW6	42327	FBgn0260003	Dys	1756	2928	DMD	9	8.719	high	Yes	Yes	Compara, Homologene, Inparanoid, OMA, OrthoDB, orthoMCL, Phylome, RoundUp, TreeFar
Q9VDW6	42327	FBgn0260003	Dys	7402	12635	UTRN	6	5.706	moderate	No	Yes	Compara, Inparanoid, OrthoDB, orthoMCL, Phylome, TreeFam
Q9VDW6	42327	FBgn0260003	Dys	1821	3032	DRP2	4	3.743	moderate	No	Yes	Compara, orthoMCL, Phylome, RoundUp
Q9XYN7	41079	FBgn0026380	Prosbeta3	5691	9540	PSMB3	10	9.669	high	Yes	Yes	Compara, Homologene, Inparanoid, Isobase, OMA, OrthoDB, orthoMCL, Phylome, RoundUp, TreeFar
Q9XZJ4	45780	FBgn0263121	Prosalpha1	5687	9535	PSMA6	8	7.718	high	Yes	Yes	Compara, Homologene, Inparanoid, OMA, orthoMCL, Phylome, RoundUp, TreeFar
Q9XZJ4	246582	FBgn0050382	CG30382	5687	9535	PSMA6	7	6.808	moderate	Yes	No	Compara, Homologene, Inparanoid, OMA, orthoMCL, RoundUp, TreeFan
Q9W2U8	50417	FBgn0083167	Neb-cGP	84833	30889	USMG5	3	2.811	moderate	Yes	No	OrthoDB, orthoMCL, Phylome
Q32KD4	36544	FBgn0262739	AGO1	26523	3262	AGO1	8	7.719	high	Yes	Yes	Compara, Inparanoid, OMA, OrthoDB, orthoMCL, Phylome, RoundUp, TreeFan
Q32KD4	36544	FBgn0262739	AGO1	27161	3263	AGO2	8	7.709	high	Yes	Yes	Compara, Homologene, Inparanoid, OrthoDB, orthoMCL, Phylome, RoundUp, TreeFar
Q32KD4	36544	FBgn0262739	AGO1	192670	18424	AGO4	7	6.716	moderate	No	Yes	Compara, Inparanoid, OMA, OrthoDB, orthoMCL, Phylome, TreeFam
Q32KD4	36544	FBgn0262739	AGO1	192669	18421	AGO3	7	6.709	moderate	No	Yes	Compara, Inparanoid, OrthoDB, orthoMCL, Phylome, RoundUp, TreeFan
A0AQH0	46058	FBgn0010590	Prosbeta1	5694	9543	PSMB6	9	8.669	high	Yes	Yes	Compara, Inparanoid, Isobase, OMA, OrthoDB, orthoMCL, Phylome, RoundUp, TreeFan
A0AQH0	46058	FBgn0010590	Prosbeta1	5698	9546	PSMB9	5	4.804	moderate	No	Yes	Compara, OrthoDB, Phylome, RoundUp, TreeFan
Q7JRC3	251984	FBgn0010053	Jheh1	2052	3401	EPHX1	9	8.669	high	Yes	Yes	Compara, Inparanoid, Isobase, OMA, OrthoDB, orthoMCL, Phylome, RoundUp, TreeFan
Q7JRC3	251984	FBgn0010053	Jheh1	2053	3402	EPHX2	1	1.003	low	No	Yes	RoundUp
Q7JRC3	251984	FBgn0010053	Jheh1	253152	23758	EPHX4	1	1.003	low	No	No	RoundUp
Q7JRC3	251984	FBgn0010053	Jheh1	79852	23760	EPHX3	1	1.003	low	No	Yes	RoundUp
Q7KN90	36784	FBgn0027091	Aats-cys	833	1493	CARS	10	9.669	high	Yes	Yes	Compara, Homologene, Inparanoid, Isobase, OMA, OrthoDB, orthoMCL, Phylome, RoundUp, TreeFar
Q7KN90	36784	FBgn0027091	Aats-cys	79587	25695	CARS2	1	0.9	low	No	No	orthoMCL
M9NEX3	31562	FBgn0029854	CG3566	80777	24374	CYB5B	5	4.794	moderate	Yes	No	Compara, OrthoDB, orthoMCL, RoundUp, TreeFam
M9NEX3	31562	FBgn0029854	CG3566	1528	2570	CYB5A	5	4.794	moderate	Yes	No	Compara, OrthoDB, orthoMCL, RoundUp, TreeFam
M9NEX3	31562	FBgn0029854	CG3566	79152	21197	FA2H	1	1.003	low	No	No	RoundUp
Q9VKZ8	34387	FBgn0032216	CG5384	9097	12612	USP14	9	8.719	high	Yes	Yes	Compara, Homologene, Inparanoid, OMA, OrthoDB, orthoMCL, Phylome, RoundUp, TreeFar
Q9VVM2	39992	FBgn0036766	CG5506				0	0	None			
B7Z0D3	38844	FBgn0035793	CG7546	7917	13919	BAG6	6	5.808	high	Yes	Yes	Compara, Inparanoid, OMA, orthoMCL, RoundUp, TreeFam
B7Z0D3	38844	FBgn0035793	CG7546	8266	12505	UBL4A	1	0.91	low	No	No	Phylome
B7Z0D3	38844	FBgn0035793	CG7546	164153	32309	UBL4B	1	0.91	low	No	No	Phylome
Q9VHY6	40933	FBgn0037530	CG2943	23065	28957	EMC1	8	7.709	high	Yes	Yes	Compara, Homologene, Inparanoid, OrthoDB, orthoMCL, Phylome, RoundUp, TreeFar
P35122	33397	FBgn0010288	Uch	7347	12515	UCHL3	8	7.759	high	Yes	Yes	Compara, Homologene, Inparanoid, Isobase, OrthoDB, Phylome, RoundUp, TreeFar
P35122	33397	FBgn0010288	Uch	7345	12513	UCHL1	7	6.709	moderate	No	Yes	Compara, Inparanoid, OrthoDB, orthoMCL, Phylome, RoundUp, TreeFan
Q9VSL2	38972	FBgn0035904	GstO3	9446	13312	GSTO1	10	9.669	high	Yes	Yes	Compara, Homologene, Inparanoid, Isobase, OMA, OrthoDB, orthoMCL, Phylome, RoundUp, TreeFar
Q9VSL2	38972	FBgn0035904	GstO3	119391	23064	GSTO2	7	6.819	moderate	No	No	Compara, Inparanoid, OMA, OrthoDB, Phylome, RoundUp, TreeFan
Q7KMQ0	35701	FBgn0028687	Rpt1	5701	9548	PSMC2	9	8.666	high	Yes	Yes	Compara, Homologene, Inparanoid, Isobase, OMA, OrthoDB, orthoMCL, Phylome, TreeFam
Q7KMQ0	35701	FBgn0028687	Rpt1	5705	9552	PSMC5	1	1.001	low	No	No	OrthoDB
Q7KMQ0	35701	FBgn0028687	Rpt1	5706	9553	PSMC6	1	1.001	low	No	No	OrthoDB
Q7KMQ0	35701	FBgn0028687	Rpt1	55759	14098	WDR12	1	1.001	low	No	No	OrthoDB
O18680	37815	FBgn0020764	Alas	212	397	ALAS2	10	9.669	high	Yes	Yes	Compara, Homologene, Inparanoid, Isobase, OMA, OrthoDB, orthoMCL, Phylome, RoundUp, TreeFar
O18680	37815	FBgn0020764	Alas	211	396	ALAS1	8	7.719	moderate	No	Yes	Compara, Inparanoid, OMA, OrthoDB, orthoMCL, Phylome, RoundUp, TreeFan
O18680	37815	FBgn0020764	Alas	23464	4188	GCAT	3	2.904	low	No	No	OrthoDB, orthoMCL, RoundUp
Q9VSP9	39005	FBgn0035926	CG5804	414149	17715	ACBD7	2	1.901	moderate	Yes	No	OrthoDB, orthoMCL
Q9VSP9	39005	FBgn0035926	CG5804	10455	14601	ECI2	1	1.001	low	No	No	OrthoDB
Q9VSP9	39005	FBgn0035926	CG5804	9085	1809	CDY1	1	1.001	low	No	No	OrthoDB
Q9VSP9	39005	FBgn0035926	CG5804	84320	23339	ACBD6	1	1.001	low	No	No	OrthoDB
Q9VSP9	39005	FBgn0035926	CG5804	253175	23920	CDY1B	1	1.001	low	No	No	OrthoDB
Q9VSP9	39005	FBgn0035926	CG5804	1622	2690	DBI	1	1.001	low	No	No	OrthoDB
Q9VSP9	39005	FBgn0035926	CG5804	9425	1811	CDYL	1	1.001	low	No	No	OrthoDB
P51592	41181	FBgn0002431	hyd	51366	16806	UBR5	10	9.669	high	Yes	Yes	Compara, Homologene, Inparanoid, Isobase, OMA, OrthoDB, orthoMCL, Phylome, RoundUp, TreeFar
Q95083	36951	FBgn0016697	Prosalpha5	5686	9534	PSMA5	9	8.719	high	Yes	Yes	Compara, Homologene, Inparanoid, OMA, OrthoDB, orthoMCL, Phylome, RoundUp, TreeFar
Q95083	36951	FBgn0016697	Prosalpha5	143471	22985	PSMA8	1	1.001	low	No	No	OrthoDB
Q95083	36951	FBgn0016697	Prosalpha5	5688	9536	PSMA7	1	1.001	low	No	No	OrthoDB
A8JV19	32672	FBgn0030796	CG4829	2678	4250	GGT1	7	6.704	high	Yes	Yes	Compara, Homologene, OrthoDB, orthoMCL, Phylome, RoundUp, TreeFar
A8JV19	32672	FBgn0030796	CG4829	728441	4251	GGT2	5	4.701	moderate	No	No	Compara, OrthoDB, orthoMCL, Phylome, TreeFam
A8JV19	32672	FBgn0030796	CG4829	2687	4260	GGT5	4	3.801	moderate	No	Yes	Compara, OrthoDB, Phylome, TreeFam
A8JV19	32672	FBgn0030796	CG4829	91227	18596	GGTLC2	2	1.931	low	No	No	Compara, OrthoDB
A8JV19	32672	FBgn0030796	CG4829	92086	16437	GGTLC1	2	1.931	low	No	No	Compara, OrthoDB
A8JV19	32672	FBgn0030796	CG4829	728226	33426	GGTLC3	1	0.91	low	No	No	Phylome
A8JV19	32672	FBgn0030796	CG4829	2686	4259	GGT7	1	0.9	low	No	Yes	orthoMCL
M9PEJ3	38971	FBgn0035903	CG6765	7709	12936	ZBTB17	1	0.91	low	Yes	Yes	Phylome
M9PEJ3	38971	FBgn0035903	CG6765	9880	29014	ZBTB39	1	0.91	low	Yes	No	Phylome
Q9VXE0	32636	FBgn0261791	SmG	6637	11163	SNRPG	9	8.719	high	Yes	Yes	Compara, Homologene, Inparanoid, OMA, OrthoDB, orthoMCL, Phylome, RoundUp, TreeFar
Q9VNA5	40639	FBgn0250746	Prosbeta7	5692	9541	PSMB4	10	9.669	high	Yes	Yes	Compara, Homologene, Inparanoid, Isobase, OMA, OrthoDB, orthoMCL, Phylome, RoundUp, TreeFar
Q9VD01	42644	FBgn0038973	Pebp1	5037	8630	PEBP1	3	2.891	moderate	Yes	No	Compara, OrthoDB, TreeFam
P55035	40388	FBgn0015283	Rpn10	5710	9561	PSMD4	10	9.669	high	Yes	Yes	Compara, Homologene, Inparanoid, Isobase, OMA, OrthoDB, orthoMCL, Phylome, RoundUp, TreeFar
P55035	40388	FBgn0015283	Rpn10	8394	8994	PIP5K1A	1	1.001	low	No	No	OrthoDB
P55035	40388	FBgn0015283	Rpn10	23396	8996	PIP5K1C	1	1.001	low	No	No	OrthoDB
P55035	40388	FBgn0015283	Rpn10	8395	8995	PIP5K1B	1	1.001	low	No	No	OrthoDB
P55035	40388	FBgn0015283	Rpn10	266971	23733	PIPSL	1	0.91	low	No	No	Phylome
E2QCS8	326153	FBgn0031390	tho2	57187	19073	THOC2	9	8.719	high	Yes	Yes	Compara, Homologene, Inparanoid, OMA, OrthoDB, orthoMCL, Phylome, RoundUp, TreeFar
Q7KN62	36040	FBgn0261014	TER94	7415	12666	VCP	9	8.739	high	Yes	Yes	Homologene, Inparanoid, Isobase, OMA, OrthoDB, orthoMCL, Phylome, RoundUp, TreeFan
Q7K3Z3	35674	FBgn0033179	p47	55968	15912	NSFL1C	9	8.719	high	Yes	Yes	Compara, Homologene, Inparanoid, OMA, OrthoDB, orthoMCL, Phylome, RoundUp, TreeFar
Q7K3Z3	35674	FBgn0033179	p47	137886	27035	UBXN2B	6	5.706	moderate	No	Yes	Compara, Inparanoid, OrthoDB, orthoMCL, Phylome, TreeFam

Q7K3Z3	35674	FBgn0033179	p47	165324	27265	UBXN2A	5	4.804	moderate	No	Yes	Compara, OrthoDB, Phylome, RoundUp, TreeFan
Q7K485	45268	FBgn0029093	cathD	1509	2529	CTSD	9	8.719	high	Yes	Yes	Compara, Homologene, Inparanoid, OMA, OrthoDB, orthoMCL, Phylome, RoundUp, TreeFar
Q7K485	45268	FBgn0029093	cathD	9476	13395	NAPSA	6	5.704	moderate	No	Yes	Compara, OrthoDB, orthoMCL, Phylome, RoundUp, TreeFan
Q7K485	45268	FBgn0029093	cathD	5972	9958	REN	4	3.791	moderate	No	No	Compara, OrthoDB, orthoMCL, TreeFam
Q7K485	45268	FBgn0029093	cathD	1510	2530	CTSE	3	2.851	low	No	No	Isobase, OrthoDB, orthoMCL
Q7K485	45268	FBgn0029093	cathD	5225	8890	PGC	1	1.001	low	No	No	OrthoDB
Q7K485	45268	FBgn0029093	cathD	5222	8887	PGA5	1	1.001	low	No	No	OrthoDB
Q7K485	45268	FBgn0029093	cathD	643834	8885	PGA3	1	1.001	low	No	No	OrthoDB
Q9VZ35	32040	FBgn0030262	Vago				0	0	None			
Q9VFZ4	41610	FBgn0038115	CG7966	8991	10719	SELENBP1	9	8.719	high	Yes	Yes	Compara, Homologene, Inparanoid, OMA, OrthoDB, orthoMCL, Phylome, RoundUp, TreeFar
Q95U30	40905	FBgn0261393	alpha-Est5				0	0	None			
M9PBS9	38750	FBgn0035715	CG10103	9798	28977	IST1	9	8.719	high	Yes	Yes	Compara, Homologene, Inparanoid, OMA, OrthoDB, orthoMCL, Phylome, RoundUp, TreeFar
P07909	43385	FBgn0001215	Hrb98DE	3181	5033	HNRNPA2B1	9	8.669	high	Yes	Yes	Compara, Inparanoid, Isobase, OMA, OrthoDB, orthoMCL, Phylome, RoundUp, TreeFam
P07909	43385	FBgn0001215	Hrb98DE	220988	24941	HNRNPA3	6	5.756	moderate	No	Yes	Compara, Inparanoid, OMA, OrthoDB, orthoMCL, Phylome
P07909	43385	FBgn0001215	Hrb98DE	144983	27067	HNRNPA1L2	5	4.856	moderate	No	Yes	Compara, Inparanoid, OMA, OrthoDB, Phylome
P07909	43385	FBgn0001215	Hrb98DE	3178	5031	HNRNPA1	5	4.746	moderate	No	Yes	Compara, Inparanoid, OrthoDB, orthoMCL, Phylome
P07909	43385	FBgn0001215	Hrb98DE	10949	5030	HNRNPA0	4	3.801	moderate	No	Yes	Compara, OrthoDB, Phylome, TreeFam
P07909	43385	FBgn0001215	Hrb98DE	26528	2683	DAZAP1	2	1.901	low	No	No	OrthoDB, orthoMCL
P07909	43385	FBgn0001215	Hrb98DE	124540	18585	MSI2	1	1.001	low	No	No	OrthoDB
P07909	43385	FBgn0001215	Hrb98DE	3182	5034	HNRNPAB	1	1.001	low	No	No	OrthoDB
P07909	43385	FBgn0001215	Hrb98DE	3184	5036	HNRNPD	1	1.001	low	No	No	OrthoDB
P07909	43385	FBgn0001215	Hrb98DE	9987	5037	HNRNPDL	1	1.001	low	No	No	OrthoDB
P07909	43385	FBgn0001215	Hrb98DE	4440	7330	MSI1	1	1.001	low	No	No	OrthoDB
Q9VMF0	33885	FBgn0031801	CG9498				0	0	None			
P11997	38015	FBgn0002564	Lsp1gamma				0	0	None			
Q9Y166	41640	FBgn0027610	Dic1	1468	10980	SLC25A10	7	6.738	high	Yes	Yes	Inparanoid, Isobase, OMA, orthoMCL, Phylome, RoundUp, TreeFam
Q9Y166	41640	FBgn0027610	Dic1	9016	10984	SLC25A14	2	2.004	low	No	No	OrthoDB, RoundUp
Q9Y166	41640	FBgn0027610	Dic1	8402	10981	SLC25A11	2	2.004	low	No	No	OrthoDB, RoundUp
Q9Y166	41640	FBgn0027610	Dic1	253512	27371	SLC25A30	2	2.004	low	No	No	OrthoDB, RoundUp
Q9Y166	41640	FBgn0027610	Dic1	6182	10378	MRPL12	2	1.94	low	No	No	Compara, OMA
Q9Y166	41640	FBgn0027610	Dic1	284723	27653	SLC25A34	1	1.003	low	No	No	RoundUp
Q9Y166	41640	FBgn0027610	Dic1	399512	31921	SLC25A35	1	1.003	low	No	No	RoundUp
Q9Y166	41640	FBgn0027610	Dic1	7351	12518	UCP2	1	1.001	low	No	Yes	OrthoDB
Q9Y166	41640	FBgn0027610	Dic1	9481	21065	SLC25A27	1	1.001	low	No	No	OrthoDB
Q9Y166	41640	FBgn0027610	Dic1	7350	12517	UCP1	1	1.001	low	No	Yes	OrthoDB
Q9Y166	41640	FBgn0027610	Dic1	7352	12519	UCP3	1	1.001	low	No	Yes	OrthoDB
P22769	32584	FBgn0004066	Prosalpha4	143471	22985	PSMA8	9	8.719	high	Yes	Yes	Compara, Homologene, Inparanoid, OMA, OrthoDB, orthoMCL, Phylome, RoundUp, TreeFar
P22769	32584	FBgn0004066	Prosalpha4	5688	9536	PSMA7	9	8.669	high	Yes	Yes	Compara, Inparanoid, Isobase, OMA, OrthoDB, orthoMCL, Phylome, RoundUp, TreeFam
P22769	32584	FBgn0004066	Prosalpha4	5685	9533	PSMA4	1	1.01	low	No	No	OMA
P22769	32584	FBgn0004066	Prosalpha4	5686	9534	PSMA5	1	1.001	low	No	No	OrthoDB
Q8IMH4	43662	FBgn0000416	Sap-r	5660	9498	PSAP	8	7.709	high	Yes	Yes	Compara, Homologene, Inparanoid, OrthoDB, orthoMCL, Phylome, RoundUp, TreeFar
Q8IMH4	43662	FBgn0000416	Sap-r	768239	33131	PSAPL1	5	4.806	moderate	No	Yes	Compara, Inparanoid, OrthoDB, Phylome, TreeFam
Q8IMH4	43662	FBgn0000416	Sap-r	6439	10801	SFTPB	4	3.801	moderate	No	Yes	Compara, OrthoDB, Phylome, TreeFam
P12881	34359	FBgn0250843	Prosalpha6	5682	9530	PSMA1	8	7.719	moderate	Yes	No	Compara, Inparanoid, OMA, OrthoDB, orthoMCL, Phylome, RoundUp, TreeFan
A8JNT7	5740624	FBgn0263606	Hsc20	150274	28913	HSCB	9	8.719	high	Yes	Yes	Compara, Homologene, Inparanoid, OMA, OrthoDB, orthoMCL, Phylome, RoundUp, TreeFar
P25161	35176	FBgn0261396	Rpn3	5709	9560	PSMD3	8	7.819	high	Yes	Yes	Compara, Homologene, Inparanoid, OMA, OrthoDB, Phylome, RoundUp, TreeFan
Q9VH72	41197	FBgn0037749	CG9471	645	1063	BLVRB	10	9.669	high	Yes	Yes	Compara, Homologene, Inparanoid, Isobase, OMA, OrthoDB, orthoMCL, Phylome, RoundUp, TreeFar
Q8IRD3	38413	FBgn0035438	PHGPx	2879	4556	GPX4	6	5.749	high	Yes	Yes	Compara, Inparanoid, OrthoDB, orthoMCL, Phylome, RoundUp
Q8IRD3	38413	FBgn0035438	PHGPx	2882	4559	GPX7	4	3.864	moderate	No	Yes	OrthoDB, orthoMCL, RoundUp, TreeFam
Q8IRD3	38413	FBgn0035438	PHGPx	493869	33100	GPX8	4	3.864	moderate	No	Yes	OrthoDB, orthoMCL, RoundUp, TreeFam
Q7K148	45269	FBgn0029134	Prosbeta5	5693	9542	PSMB5	10	9.669	high	Yes	Yes	Compara, Homologene, Inparanoid, Isobase, OMA, OrthoDB, orthoMCL, Phylome, RoundUp, TreeFar
Q7K148	45269	FBgn0029134	Prosbeta5	5696	9545	PSMB8	7	6.709	moderate	No	Yes	Compara, Inparanoid, OrthoDB, orthoMCL, Phylome, RoundUp, TreeFan
Q7K148	45269	FBgn0029134	Prosbeta5	122706	31963	PSMB11	4	3.801	moderate	No	No	Compara, OrthoDB, Phylome, TreeFam
Q7KSM5	41532	FBgn0029512	Aos1	10055	30660	SAE1	9	8.719	high	Yes	Yes	Compara, Homologene, Inparanoid, OMA, OrthoDB, orthoMCL, Phylome, RoundUp, TreeFar
Q7KSM5	41532	FBgn0029512	Aos1	8883	621	NAE1	1	1.003	low	No	No	RoundUp
Q7K3B7	37285	FBgn0034488	CG11208	26061	17856	HACL1	10	9.669	high	Yes	Yes	Compara, Homologene, Inparanoid, Isobase, OMA, OrthoDB, orthoMCL, Phylome, RoundUp, TreeFar
Q7K3B7	37285	FBgn0034488	CG11208	10994	6041	ILVBL	1	0.9	low	No	Yes	orthoMCL
Q7KSK2	41717	FBgn0038205	Kif19A	124602	26735	KIF19	6	5.919	high	Yes	Yes	Homologene, Inparanoid, Isobase, OrthoDB, RoundUp, TreeFam
Q7KSK2	41717	FBgn0038205	Kif19A	81930	29441	KIF18A	2	1.903	low	No	No	orthoMCL, RoundUp
Q7KSK2	41717	FBgn0038205	Kif19A	350383	20088	GPR142	1	0.93	low	No	Yes	Compara
Q7KSK2	41717	FBgn0038205	Kif19A	146909	27102	KIF18B	1	0.9	low	No	No	orthoMCL
A1Z9F9	36511	FBgn0261989	CG42807				0	0	None			
M9PCP3	33825	FBgn0031752	CG9044	114790	19184	STK11IP	7	6.709	high	Yes	Yes	Compara, Inparanoid, OrthoDB, orthoMCL, Phylome, RoundUp, TreeFan
M9PCP3	33825	FBgn0031752	CG9044	11188	18006	NISCH	1	1.003	low	No	No	RoundUp
Q8IN44	44121	FBgn0028396	TotA				0	0	None			
Q86BR8	42433	FBgn0038805	TFAM	7019	11741	TFAM	9	8.719	high	Yes	Yes	Compara, Homologene, Inparanoid, OMA, OrthoDB, orthoMCL, Phylome, RoundUp, TreeFar
Q24524	31717	FBgn0003447	sn	6624	11148	FSCN1	8	7.769	high	Yes	Yes	Compara, Inparanoid, Isobase, OMA, OrthoDB, Phylome, RoundUp, TreeFan
Q24524	31717	FBgn0003447	sn	25794	3960	FSCN2	7	6.809	moderate	No	Yes	Compara, Homologene, Inparanoid, OrthoDB, Phylome, RoundUp, TreeFar
Q24524	31717	FBgn0003447	sn	29999	3961	FSCN3	2	1.931	moderate	No	Yes	Compara, OrthoDB
Q9VSL3	38973	FBgn0086348	se	9446	13312	GSTO1	10	9.669	high	Yes	Yes	Compara, Homologene, Inparanoid, Isobase, OMA, OrthoDB, orthoMCL, Phylome, RoundUp, TreeFar
Q9VSL3	38973	FBgn0086348	se	119391	23064	GSTO2	7	6.819	moderate	No	No	Compara, Inparanoid, OMA, OrthoDB, Phylome, RoundUp, TreeFan
Q7KV99	38286	FBgn0035321	CG1275	79901	20797	CYBRD1	9	8.719	high	Yes	Yes	Compara, Homologene, Inparanoid, OMA, OrthoDB, orthoMCL, Phylome, RoundUp, TreeFar
Q7KV99	38286	FBgn0035321	CG1275	1534	2571	CYB561	6	5.653	moderate	No	Yes	Compara, Isobase, orthoMCL, Phylome, RoundUp, TreeFan
Q7KV99	38286	FBgn0035321	CG1275	220002	23014	CYB561A3	3	2.8	moderate	No	Yes	Compara, Phylome, TreeFam
Q7KV99	38286	FBgn0035321	CG1275	284613	26804	CYB561D1	1	0.91	low	No	No	Phylome
Q7KV99	38286	FBgn0035321	CG1275	11068	30253	CYB561D2	1	0.91	low	No	No	Phylome
Q9VVL5	39986	FBgn0036760	CG5567	283871	8909	PGP	10	9.669	high	Yes	Yes	Compara, Homologene, Inparanoid, Isobase, OMA, OrthoDB, orthoMCL, Phylome, RoundUp, TreeFar
Q9VVL5	39986	FBgn0036760	CG5567	57026	30259	PDXP	6	5.799	moderate	No	Yes	Compara, Inparanoid, OrthoDB, orthoMCL, RoundUp, TreeFan
Q9VVL5	39986	FBgn0036760	CG5567	2631	4179	GBAS	1	1.003	low	No	No	RoundUp
Q9VVL5	39986	FBgn0036760	CG5567	8508	7827	NIPSNAP1	1	1.003	low	No	No	RoundUp
A1Z8F4	36171	FBgn0003396	shn	3097	4921	HIVEP2	6	5.758	high	Yes	Yes	Compara, Inparanoid, Isobase, Phylome, RoundUp, TreeFan
A1Z8F4	36171	FBgn0003396	shn	59269	13561	HIVEP3	5	4.808	moderate	No	Yes	Compara, Inparanoid, Phylome, RoundUp, TreeFan
A1Z8F4	36171	FBgn0003396	shn	3096	4920	HIVEP1	5	4.808	moderate	No	Yes	Compara, Inparanoid, Phylome, RoundUp, TreeFan
A1Z8F4	36171	FBgn0003396	shn	128611	16167	ZNF831	2	1.84	moderate	No	Yes	Compara, Phylome
Q9VJY6	34754	FBgn0032518	RpL24	6152	10325	RPL24	8	7.709	high	Yes	Yes	Compara, Homologene, Inparanoid, OrthoDB, orthoMCL, Phylome, RoundUp, TreeFar
Q9VH39	41227	FBgn0037777	CG11722	29078	21034	NDUFAF4	9	8.719	high	Yes	Yes	Compara, Homologene, Inparanoid, OMA, OrthoDB, orthoMCL, Phylome, RoundUp, TreeFar
Q9VGT0	41333	FBgn0026314	Ugt35b	79799	28528	UGT2A3	5	4.818	high	Yes	Yes	Homologene, Inparanoid, orthoMCL, Phylome, RoundU
Q9VGT0	41333	FBgn0026314	Ugt35b	7366	12546	UGT2B15	5	4.768	high	Yes	Yes	Inparanoid, Isobase, orthoMCL, Phylome, RoundUp
Q9VGT0	41333	FBgn0026314	Ugt35b	54490	13479	UGT2B28	4	3.866	moderate	No	Yes	Inparanoid, Isobase, OrthoDB, Phylome
Q9VGT0	41333	FBgn0026314	Ugt35b	10720	12545	UGT2B11	4	3.816	moderate	No	No	Inparanoid, OrthoDB, orthoMCL, Phylome
Q9VGT0	41333	FBgn0026314	Ugt35b	7367	12547	UGT2B17	4	3.765	moderate	No	Yes	Inparanoid, Isobase, orthoMCL, Phylome
Q9VGT0	41333	FBgn0026314	Ugt35b	7363	12553	UGT2B4	3	2.815	moderate	No	Yes	Inparanoid, orthoMCL, Phylome
Q9VGT0	41333	FBgn0026314	Ugt35b	574537	28183	UGT2A2	3	2.815	low	No	No	Inparanoid, orthoMCL, Phylome

Q9VGT0	41333	FBgn0026314	Ugt35b	7364	12554	UGT2B7	3	2.815	low	No	No	Inparanoid, orthoMCL, Phylome
Q9VGT0	41333	FBgn0026314	Ugt35b	7368	12555	UGT8	3	2.74	low	No	No	Compara, orthoMCL, Phylome
Q9VGT0	41333	FBgn0026314	Ugt35b	54659	12535	UGT1A3	2	2.006	low	No	No	Inparanoid, OrthoDB
Q9VGT0	41333	FBgn0026314	Ugt35b	54578	12538	UGT1A6	2	1.915	low	No	No	Inparanoid, Phylome
Q9VGT0	41333	FBgn0026314	Ugt35b	7365	12544	UGT2B10	2	1.905	low	No	No	Inparanoid, orthoMCL
Q9VGT0	41333	FBgn0026314	Ugt35b	133688	26625	UGT3A1	2	1.84	moderate	No	Yes	Compara, Phylome
Q9VGT0	41333	FBgn0026314	Ugt35b	167127	27266	UGT3A2	2	1.84	low	No	No	Compara, Phylome
Q9VGT0	41333	FBgn0026314	Ugt35b	54658	12530	UGT1A1	1	1.005	low	No	No	Inparanoid
Q9VGT0	41333	FBgn0026314	Ugt35b	54657	12536	UGT1A4	1	1.005	low	No	Yes	Inparanoid
Q9VGT0	41333	FBgn0026314	Ugt35b	54575	12531	UGT1A10	1	1.005	low	No	No	Inparanoid
Q9VGT0	41333	FBgn0026314	Ugt35b	54577	12539	UGT1A7	1	1.005	low	No	No	Inparanoid
Q9VGT0	41333	FBgn0026314	Ugt35b	54579	12537	UGT1A5	1	1.005	low	No	Yes	Inparanoid
Q9VGT0	41333	FBgn0026314	Ugt35b	54600	12541	UGT1A9	1	1.005	low	No	Yes	Inparanoid
Q9VGT0	41333	FBgn0026314	Ugt35b	54576	12540	UGT1A8	1	1.005	low	No	Yes	Inparanoid
Q9VP61	40348	FBgn0012034	AcCoAS	55902	15814	ACSS2	8	7.709	high	Yes	Yes	Compara, Homologene, Inparanoid, OrthoDB, orthoMCL, Phylome, RoundUp, TreeFam
Q9VP61	40348	FBgn0012034	AcCoAS	84532	16091	ACSS1	4	3.854	moderate	No	Yes	Isobase, OrthoDB, orthoMCL, RoundUp
Q9VP61	40348	FBgn0012034	AcCoAS	79611	24723	ACSS3	1	0.9	low	No	No	orthoMCL
Q967S0	38753	FBgn0041194	Prat2	5471	9238	PPAT	10	9.669	high	Yes	Yes	Compara, Homologene, Inparanoid, Isobase, OMA, OrthoDB, orthoMCL, Phylome, RoundUp, TreeFam
P54192	33040	FBgn0011280	Obp19d				0	0	None			
Q9VA83	44965	FBgn0015221	Fer2LCH	94033	17345	FTMT	1	0.95	low	Yes	No	Isobase
Q9VCI3	42810	FBgn0039114	Lsd-1	10226	16893	PLIN3	3	2.918	high	Yes	Yes	Inparanoid, Phylome, RoundUp
Q9VCI3	42810	FBgn0039114	Lsd-1	5346	9076	PLIN1	3	2.873	moderate	Yes	No	Phylome, RoundUp, TreeFam
Q9VCI3	42810	FBgn0039114	Lsd-1	440503	33196	PLIN5	2	1.915	moderate	No	Yes	Inparanoid, Phylome
Q9VCI3	42810	FBgn0039114	Lsd-1	123	248	PLIN2	2	1.913	low	No	No	Phylome, RoundUp
Q7KUQ6	39889	FBgn0261565	Lmpt	2274	3703	FHL2	5	4.873	high	Yes	Yes	Homologene, Isobase, OMA, Phylome, RoundUp
Q7KUQ6	39889	FBgn0261565	Lmpt	2275	3704	FHL3	1	0.91	low	No	No	Phylome
Q7KUQ6	39889	FBgn0261565	Lmpt	2273	3702	FHL1	1	0.91	low	No	No	Phylome
Q7KUQ6	39889	FBgn0261565	Lmpt	1758	2932	DMP1	1	0.91	low	No	Yes	Phylome
Q7KUQ6	39889	FBgn0261565	Lmpt	9457	17371	FHL5	1	0.91	low	No	No	Phylome
O97479	40836	FBgn0024289	Sodh-1	6652	11184	SORD	10	9.669	high	Yes	Yes	Compara, Homologene, Inparanoid, Isobase, OMA, OrthoDB, orthoMCL, Phylome, RoundUp, TreeFam
P08171	39392	FBgn0000592	Est-6				0	0	None			
Q9VQL7	33524	FBgn0027571	CG3523	2194	3594	FASN	8	7.659	high	Yes	Yes	Compara, Inparanoid, Isobase, OrthoDB, orthoMCL, Phylome, RoundUp, TreeFam
P35500	32619	FBgn0264255	para	6326	10588	SCN2A	8	7.759	high	Yes	Yes	Compara, Homologene, Inparanoid, OMA, OrthoDB, orthoMCL, Phylome, RoundU
P35500	32619	FBgn0264255	para	6323	10585	SCN1A	7	6.759	moderate	No	Yes	Compara, Inparanoid, OMA, OrthoDB, orthoMCL, Phylome, RoundUp
P35500	32619	FBgn0264255	para	6334	10596	SCN8A	7	6.699	moderate	No	Yes	Compara, Inparanoid, Isobase, OrthoDB, orthoMCL, Phylome, RoundUp
P35500	32619	FBgn0264255	para	6331	10593	SCN5A	6	5.706	moderate	No	Yes	Compara, Inparanoid, OrthoDB, orthoMCL, Phylome, TreeFam
P35500	32619	FBgn0264255	para	6332	10594	SCN7A	6	5.706	moderate	No	Yes	Compara, Inparanoid, OrthoDB, orthoMCL, Phylome, TreeFam
P35500	32619	FBgn0264255	para	6336	10582	SCN10A	6	5.706	moderate	No	Yes	Compara, Inparanoid, OrthoDB, orthoMCL, Phylome, TreeFam
P35500	32619	FBgn0264255	para	6329	10591	SCN4A	5	4.746	moderate	No	Yes	Compara, Inparanoid, OrthoDB, orthoMCL, Phylome
P35500	32619	FBgn0264255	para	6328	10590	SCN3A	5	4.746	moderate	No	Yes	Compara, Inparanoid, OrthoDB, orthoMCL, Phylome
P35500	32619	FBgn0264255	para	11280	10583	SCN11A	5	4.701	moderate	No	Yes	Compara, OrthoDB, orthoMCL, Phylome, TreeFam
P35500	32619	FBgn0264255	para	6335	10597	SCN9A	4	3.836	moderate	No	Yes	Compara, Inparanoid, OrthoDB, orthoMCL
P35500	32619	FBgn0264255	para	775	1390	CACNA1C	1	1.003	low	No	No	RoundUp
P35500	32619	FBgn0264255	para	773	1388	CACNA1A	1	1.003	low	No	No	RoundUp
P35500	32619	FBgn0264255	para	259232	19082	NALCN	1	1.003	low	No	No	RoundUp
P35500	32619	FBgn0264255	para	779	1397	CACNA1S	1	1.003	low	No	No	RoundUp
P35500	32619	FBgn0264255	para	776	1391	CACNA1D	1	1.003	low	No	No	RoundUp
Q04691	39566	FBgn0000639	Fbp1				0	0	None			
Q9XYZ9	37960	FBgn0027590	GstE12	2940	4628	GSTA3	1	0.91	low	Yes	Yes	Phylome
Q9XYZ9	37960	FBgn0027590	GstE12	2941	4629	GSTA4	1	0.91	low	Yes	Yes	Phylome
Q9XYZ9	37960	FBgn0027590	GstE12	2939	4627	GSTA2	1	0.91	low	Yes	Yes	Phylome
Q9XYZ9	37960	FBgn0027590	GstE12	2938	4626	GSTA1	1	0.91	low	Yes	Yes	Phylome
Q9VA31	43658	FBgn0039806	CG15545				0	0	None			
Q9V3S0	30986	FBgn0010019	Cyp4g1	1579	2642	CYP4A11	1	1.001	low	Yes	No	OrthoDB
Q9V3S0	30986	FBgn0010019	Cyp4g1	260293	20244	CYP4X1	1	1.001	low	Yes	No	OrthoDB
Q9V3S0	30986	FBgn0010019	Cyp4g1	8529	2645	CYP4F2	1	1.001	low	Yes	No	OrthoDB
Q9V3S0	30986	FBgn0010019	Cyp4g1	57834	13265	CYP4F11	1	1.001	low	Yes	No	OrthoDB
Q9V3S0	30986	FBgn0010019	Cyp4g1	285440	23198	CYP4V2	1	1.001	low	Yes	No	OrthoDB
Q9V3S0	30986	FBgn0010019	Cyp4g1	66002	18857	CYP4F12	1	1.001	low	Yes	No	OrthoDB
Q9V3S0	30986	FBgn0010019	Cyp4g1	1580	2644	CYP4B1	1	1.001	low	Yes	No	OrthoDB
Q9V3S0	30986	FBgn0010019	Cyp4g1	284541	20575	CYP4A22	1	1.001	low	Yes	No	OrthoDB
Q9V3S0	30986	FBgn0010019	Cyp4g1	1582	2653	CYP8B1	1	0.95	low	Yes	Yes	Isobase
Q9W5B4	31055	FBgn0040398	CG14629	3475	5456	IFRD1	1	1.001	low	Yes	No	OrthoDB
Q9W5B4	31055	FBgn0040398	CG14629	7866	5457	IFRD2	1	1.001	low	Yes	No	OrthoDB
Q9VXG9	32606	FBgn0030745	CG4239	79041	28462	TMEM38A	8	7.668	high	Yes	Yes	Compara, Inparanoid, Isobase, OMA, orthoMCL, Phylome, RoundUp, TreeFam
Q9VXG9	32606	FBgn0030745	CG4239	55151	25535	TMEM38B	7	6.718	moderate	No	Yes	Compara, Inparanoid, OMA, orthoMCL, Phylome, RoundUp, TreeFam
P54353	33111	FBgn0015379	dod	5300	8988	PIN1	9	8.668	high	Yes	Yes	Compara, Homologene, Inparanoid, Isobase, OMA, orthoMCL, Phylome, RoundUp, TreeFam
Q9VLS9	34106	FBgn0031987	CG12375	51110	18512	LACTB2	8	7.759	high	Yes	Yes	Compara, Homologene, Inparanoid, OMA, OrthoDB, orthoMCL, Phylome, RoundU
Q9VF15	41930	FBgn0027657	glob1	114757	16505	CYGB	5	4.768	high	Yes	Yes	Inparanoid, Isobase, orthoMCL, Phylome, RoundUp
Q9VF15	41930	FBgn0027657	glob1	4151	6915	MB	2	1.915	moderate	No	Yes	Inparanoid, Phylome
Q9VF15	41930	FBgn0027657	glob1	3043	4827	HBB	1	1.005	low	No	Yes	Inparanoid
Q9VF15	41930	FBgn0027657	glob1	3048	4832	HBG2	1	1.005	low	No	Yes	Inparanoid
Q9VF15	41930	FBgn0027657	glob1	3040	4824	HBA2	1	1.005	low	No	Yes	Inparanoid
Q9VF15	41930	FBgn0027657	glob1	3046	4830	HBE1	1	1.005	low	No	Yes	Inparanoid
Q9VF15	41930	FBgn0027657	glob1	3045	4829	HBD	1	1.005	low	No	Yes	Inparanoid
Q9VF15	41930	FBgn0027657	glob1	3050	4835	HBZ	1	1.005	low	No	Yes	Inparanoid
Q9VF15	41930	FBgn0027657	glob1	3047	4831	HBG1	1	1.005	low	No	Yes	Inparanoid
Q9VF15	41930	FBgn0027657	glob1	3049	4833	HBQ1	1	1.005	low	No	Yes	Inparanoid
Q9VF15	41930	FBgn0027657	glob1	58157	14077	NGB	1	0.91	low	No	Yes	Phylome
Q7KRU8	46415	FBgn0015222	Fer1HCH	2495	3976	FTH1	1	0.93	low	Yes	No	Compara
Q7KRU8	46415	FBgn0015222	Fer1HCH	2512	3999	FTL	1	0.93	low	Yes	No	Compara
Q7KRU8	46415	FBgn0015222	Fer1HCH	53940	3987	FTHL17	1	0.93	low	Yes	No	Compara
Q7KRU8	46415	FBgn0015222	Fer1HCH	94033	17345	FTMT	1	0.93	low	Yes	No	Compara
Q9W306	31923	FBgn0030160	CG9691				0	0	None			
Q9VRJ1	38595	FBgn0028962	Aats-ala-m	57505	21022	AARS2	3	2.79	high	Yes	Yes	Compara, orthoMCL, TreeFam
Q9VRJ1	38595	FBgn0028962	Aats-ala-m	16	20	AARS	2	1.85	low	No	No	Isobase, orthoMCL
P62152	36329	FBgn0000253	Cam	801	1442	CALM1	7	6.754	high	Yes	Yes	Compara, Isobase, OMA, OrthoDB, orthoMCL, RoundUp, TreeFam
P62152	36329	FBgn0000253	Cam	808	1449	CALM3	7	6.718	high	Yes	Yes	Compara, Inparanoid, OMA, orthoMCL, Phylome, RoundUp, TreeFam
P62152	36329	FBgn0000253	Cam	810	1452	CALML3	4	3.864	moderate	No	Yes	OrthoDB, orthoMCL, RoundUp, TreeFam
P62152	36329	FBgn0000253	Cam	805	1445	CALM2	2	1.97	moderate	No	Yes	OMA, TreeFam
P62152	36329	FBgn0000253	Cam	285051	26850	C2orf61	2	1.94	moderate	No	Yes	Compara, OMA
P62152	36329	FBgn0000253	Cam	1069	1867	CETN2	1	1.001	low	No	No	OrthoDB
P62152	36329	FBgn0000253	Cam	51806	18180	CALML5	1	1.001	low	No	No	OrthoDB

P62152	36329	FBgn0000253	Cam	1070	1868	CETN3	1	1.001	low	No	No	OrthoDB
P62152	36329	FBgn0000253	Cam	1068	1866	CETN1	1	1.001	low	No	No	OrthoDB
P02843	31939	FBgn0004045	Yp1	9388	6623	LIPG	1	0.93	low	Yes	No	Compara
P02843	31939	FBgn0004045	Yp1	4023	6677	LPL	1	0.93	low	Yes	No	Compara
P02843	31939	FBgn0004045	Yp1	3990	6619	LIPC	1	0.93	low	Yes	No	Compara
P06607	32339	FBgn0004047	Yp3	9388	6623	LIPG	1	0.93	low	Yes	No	Compara
P06607	32339	FBgn0004047	Yp3	4023	6677	LPL	1	0.93	low	Yes	No	Compara
P06607	32339	FBgn0004047	Yp3	3990	6619	LIPC	1	0.93	low	Yes	No	Compara
Q6NR72	37739	FBgn0034885	CG4019	363	639	AQP6	2	1.951	high	Yes	Yes	Isobase, OrthoDB
Q6NR72	37739	FBgn0034885	CG4019	361	637	AQP4	2	1.901	moderate	Yes	No	OrthoDB, orthoMCL
Q6NR72	37739	FBgn0034885	CG4019	362	638	AQP5	2	1.901	moderate	Yes	No	OrthoDB, orthoMCL
Q6NR72	37739	FBgn0034885	CG4019	89872	16029	AQP10	2	1.81	high	Yes	Yes	orthoMCL, Phylome
Q6NR72	37739	FBgn0034885	CG4019	360	636	AQP3	2	1.81	moderate	Yes	No	orthoMCL, Phylome
Q6NR72	37739	FBgn0034885	CG4019	366	643	AQP9	2	1.81	high	Yes	Yes	orthoMCL, Phylome
Q6NR72	37739	FBgn0034885	CG4019	364	640	AQP7	2	1.81	high	Yes	Yes	orthoMCL, Phylome
Q6NR72	37739	FBgn0034885	CG4019	359	634	AQP2	1	1.001	low	No	No	OrthoDB
Q6NR72	37739	FBgn0034885	CG4019	4284	7103	MIP	1	1.001	low	No	No	OrthoDB
Q6NR72	37739	FBgn0034885	CG4019	343	642	AQP8	1	1.001	low	No	No	OrthoDB
Q9I7Q5	59158	FBgn0042119	Cpr65Au				0	0	None			
Q9W380	50405	FBgn0040931	CG9034				0	0	None			
Q95U34	39031	FBgn0263199	Galk	2585	4119	GALK2	10	9.669	high	Yes	Yes	Compara, Homologene, Inparanoid, Isobase, OMA, OrthoDB, orthoMCL, Phylome, RoundUp, TreeFam
P07668	42249	FBgn0000303	Cha	1103	1912	CHAT	9	8.659	high	Yes	Yes	Compara, Homologene, Inparanoid, Isobase, OrthoDB, orthoMCL, Phylome, RoundUp, TreeFam
P07668	42249	FBgn0000303	Cha	1384	2342	CRAT	2	2.004	low	No	No	OrthoDB, RoundUp
C9QPE7	34016	FBgn0031907	CG5171	286451	28304	YIPF6	1	1.001	low	Yes	No	OrthoDB
Q8INW5	35222	FBgn0032783	CG10237	79183	16114	TTPAL	4	3.918	high	Yes	Yes	Inparanoid, OMA, orthoMCL, RoundUp
Q8INW5	35222	FBgn0032783	CG10237	134829	23046	CLVS2	2	1.913	moderate	No	Yes	Phylome, RoundUp
Q8INW5	35222	FBgn0032783	CG10237	157807	23139	CLVS1	1	0.91	low	No	No	Phylome
Q8INW5	35222	FBgn0032783	CG10237	6017	10024	RLBP1	1	0.91	low	No	No	Phylome
Q7JYW9	44008	FBgn0001187	Hex-C	2645	4195	GCK	7	6.656	moderate	Yes	No	Compara, Inparanoid, Isobase, OrthoDB, orthoMCL, Phylome, TreeFam
Q7JYW9	44008	FBgn0001187	Hex-C	3098	4922	HK1	5	4.741	moderate	No	No	Compara, Homologene, OrthoDB, orthoMCL, Phylome
Q7JYW9	44008	FBgn0001187	Hex-C	80201	23302	HKDC1	4	3.741	moderate	No	No	Compara, OrthoDB, orthoMCL, Phylome
Q7JYW9	44008	FBgn0001187	Hex-C	3101	4925	HK3	4	3.741	moderate	No	No	Compara, OrthoDB, orthoMCL, Phylome
Q7JYW9	44008	FBgn0001187	Hex-C	3099	4923	HK2	4	3.741	moderate	No	No	Compara, OrthoDB, orthoMCL, Phylome
Q9Y143	34728	FBgn0027586	CG5867				0	0	None			
A1Z7Z4	36009	FBgn0033446	CG1648				0	0	None			
O76454	43499	FBgn0024841	Pcd	5092	8646	PCBD1	8	7.654	high	Yes	Yes	Compara, Homologene, Isobase, OrthoDB, orthoMCL, Phylome, RoundUp, TreeFam
O76454	43499	FBgn0024841	Pcd	84105	24474	PCBD2	4	3.791	moderate	No	Yes	Compara, OrthoDB, orthoMCL, TreeFam
Q9VP02	40406	FBgn0043783	CG32444	130589	24063	GALM	6	5.696	moderate	Yes	No	Compara, Inparanoid, Isobase, OrthoDB, orthoMCL, Phylome
Q9VP02	40406	FBgn0043783	CG32444	5986	9974	RFNG	1	0.96	low	No	No	TreeFam
Q9VP02	40406	FBgn0043783	CG32444	3955	6560	LFNG	1	0.96	low	No	No	TreeFam
Q9VP02	40406	FBgn0043783	CG32444	4242	7038	MFNG	1	0.96	low	No	No	TreeFam
B7Z061	39812	FBgn0011693	Pdh	3248	5154	HPGD	9	8.669	high	Yes	Yes	Compara, Inparanoid, Isobase, OMA, OrthoDB, orthoMCL, Phylome, RoundUp, TreeFam
B7Z061	39812	FBgn0011693	Pdh	1666	2753	DECR1	1	1.001	low	No	Yes	OrthoDB
B7Z061	39812	FBgn0011693	Pdh	55825	18281	PECR	1	1.001	low	No	Yes	OrthoDB
B7Z061	39812	FBgn0011693	Pdh	26063	2754	DECR2	1	1.001	low	No	Yes	OrthoDB
B7Z061	39812	FBgn0011693	Pdh	56898	32389	BDH2	1	1.001	low	No	Yes	OrthoDB
B7Z061	39812	FBgn0011693	Pdh	51171	23238	HSD17B14	1	1.001	low	No	No	OrthoDB
Q29R16	44297	FBgn0259175	ome	2191	3590	FAP	6	5.753	high	Yes	Yes	Compara, Homologene, OMA, orthoMCL, Phylome, RoundUp
Q29R16	44297	FBgn0259175	ome	1803	3009	DPP4	5	4.753	moderate	No	No	Compara, OMA, orthoMCL, Phylome, RoundUp
Q29R16	44297	FBgn0259175	ome	1804	3010	DPP6	3	2.873	low	No	No	Phylome, RoundUp, TreeFam
Q29R16	44297	FBgn0259175	ome	57628	20823	DPP10	2	1.963	low	No	No	RoundUp, TreeFam
Q9VY98	32330	FBgn0030514	CG9941	23295	20254	MGRN1	8	7.659	high	Yes	Yes	Compara, Inparanoid, Isobase, OrthoDB, orthoMCL, Phylome, RoundUp, TreeFam
Q9VY98	32330	FBgn0030514	CG9941	114804	29402	RNF157	7	6.709	moderate	No	Yes	Compara, Inparanoid, OrthoDB, orthoMCL, Phylome, RoundUp, TreeFam
Q2MGJ8	3771756	FBgn0038033	CG10097	55711	25531	FAR2	2	1.931	moderate	Yes	No	Compara, OrthoDB
Q2MGJ8	3771756	FBgn0038033	CG10097	84188	26222	FAR1	2	1.931	moderate	Yes	No	Compara, OrthoDB
Q2MGJ8	3771756	FBgn0038033	CG10097	440905	49284	FAR2P1	1	0.91	low	No	Yes	Phylome
Q9W1C9	49821	FBgn0011695	PebIII				0	0	None			
Q9VIQ6	35281	FBgn0032835	CG16772				0	0	None			
Q9Y119	33642	FBgn0027560	Tps1				0	0	None			
Q7K483	43574	FBgn0039741	CG7943	92014	23323	SLC25A51	7	6.758	high	Yes	Yes	Compara, Homologene, Inparanoid, OMA, orthoMCL, Phylome, RoundUp
Q7K483	43574	FBgn0039741	CG7943	147407	23324	SLC25A52	6	5.859	moderate	No	Yes	Compara, Inparanoid, OMA, OrthoDB, Phylome, RoundUp
Q7K483	43574	FBgn0039741	CG7943	401612	31894	SLC25A53	4	3.801	moderate	No	Yes	Compara, OrthoDB, Phylome, TreeFam
Q7K483	43574	FBgn0039741	CG7943	494141	-	LOC494141	1	0.91	low	No	Yes	Phylome
Q8IQQ0	39899	FBgn0010352	Nc73EF	4967	8124	OGDH	9	8.659	high	Yes	Yes	Compara, Homologene, Inparanoid, Isobase, OrthoDB, orthoMCL, Phylome, RoundUp, TreeFam
Q8IQQ0	39899	FBgn0010352	Nc73EF	55753	25590	OGDHL	7	6.709	moderate	No	Yes	Compara, Inparanoid, OrthoDB, orthoMCL, Phylome, RoundUp, TreeFam
Q8IQQ0	39899	FBgn0010352	Nc73EF	55526	23537	DHTKD1	1	0.9	low	No	No	orthoMCL
M9NEN9	33361	FBgn0051665	wry	92737	24456	DNER	3	2.82	high	Yes	Yes	Isobase, Phylome, TreeFam
M9NEN9	33361	FBgn0051665	wry	65989	21113	DLK2	1	0.93	low	No	Yes	Compara
Q9W3W8	31613	FBgn0029897	RpL17	100526842	44661	RPL17-C18orf32	6	5.849	high	Yes	Yes	Compara, Homologene, Inparanoid, OrthoDB, Phylome, RoundUp
Q9W3W8	31613	FBgn0029897	RpL17	6139	10307	RPL17	5	4.801	moderate	No	Yes	Compara, OMA, OrthoDB, orthoMCL, TreeFam
Q9W3W8	31613	FBgn0029897	RpL17	51548	14934	SIRT6	1	1.001	low	No	No	OrthoDB
Q9W3W8	31613	FBgn0029897	RpL17	497661	31690	C18orf32	1	0.93	low	No	No	Compara
P22817	40248	FBgn0001247	Ide	3416	5381	IDE	9	8.719	high	Yes	Yes	Compara, Homologene, Inparanoid, OMA, OrthoDB, orthoMCL, Phylome, RoundUp, TreeFam
P22817	40248	FBgn0001247	Ide	4898	7995	NRD1	3	2.904	low	No	No	OrthoDB, orthoMCL, RoundUp
Q9W5E4	31010	FBgn0001341	l(1)1Bi	10514	7546	MYBBP1A	3	2.813	high	Yes	Yes	orthoMCL, Phylome, RoundUp
A1Z8H0	36186	FBgn0085256	CG34227				0	0	None			
O97066	40815	FBgn0262801	twr	90701	23400	SEC11C	9	8.719	high	Yes	Yes	Compara, Homologene, Inparanoid, OMA, OrthoDB, orthoMCL, Phylome, RoundUp, TreeFam
O97066	40815	FBgn0262801	twr	23478	17718	SEC11A	5	4.748	moderate	No	Yes	Compara, Inparanoid, orthoMCL, Phylome, RoundUp
Q9V449	44482	FBgn0028416	Met75Ca				0	0	None			
Q9V449	44481	FBgn0028415	Met75Cb				0	0	None			

Supporting Table S4. Proteins found to be increasingly ubiquitinated [*vs*. control RU486(-) flies] in flies' somatic tissues after inducible (for 7 days) ubiquitous CncC/Nrf2 overexpression (nano-LC-ESI-MS/MS proteomics analysis).

Uniprot_Acc	Fly GeneID	FlyBaseID	Fly Symbol	GO- Biological Process (UniProt; top 2 terms)	GO- Biological Process (UniProt; top 2 terms)
A8JRC2	43102	FBgn0039358	CG5028		
E1JHR5	33351	FBgn0000579	Eno	glycolytic process	
O62619	42620	FBgn0003178	PyK	glycolytic process	
P00408	19893535	FBgn0013675	COX2	mitochondrial electron transport, cytochrome c to oxygen	
Q0E8X7	246602	FBgn0250838	CG30415	photoreceptor cell maintenance	rhodopsin biosynthetic process
Q94920	34500	FBgn0004363	porin	ion transport	mitochondrial transport
Q9VEB1	42185	FBgn0262559	Mdh2	activation of cysteine-type endopeptidase activity involved in apoptotic process	carbohydrate metabolic process
Q9VHH1	41097	FBgn0037664	CG8420	multicellular organism reproduction	
Q9VHJ8	41067	FBgn0037643	skap	mitotic spindle organization	tricarboxylic acid cycle
Q9VMB9	33918	FBgn0031830	CoVb	mitochondrial electron transport, cytochrome c to oxygen	
Q9VU58	50286	FBgn0040813	Nplp2	humoral immune response	neuropeptide signaling pathway

Supporting Table S5. Human orthologs of fly proteins identified to be increasingly ubiquitinated after inducible CncC/Nrf2 overexpression (nano-LC-ESI-MS/MS proteomics analysis).

Uniprot_Acc_Fly Search Term	Fly GeneID	FlyBaseID	Fly Symbol	Human GeneID	HGNCID	Human Symbol	DIOPT Score	Weighted Score	Rank	Best Score	Best Score Reverse	Prediction Derived From
A8JRC2	43102	FBgn0039358	CG5028	3421	5386	IDH3G	9	8.666	high	Yes	Yes	Compara, Homologene, Inparanoid, Isobase, OMA, OrthoDB, orthoMCL, Phylome, TreeFan
A8JRC2	43102	FBgn0039358	CG5028	3420	5385	IDH3B	3	2.904	low	No	No	OrthoDB, orthoMCL, RoundUp
A8JRC2	43102	FBgn0039358	CG5028	3419	5384	IDH3A	1	1.001	low	No	No	OrthoDB
E1JHR5	33351	FBgn0000579	Eno	2023	3350	ENO1	9	8.719	high	Yes	Yes	Compara, Homologene, Inparanoid, OMA, OrthoDB, orthoMCL, Phylome, RoundUp, TreeFar
E1JHR5	33351	FBgn0000579	Eno	2026	3353	ENO2	8	7.719	moderate	No	Yes	Compara, Inparanoid, OMA, OrthoDB, orthoMCL, Phylome, RoundUp, TreeFan
E1JHR5	33351	FBgn0000579	Eno	2027	3354	ENO3	8	7.719	moderate	No	Yes	Compara, Inparanoid, OMA, OrthoDB, orthoMCL, Phylome, RoundUp, TreeFan
E1JHR5	33351	FBgn0000579	Eno	387712	31670	ENO4	1	0.91	low	No	Yes	Phylome
E1JHR5	33351	FBgn0000579	Eno	128178	14341	EDARADD	1	0.91	low	No	Yes	Phylome
O62619	42620	FBgn0003178	PyK	5315	9021	PKM	9	8.719	high	Yes	Yes	Compara, Homologene, Inparanoid, OMA, OrthoDB, orthoMCL, Phylome, RoundUp, TreeFar
O62619	42620	FBgn0003178	PyK	5313	9020	PKLR	7	6.709	moderate	No	Yes	Compara, Inparanoid, OrthoDB, orthoMCL, Phylome, RoundUp, TreeFan
P00408	19893535	FBgn0013675	COX2	4513	7421	COX2	7	6.819	high	Yes	Yes	Compara, Inparanoid, OMA, OrthoDB, Phylome, RoundUp, TreeFan
Q0E8X7	246602	FBgn0250838	CG30415				0	0	None			
Q94920	34500	FBgn0004363	porin	7417	12672	VDAC2	9	8.719	high	Yes	Yes	Compara, Homologene, Inparanoid, OMA, OrthoDB, orthoMCL, Phylome, RoundUp, TreeFar
Q94920	34500	FBgn0004363	porin	7416	12669	VDAC1	8	7.719	moderate	No	Yes	Compara, Inparanoid, OMA, OrthoDB, orthoMCL, Phylome, RoundUp, TreeFan
Q94920	34500	FBgn0004363	porin	7419	12674	VDAC3	8	7.668	moderate	No	Yes	Compara, Inparanoid, Isobase, OMA, orthoMCL, Phylome, RoundUp, TreeFan
Q9VEB1	42185	FBgn0262559	Mdh2	4191	6971	MDH2	8	7.709	high	Yes	Yes	Compara, Homologene, Inparanoid, OrthoDB, orthoMCL, Phylome, RoundUp, TreeFar
Q9VEB1	42185	FBgn0262559	Mdh2	55342	16462	STRBP	1	1.001	low	No	No	OrthoDB
Q9VEB1	42185	FBgn0262559	Mdh2	23217	29189	ZFR2	1	1.001	low	No	No	OrthoDB
Q9VEB1	42185	FBgn0262559	Mdh2	51663	17277	ZFR	1	1.001	low	No	No	OrthoDB
Q9VEB1	42185	FBgn0262559	Mdh2	3609	6038	ILF3	1	1.001	low	No	No	OrthoDB
Q9VHH1	41097	FBgn0037664	CG8420				0	0	None			
Q9VHJ8	41067	FBgn0037643	skap	8803	11448	SUCLA2	9	8.666	high	Yes	Yes	Compara, Homologene, Inparanoid, Isobase, OMA, OrthoDB, orthoMCL, Phylome, TreeFan
Q9VHJ8	41067	FBgn0037643	skap	8801	11450	SUCLG2	2	1.901	low	No	No	OrthoDB, orthoMCL
Q9VMB9	33918	FBgn0031830	CoVb	1329	2269	COX5B	8	7.719	high	Yes	Yes	Compara, Inparanoid, OMA, OrthoDB, orthoMCL, Phylome, RoundUp, TreeFan
Q9VU58	50286	FBgn0040813	Nplp2				0	0	None			

Supporting Table S6. Differences [*vs.* control RU486(-) flies] of the shown metabolites concentration observed on the NMR spectra of UAS CncC/Gal4^{Tub} or UAS CncC^{RNAi}/Gal4^{Tub} RU486(+) flies (transgenes were induced for 7 days) as depicted by z-score transformation.

	Metabolites	B vs A	D vs C	
1	Leucine	0.7	-0.2	x
2	Isoleucine	0.5	0	
3	Valine	1.1	0	
4	Threonine	2.7	-0.5	x
5	Arginine	0.8	0.5	
6	Glutamine	3.5	-1	x
7	Asparagine	3.1	0	
8	Glutamic Acid[1]			
9	Aspartic Acid	0.9	2.9	
10	Histidine	4	-0.4	x
11	Tyrosine	-0.1	-1.4	
12	Glycine	3.9	0.8	x
13	β-Alanine	-0.2	-0.7	
14	Alanine	-4.2	-1	
15	Proline	-3.6	-3	
16	Taurine	3.2	-1.1	x
17	N-AcetylAsparate (NAA)	0.3	-1.1	x
18	Maltose	-1.4	0.1	x
19	Glucose	-1.1	0.7	x
20	Glucose-1-phosphate	-2.2	0.1	x
21	Trehalose	3	-1	x
22	Adenosine Triphosphate (ATP)	2.1	-0.9	x
23	Adenosine Monophosphate (AMP)	-1.6	-0.5	
24	Nicotinamide adenine dinucleotide (NAD^+)	-0.9	-1.2	
25	Uridine Diphosphate (UDPs)	3.1	3.4	
26	Acetate	-0.3	0.6	x
27	Acetone (Acetoacetate[2])	-0.3	0.2	x
28	Gamma Aminobutyric Acid (GABA)	0.5	0.5	
29	O-Phosphocholine	2.4	1.9	
30	Lactic Acid	-0.6	1.1	x
31	Succinic Acid	-5.2	-3.5	
32	Pyruvic Acid[3]			
33	Fumaric Acid	-0.4	-1.5	
34	Citric Acid	1.5	-1	x
35	Malic Acid	-0.6	-0.9	
36	Formic Acid	-0.8	-0.3	
37	Kynurenic Acidc	-0.7	0.2	x
38	3-HydroxyKynurenine	-0.9	-0.1	x
39	Methionine Sulfoxide	-5	0.2	x
40	Polyunsaturated Fatty Acids	-1	-2.7	
41	Triglycerides	-1.6	-3.2	
42	$R\text{-}CH_3$ Fatty Acids	-1.8	-3.4	
43	Linoleic Acid (18:2)	0.5	-1.6	x
44	Arachidonic Acid (AA; 20:4)	0.5	-0.7	x
45	ω3 Fatty Acids	1.5	-1.1	x

[1] all chemical shifts overlapped with Proline resonances
[2] can not be separated from Acetone
[3] overlapped with Glutamate and Proline resonances

A: UAS CncC/Gal4 RU486^{Tub} RU486(-)
B: UAS CncC/Gal4 RU486^{Tub} RU486(+)
C: UAS CncC^{RNAi}/Gal4 RU486^{Tub} RU486(-)
D: UAS CncC^{RNAi}/Gal4 RU486^{Tub} RU486(+)

x metabolites found to be inverted (*vs.* CncC/Nrf2 overexpressing flies) in CncC/Nrf2^{RNAi} flies

A-D: 6X replicates

Supporting Table S7. Differences [*vs*. control RU486(-) flies] of the shown metabolites concentration observed on the NMR spectra of UAS CncC/ $Gal4^{Tub}$ or UAS CncC, UAS Gys^{RNAi}/$Gal4^{Tub}$ RU486(+) flies (transgenes were induced for 7 days) as depicted by z-score transformation.

	Metabolites	B vs A*	E vs F	
1	Leucine	0.7	2.3	
2	Isoleucine	0.5	1.7	
3	Valine	1.1	3.2	
4	Threonine	2.7	3.9	
5	Arginine	0.8	3.8	
6	Glutamine	3.5	1.2	
7	Asparagine	3.1	1.6	
8	Glutamic Acid[1]			
9	Aspartic Acid	0.9	2.3	
10	Histidine	4	4.2	
11	Tyrosine	-0.1	0.7	x
12	Glycine	3.9	2.9	
13	β-Alanine	-0.2	-1.1	
14	Alanine	-4.2	-1.5	
15	Proline	-3.6	-2	
16	Taurine	3.2	3.1	
17	N-AcetylAsparate (NAA)	0.3	0.8	
18	Maltose	-1.4	-3.2	
19	Glucose	-1.1	-0.7	
20	Glucose-1-phosphate	-2.2	-3.1	
21	Trehalose	3	-3.3	x
22	Adenosine Triphosphate (ATP)	2.1	2.2	
23	Adenosine Monophosphate (AMP)	-1.6	-0.1	
24	Nicotinamide adenine dinucleotide (NAD^+)	-0.9	-0.1	
25	Uridine Diphosphate (UDPs)	3.1	1.6	
26	Acetate	-0.3	2.7	x
27	Acetone (Acetoacetate[2])	-0.3	1.4	x
28	Gamma Aminobutyric Acid (GABA)	0.5	1	
29	O-Phosphocholine	2.4	5.6	
30	Lactic Acid	-0.6	1.5	x
31	Succinic Acid	-5.2	-0.3	
32	Pyruvic Acid[3]			
33	Fumaric Acid	-0.4	0.1	x
34	Citric Acid	1.5	2.6	
35	Malic Acid	-0.6	0.4	x
36	Formic Acid	-0.8	-0.2	
37	Kynurenic Acidc	-0.7	1.5	x
38	3-HydroxyKynurenine	-0.9	0.5	x
39	Methionine Sulfoxide	-5	-1.2	
40	Polyunsaturated Fatty Acids	-1	-1.9	
41	Triglycerides	-1.6	-1.7	
42	$R-CH_3$ Fatty Acids	-1.8	-1.8	
43	Linoleic Acid (18:2)	0.5	-1.5	x
44	Arachidonic Acid (AA; 20:4)	0.5	-0.7	x
45	ω3 Fatty Acids	1.5	-0.6	x

[1] all chemical shifts overlapped with Proline resonances
[2] can not be separated from Acetone
[3] overlapped with Glutamate and Proline resonances

A: UAS CncC/$Gal4^{Tub}$ RU486 (-)
B: UAS CncC/$Gal4^{Tub}$ RU486 (+)
E: UAS CncC, UAS Gys^{RNAi}/$Gal4^{Tub}$ RU486 (-)
F: UAS CncC, UAS Gys^{RNAi}/$Gal4^{Tub}$ RU486 (+)

x metabolites found to be inverted (*vs.* UAS CncC overexpressing flies) in UAS CncC, UAS Gys^{RNAi}/$Gal4^{Tub}$ flies

A-D: 6X replicates; E-F: 7X replicates

*values also shown in Table S6
